# Supplementary material for: Association between practice coding of chronic kidney disease (CKD) in primary care and subsequent hospitalisations and death: a cohort analysis using national audit data
Source: BMJ Open. 2022 Oct 11;12(10):e064513. doi: 10.1136/bmjopen-2022-064513 (PMC9558803; doi:10.1136/bmjopen-2022-064513)
Supplement: Supplementary data [file bmjopen-2022-064513supp001.pdf]

SUPPLEMENTARY MATERIALS

Supplementary Table 1. Outcome definitions

Outcome	Definition	Details	ICD-10 codes
AKI	AKI at admission	Any diagnosis (HES) of AKI recorded in the first episode of care	N17
HF	Admission for HF	A primary diagnosis (HES) of HF recorded in the first episode of care	I50, I11.0, I13, I97.1
CV event	Admission for CV event	A primary diagnosis (HES) of HF, CHD, stroke/TIA, PAD or AAA recorded in the first episode of care	HF (I50, I11.0, I13, I97.1)<br>CHD (I20-I25, I51.6)<br>Stroke/TIA (G45-G46)<br>PAD (I79.0, I79.2, I73.8, I73.9, I74.3, I74.4, I74.5, I70.2)<br>AAA (I71, I74.0)<br>Cerebrovascular disease (I60-I69)
All-cause mortality	All-cause mortality	Any death (ONS)	N/A

Supplementary Table 2. Percentage of practices and patients eligible for analysis after application of sample size eligibility criteria (minimum of 50 CKD cases) and comparison of coding rates in the original database and analysis database, in the CKD population and in subgroups

Population	Total practices	Total patients	Total patients coded	Eligible practices	Eligible patients	Eligible patients coded
All CKD	695	169,002	119,248 (70.6%)	637 (91.7%)	167,208 (98.9%)	117,932 (70.5%)
CKD stage 3a	695	106,981	66,514 (62.2%)	580 (83.5%)	103,615 (96.9%)	64,398 (62.2%)
CKD stages 3b-5	695	62,021	52,734 (85.0%)	477 (68.6%)	56,122 (90.5%)	47,700 (84.5%)
Diabetes	695	42,063	32,099 (76.3%)	362 (52.1%)	33,065 (78.6%)	25,273 (76.4%)
No diabetes	695	126,939	87,149 (68.7%)	605 (87.1%)	124,364 (98.0%)	85,324 (68.6%)

Cleary F, *et al. BMJ Open* 2022; 12:e064513. doi: 10.1136/bmjopen-2022-064513

Supplementary Information 1. Methods for estimating the percentage of CV and HF hospitalisation events that are preventable among CKD patients in practices coding 55% of CKD cases if practice coding improved to 88% (attributable fraction for first events)

In order to estimate the percentage of first hospitalisation events that could be prevented over a period of 3.8 years (median follow-up duration) among patients in practices coding 55% of CKD cases (13th practice coding percentile, lower boundary of sextile 2), if such practices instead coded 88% of CKD cases (83rd practice coding percentile, upper boundary of sextile 5), we adopted the Austin method in [1], which we adapted for use in STATA statistical software. We make an assumption that after adjustment for model covariates, the difference in expected event rates between practice coding groups estimated using the methods listed below is caused by practice coding performance. We followed the following steps to estimate the percentage of first events attributable to lower practice coding (55%), when compared to higher practice coding (88%), for both CV and HF hospitalisations:

1. Fit the fully adjusted Cox regression model for time to first event with 5-knot spline for practice percent coded CKD and other practice covariates (model 2), first centring all continuous covariates to generate a sensible baseline group
2. Estimate the baseline survival function [2] using: “predict s, basesurv”, sort data by analysis time and extract the baseline survival probability at t = 3.8 years (median follow-up)
3. Predict the estimated survival probability at t = 3.8 years for every patient in database assuming 54.8% practice percent coded, based on true values of all covariates except with practice percent coded forced to 54.8%, as follows:
    a. Recode data to set value of practice coding variable to 54.8% coded in all patients. (Spline variable values recoded accordingly.)
    b. Use equation $\hat{S}_i(t) = \hat{S}_0(t)^{\exp(\boldsymbol{x}_i\widehat{\boldsymbol{\beta}})}$ [3] to estimate individual survival probabilities $\hat{S}_i(t)$ at time t = 3.8 years, where $\hat{S}_0(t)$ is the estimated baseline survival at time t and $\boldsymbol{x}_i\widehat{\boldsymbol{\beta}}$ is the individual prediction of the linear predictor evaluated at individual true covariate values $\boldsymbol{x}_i$ (but specified practice percent coded of 54.8%) based on coefficient estimates $\widehat{\boldsymbol{\beta}}$
4. Repeat step 3 for practice percent coded forced to 87.5% coded, to obtain individual predicted survival probabilities at t = 3.8 years assuming 87.5% practice percent coded
5. Estimate the expected number of first events occurring over 3.8 years if all practices coded at 54.8% (with other practice characteristics unchanged) by taking the sum of individual probabilities of an event (1 - $\hat{S}_i(t = 3.8)$, using results from (3b)). Repeat for assumption of all practices coding at 87.5% by taking the sum of individual probabilities of an event (1 - $\hat{S}_i(t = 3.8)$, from (4b)).
6. Using results from step 5, compute the percentage of events preventable among individuals in practices coding 54.8% of CKD cases if practices instead coded 87.5% of CKD cases (attributable fraction) as:

$$\%\ events\ preventable\ in\ practices\ coding\ 54.8\%\ of\ CKD\ cases = \frac{expected\ events\ assuming\ 54.8\%\ coding - expected\ events\ assuming\ 87.5\%\ coding}{expected\ events\ assuming\ 54.8\%\ coding} \times 100\%$$

References:

1. Zhang Z, Ambrogi F, Bokov AF, et al. Estimate risk difference and number needed to treat in survival analysis. *Ann Transl Med* 2018;6(7):120.
2. StataCorp. STATA Survival Analysis Reference Manual Release 17. 2021. StataCorp LLC. https://www.stata.com/manuals/st.pdf. Accessed 17 Feb 2022.
3. Rodriguez, G. Approaches to Survival Modelling. In: Lecture Notes on Generalized Linear Models. Princeton University. 2007. https://data.princeton.edu/wws509/notes/c7.pdf. Accessed 17 Feb 2022

Supplementary Figure 1. Distribution of practice CKD coding performance in the CKD analysis population and in subgroups, with red lines depicting sextile boundaries

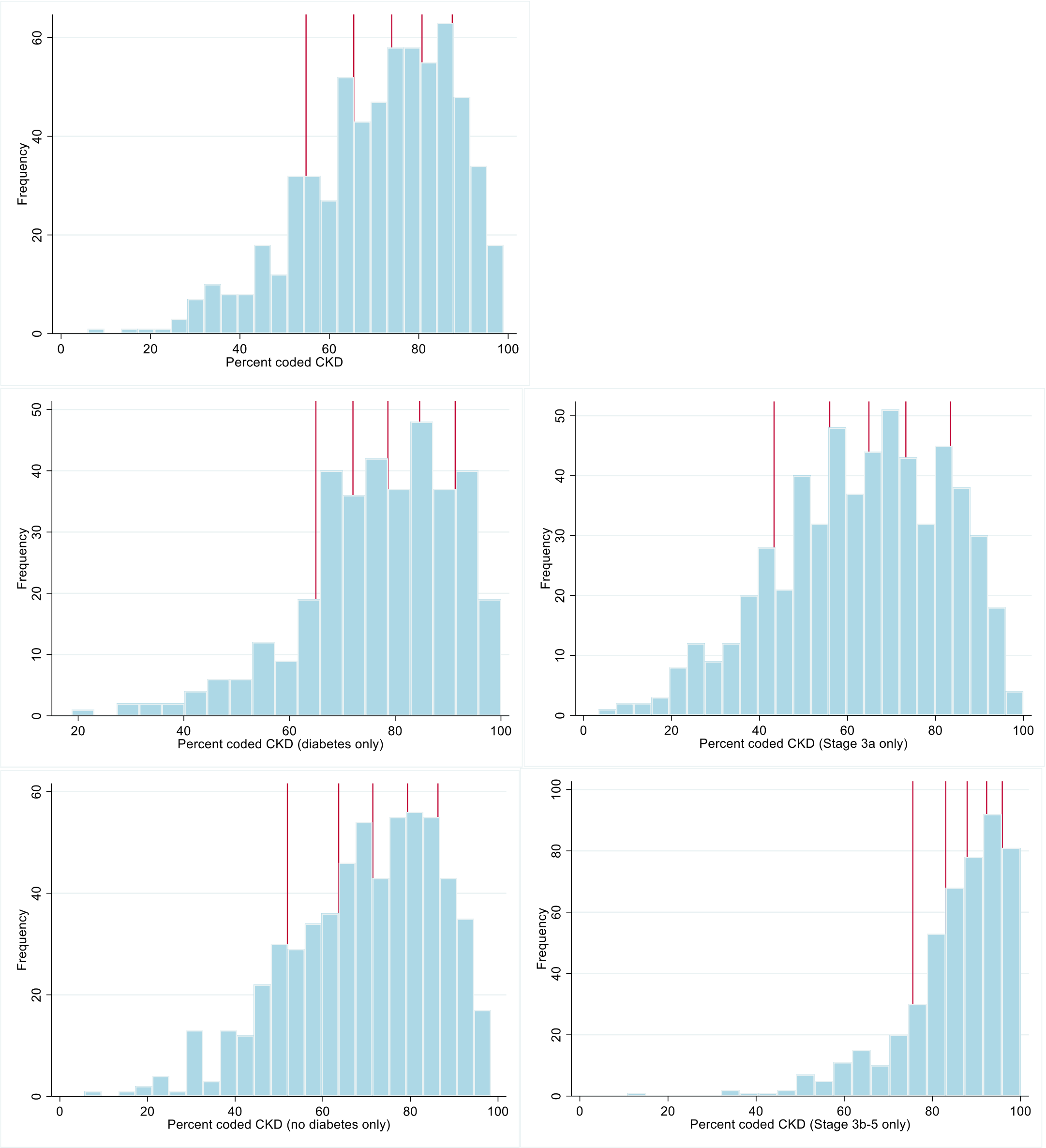

Supplementary Figure 2. Event rates per 100 patient years and 95% confidence intervals, by practice coding sextile in **all CKD** patients (including **recurring events**)

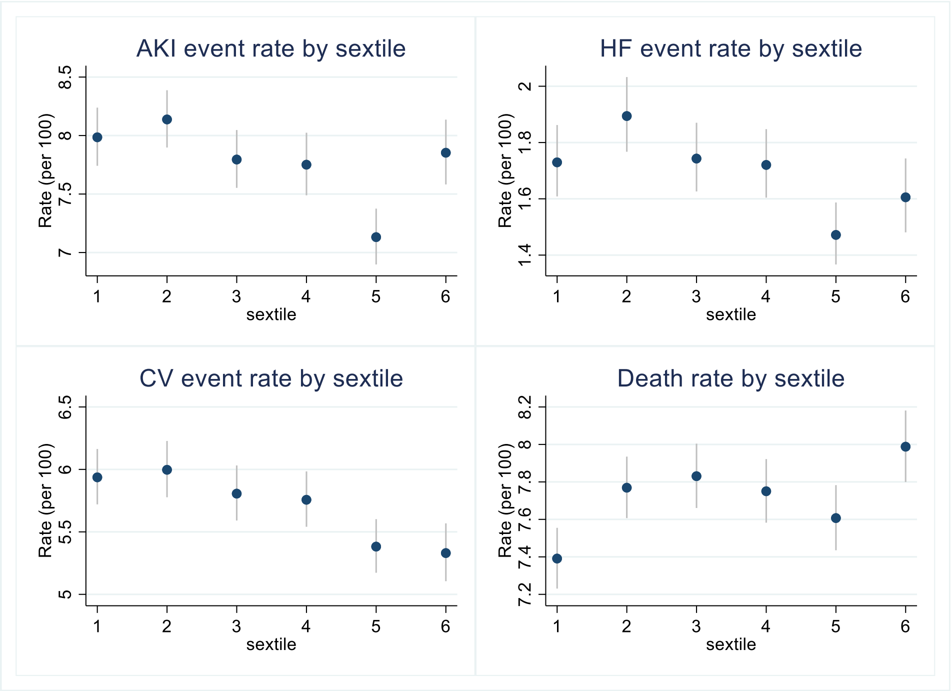

Supplementary Figure 3. Event rates per 100 patient years and 95% confidence intervals, by practice coding sextile in **all CKD** patients (**first events** only)

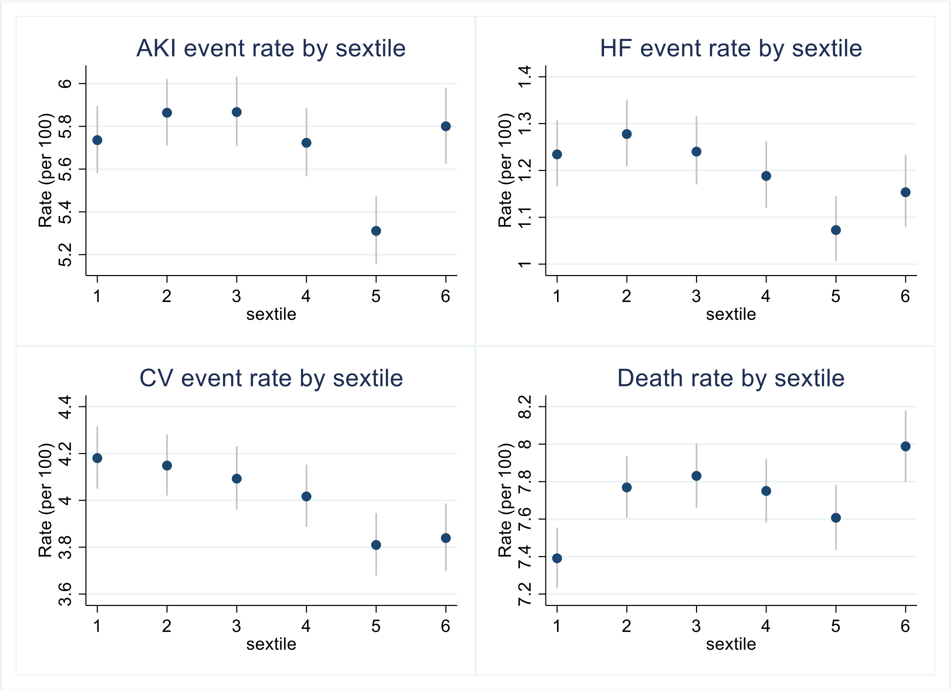

Cleary F, *et al. BMJ Open* 2022; 12:e064513. doi: 10.1136/bmjopen-2022-064513

Supplementary Table 3. Event rates (including recurring events) per 100 patient years and 95% confidence intervals, by practice coding sextile

Practice coding sextile (percent coded CKD)	S1 (<54.8%)	S2 (54.8% - 65.5%)	S3 (65.5% - 73.9%)	S4 (73.9% - 80.7%)	S5 (80.7% - 87.5%)	S6 (≥87.5%)
CV event	N_E = 5766<br>5.94<br>(5.72, 6.16)	N_E = 5980<br>6.00<br>(5.78, 6.23)	N_E = 5326<br>5.81<br>(5.59, 6.03)	N_E = 5340<br>5.76<br>(5.54, 5.98)	N_E = 4677<br>5.38<br>(5.17, 5.60)	N_E = 4037<br>5.33<br>(5.11, 5.57)
HF	N_E = 1680<br>1.73<br>(1.61, 1.86)	N_E = 1889<br>1.89<br>(1.77, 2.03)	N_E = 1599<br>1.74<br>(1.63, 1.87)	N_E = 1596<br>1.72<br>(1.60, 1.85)	N_E = 1279<br>1.47<br>(1.37, 1.59)	N_E = 1216<br>1.61<br>(1.48, 1.74)
AKI	N_E = 7755<br>7.99<br>(7.74, 8.24)	N_E = 8114<br>8.14<br>(7.90, 8.39)	N_E = 7150<br>7.80<br>(7.55, 8.05)	N_E = 7189<br>7.75<br>(7.49, 8.02)	N_E = 6197<br>7.13<br>(6.90, 7.38)	N_E = 5947<br>7.85<br>(7.58, 8.14)
All-cause mortality	N_E = 7985<br>7.39<br>(7.23, 7.55)	N_E = 8612<br>7.77<br>(7.61, 7.93)	N_E = 7994<br>7.83<br>(7.66, 8.00)	N_E = 8014<br>7.75<br>(7.58, 7.92)	N_E = 7362<br>7.61<br>(7.43, 7.78)	N_E = 6740<br>7.99<br>(7.80, 8.18)

N_E = number of events

Supplementary Table 4 Event rates (first events only) per 100 patient years and 95% confidence intervals, by practice coding sextile

Practice coding sextile (percent coded CKD)	S1 (<54.8%)	S2 (54.8% - 65.5%)	S3 (65.5% - 73.9%)	S4 (73.9% - 80.7%)	S5 (80.7% - 87.5%)	S6 (≥87.5%)
CV event	N_E = 3834<br>4.18<br>(4.05, 4.32)	N_E = 3911<br>4.15<br>(4.02, 4.28)	N_E = 3553<br>4.09<br>(3.96, 4.23)	N_E = 3532<br>4.02<br>(3.89, 4.15)	N_E = 3144<br>3.81<br>(3.68, 3.95)	N_E = 2758<br>3.84<br>(3.70, 3.96)
HF	N_E = 1183<br>1.23<br>(1.17, 1.31)	N_E = 1257<br>1.28<br>(1.21, 1.35)	N_E = 1123<br>1.24<br>(1.17, 1.31)	N_E = 1089<br>1.19<br>(1.12, 1.26)	N_E = 922<br>1.07<br>(1.01, 1.14)	N_E = 863<br>1.15<br>(1.08, 1.23)
AKI	N_E = 5228<br>5.74<br>(5.58, 5.89)	N_E = 5480<br>5.86<br>(5.71, 6.02)	N_E = 5055<br>5.87<br>(5.71, 6.03)	N_E = 4993<br>5.72<br>(5.57, 5.88)	N_E = 4359<br>5.31<br>(5.16, 5.47)	N_E = 4135<br>5.80<br>(5.63, 5.98)
All-cause mortality	N_E = 7985<br>7.39<br>(7.23, 7.55)	N_E = 8612<br>7.77<br>(7.61, 7.93)	N_E = 7994<br>7.83<br>(7.66, 8.00)	N_E = 8014<br>7.75<br>(7.58, 7.92)	N_E = 7362<br>7.61<br>(7.43, 7.78)	N_E = 6740<br>7.99<br>(7.80, 8.18)

N_E = number of events

Supplementary Information 2. Methods for sequentially adjusted Cox regression analyses shown in supplementary analyses

Main Cox regression analyses, models 1 and 2, adjusted for practice characteristics are described in the main methods (repeated here for completeness) with results for all studied outcomes shown in the main results. Supplementary figures 4-23 show additional sequential adjustments, demonstrating the role of confounding as different variables were incorporated in analyses, as follows:

- Crude analysis: unadjusted
- Model 1: adjusted for ***practice characteristics reflecting practice risk profile*** (mean age, percent male, median rank of IMD, diabetes prevalence, hypertension prevalence, CVD prevalence)
- Model 1.5: adjusted for ***practice characteristics reflecting practice risk profile*** (model 1 variables), as well as ***practice characteristics of the detected CKD population*** (percent of CKD cases stages 3b-5 ["CKD severity"]), percent admitted for COPD in last 3 years, percent admitted for cancer in last 3 years)
- Model 2: adjusted for ***practice characteristics relating to overall practice risk profile*** and ***of the detected CKD population*** (model 1.5 variables), as well as ***testing biases*** which may result in confounding due to different vintages (i.e. duration of underlying disease) (percent GFR test in last year in diabetes, percent GFR test in last year in CKD, percent of adult population with detected CKD)

As in main analyses, adjusted hazard ratios (HRs) for outcomes with 95% confidence intervals were plotted across the spectrum of practice CKD coding, compared to average practice CKD coding.

Supplementary Figure 4. Hazard ratio splines for time to first **CV event**, compared to median (73.9%) practice percent coded (sequential adjustments)

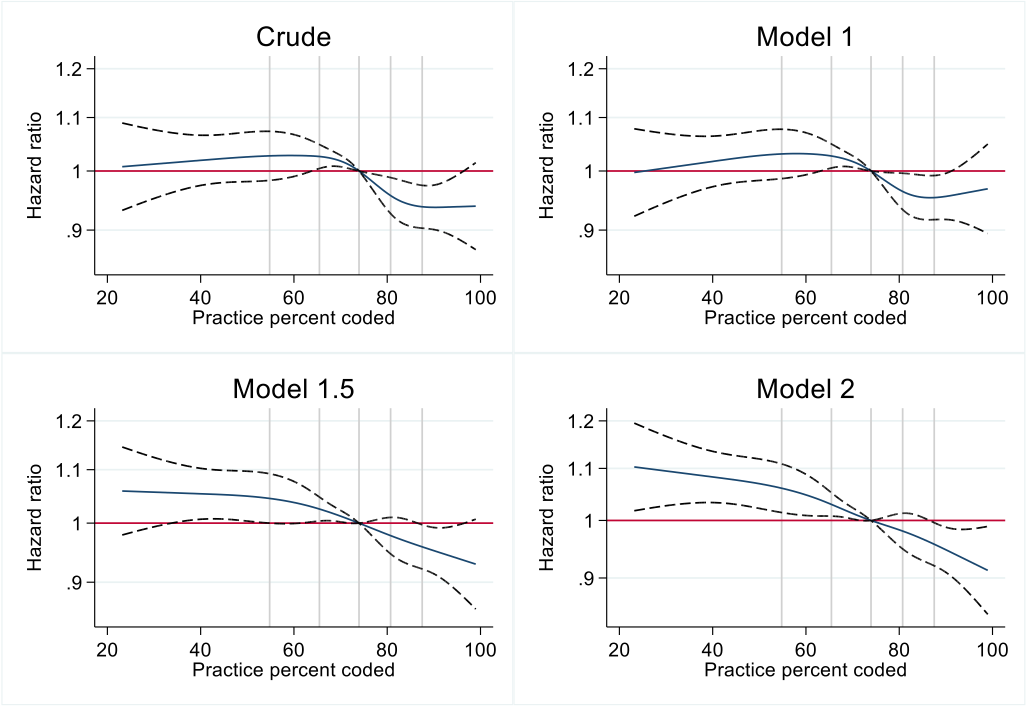

Grey lines depict sextile boundaries

Supplementary Figure 5. Hazard ratio splines for time to first **CV event**, compared to median (64.9%) practice percent coded, in **CKD stage 3a only** (sequential adjustments)

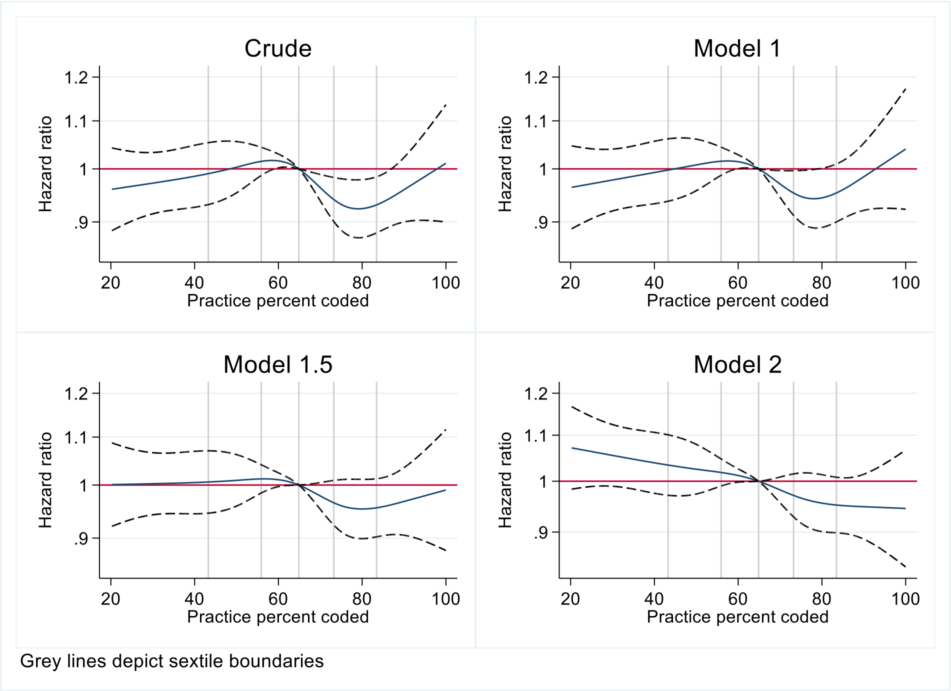

Supplementary Figure 6. Hazard ratio splines for time to first **CV event**, compared to median (87.9%) practice percent coded, in **CKD stages 3b-5 only** (sequential adjustments)

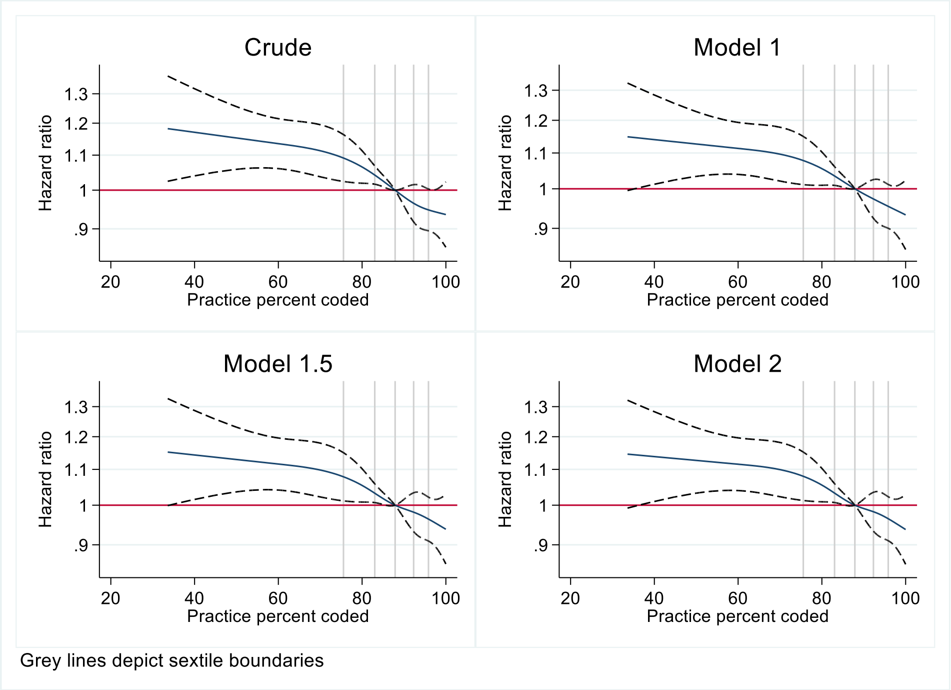

Supplementary Figure 7. Hazard ratio splines for time to first **CV event**, compared to median (78.6%) practice percent coded, in **diabetes only** (sequential adjustments)

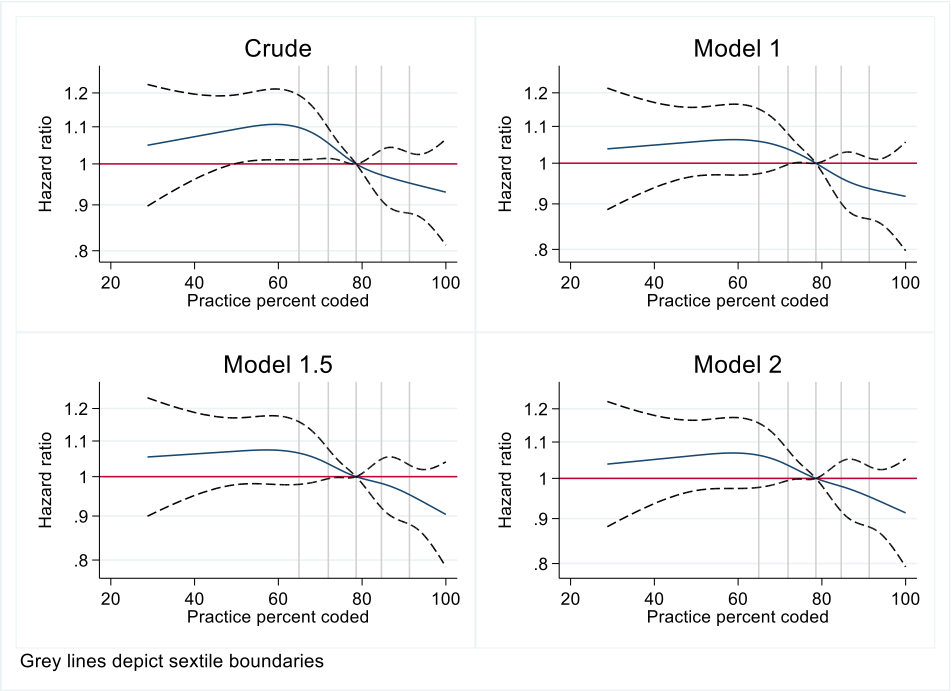

Supplementary Figure 8. Hazard ratio splines for time to first **CV event**, compared to median (71.4%) practice percent coded, in **no diabetes only** (sequential adjustments)

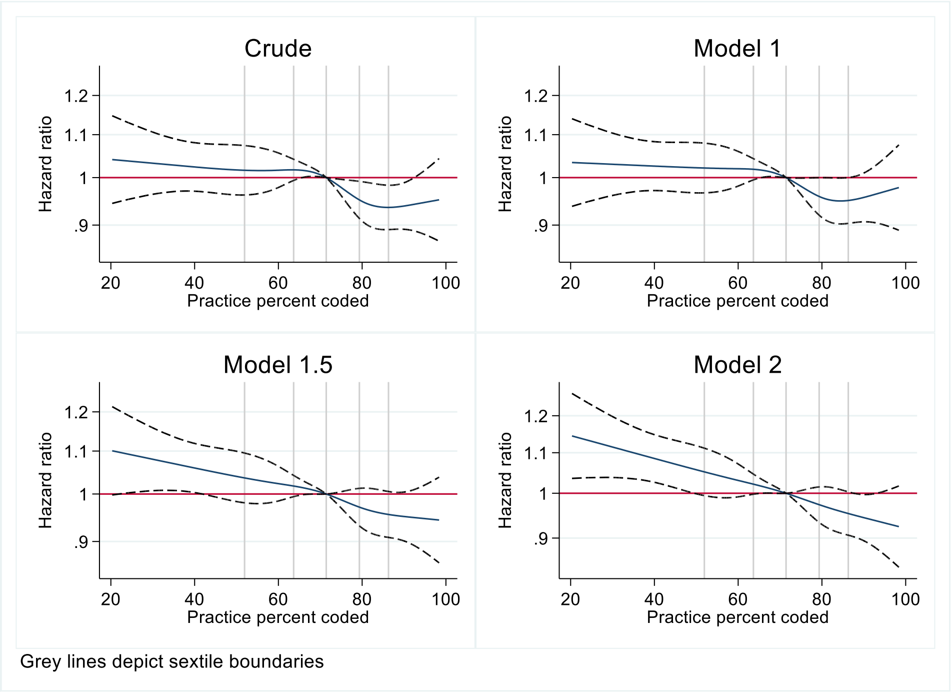

Cleary F, *et al. BMJ Open* 2022; 12:e064513. doi: 10.1136/bmjopen-2022-064513

Supplementary Figure 9. Hazard ratio splines for time to first **HF** event, compared to median (73.9%) practice percent coded (sequential adjustments)

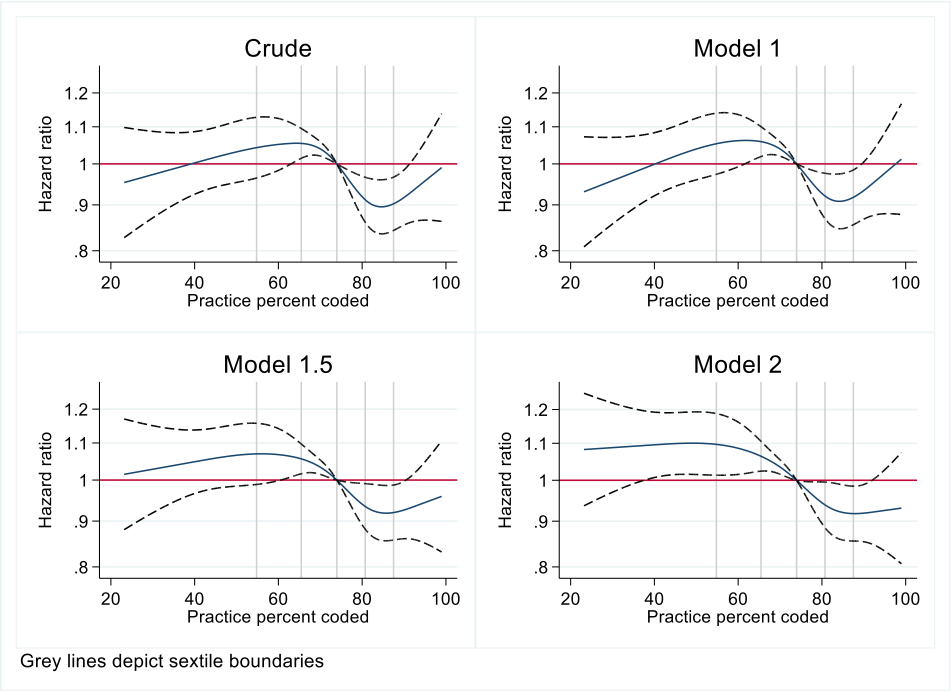

Supplementary Figure 10. Hazard ratio splines for time to first **HF** event, compared to median (64.9%) practice percent coded, in **CKD stage 3a only** (sequential adjustments)

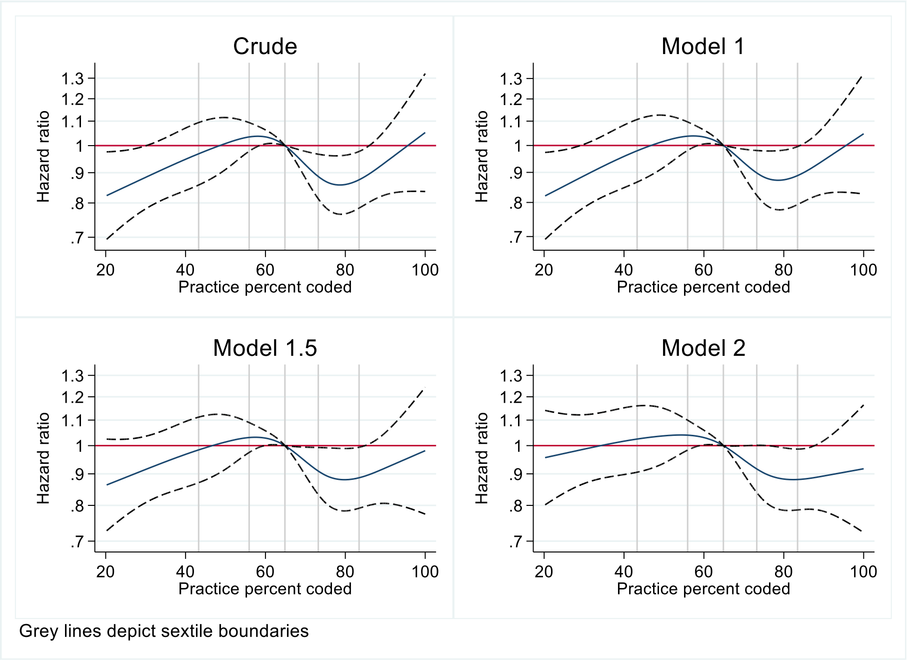

Supplementary Figure 11. Hazard ratio splines for time to first **HF** event, compared to median (87.9%) practice percent coded, in **CKD stages 3b-5** only (sequential adjustments)

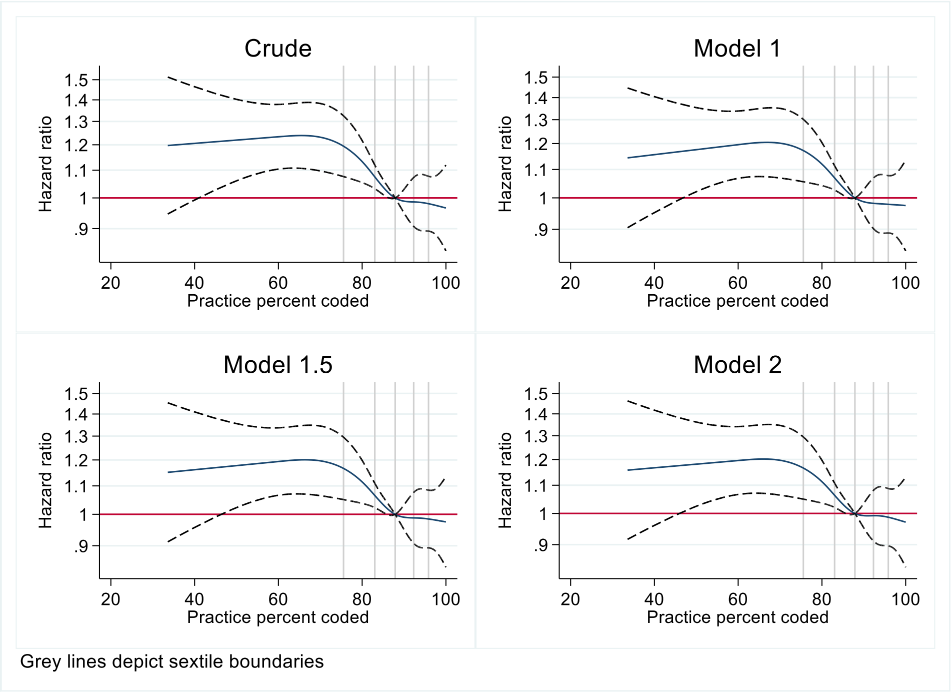

Supplementary Figure 12. Hazard ratio splines for time to first **HF** event, compared to median (78.6%) practice percent coded, in **diabetes only** (sequential adjustments)

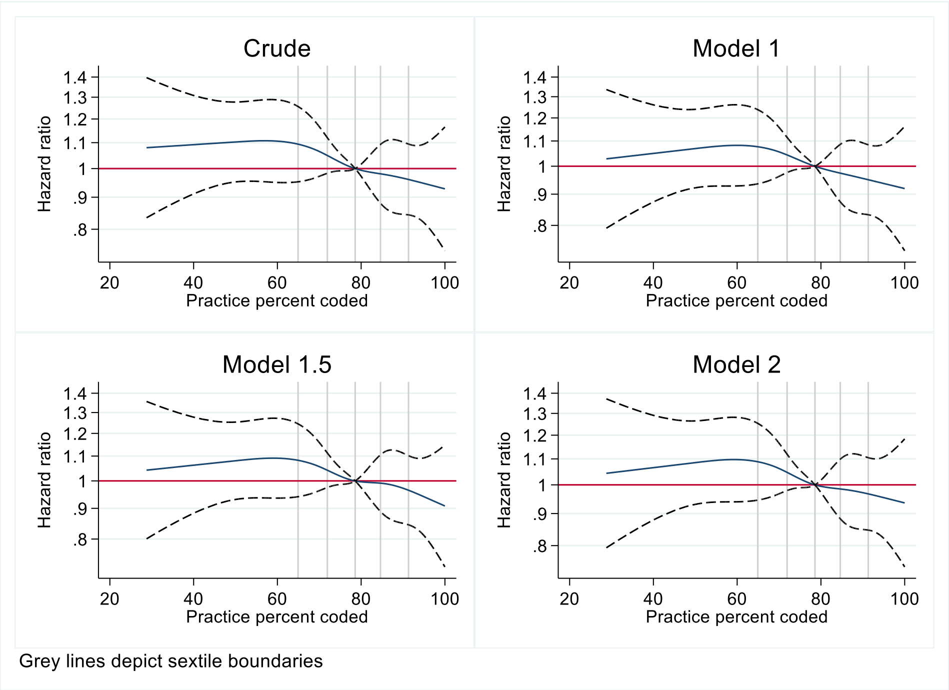

Supplementary Figure 13. Hazard ratio splines for time to first **HF** event, compared to median (71.4%) practice percent coded, in **no diabetes only** (sequential adjustments)

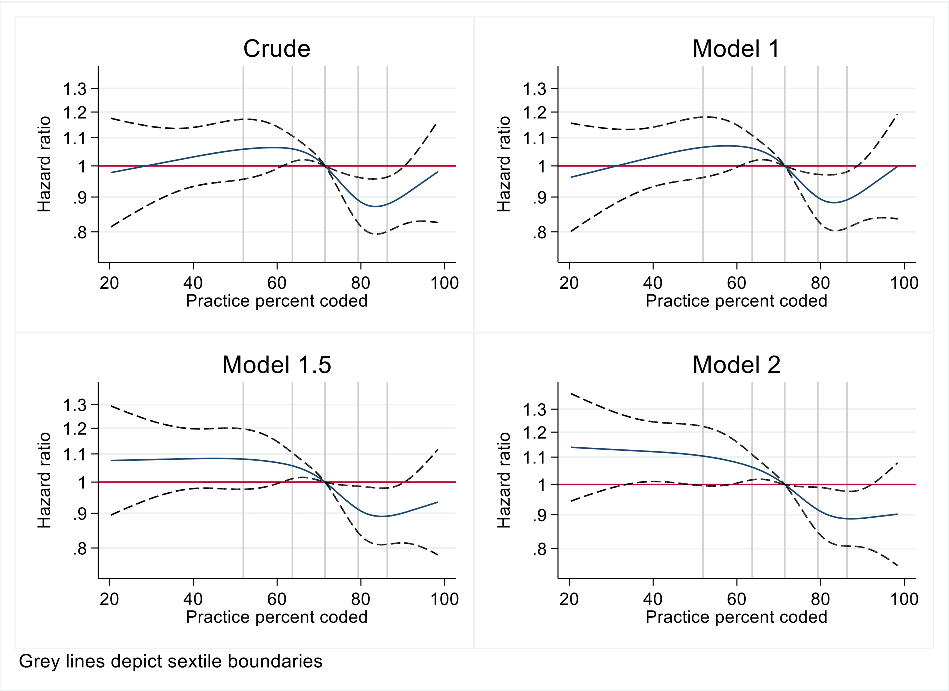

Cleary F, *et al. BMJ Open* 2022; 12:e064513. doi: 10.1136/bmjopen-2022-064513

Supplementary Figure 14. Hazard ratio splines for time to first **AKI** event, compared to median (73.9%) practice percent coded (sequential adjustments)

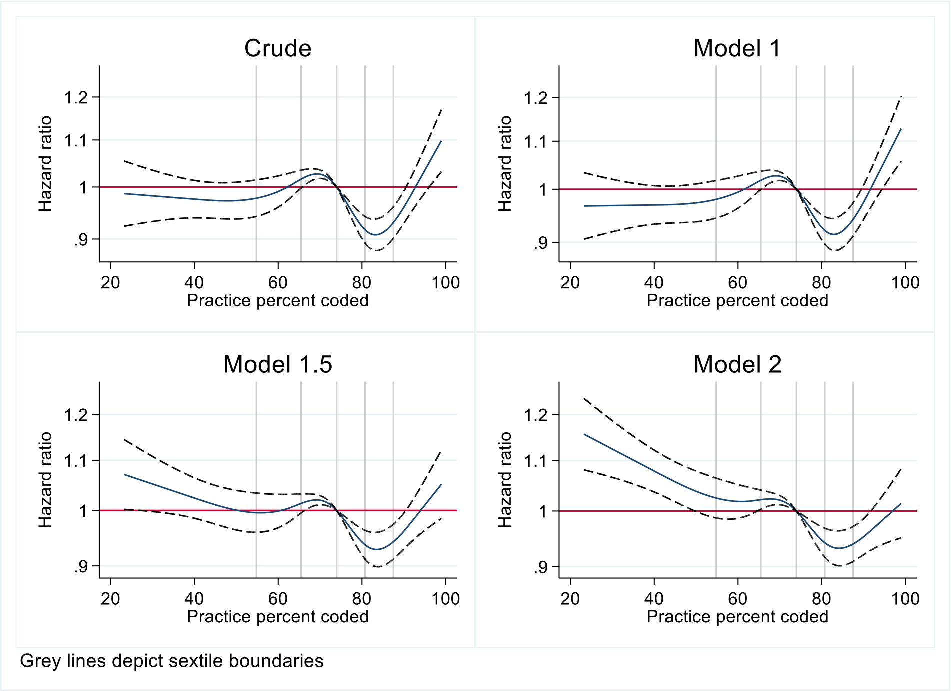

Supplementary Figure 15 Hazard ratio splines for time to first **AKI** event, compared to median (64.9%) practice percent coded, in **CKD stage 3a only** (sequential adjustments)

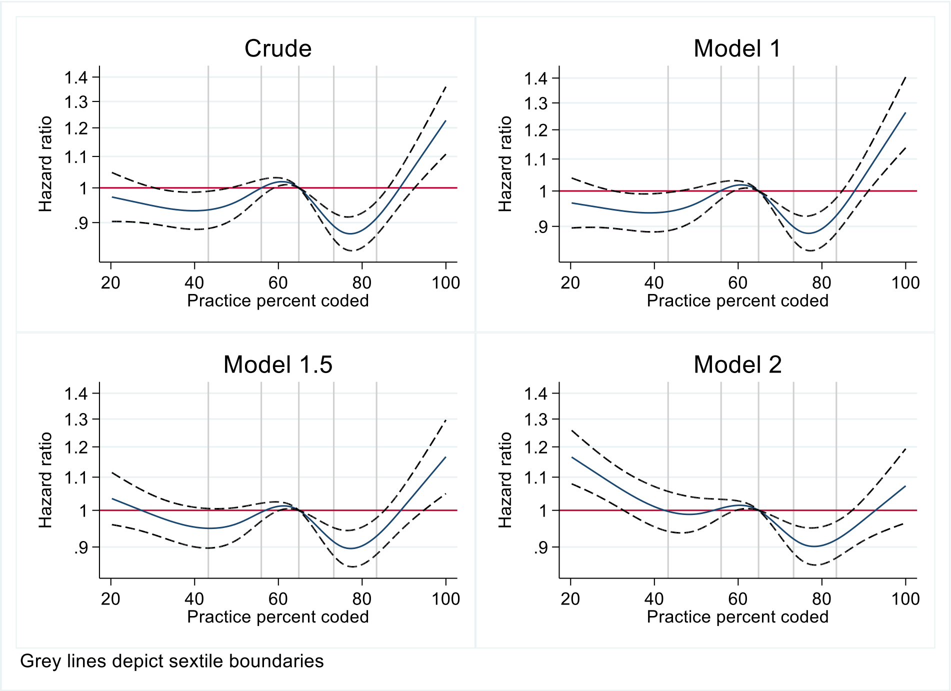

Supplementary Figure 16. Hazard ratio splines for time to first **AKI** event, compared to median (87.9%) practice percent coded, in **CKD stages 3b-5 only** (sequential adjustments)

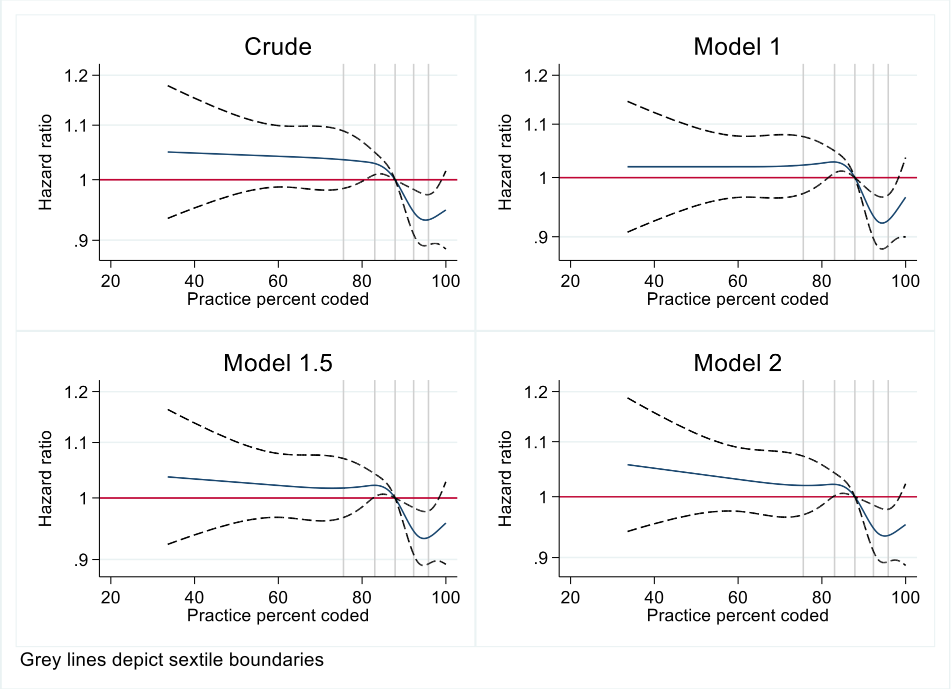

Supplementary Figure 17. Hazard ratio splines for time to first **AKI** event, compared to median (78.6%) practice percent coded, in **diabetes only** (sequential adjustments)

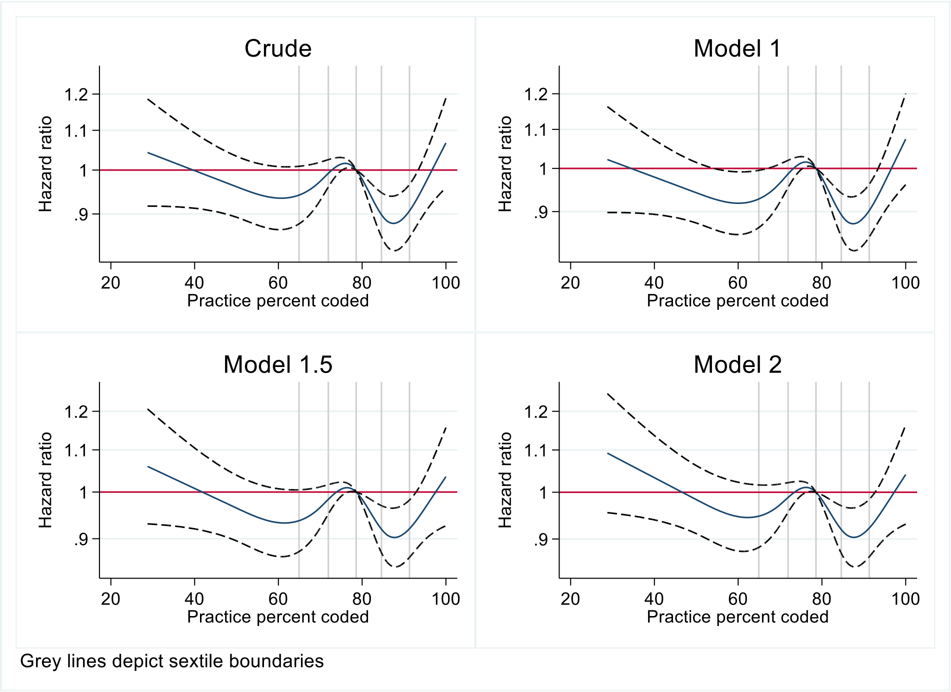

Supplementary Figure 18. Hazard ratio splines for time to first **AKI** event, compared to median (71.4%) practice percent coded, in **no diabetes only** (sequential adjustments)

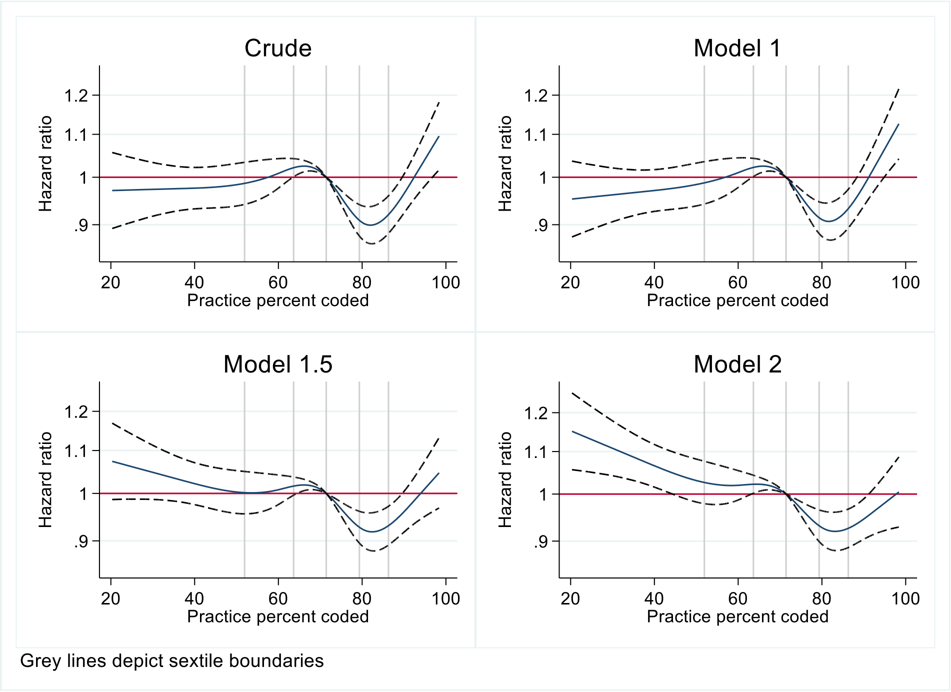

Supplementary Figure 19. Hazard ratio splines for time to **death**, compared to median (73.9%) practice percent coded (sequential adjustments)

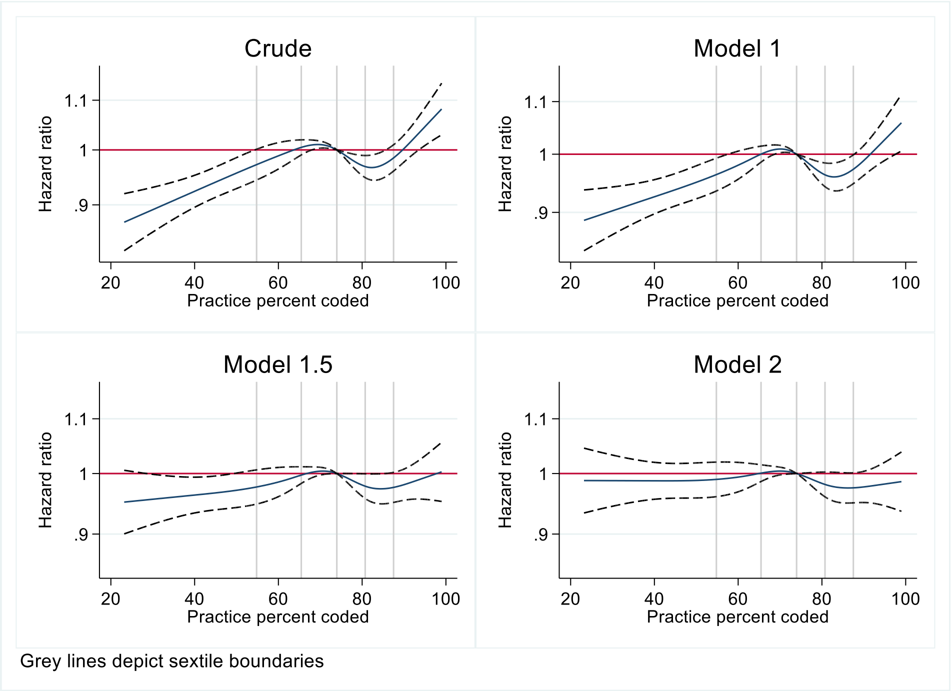

Supplementary Figure 20. Hazard ratio splines for time to **death**, compared to median (64.9%) practice percent coded, in **CKD stage 3a only** (sequential adjustments)

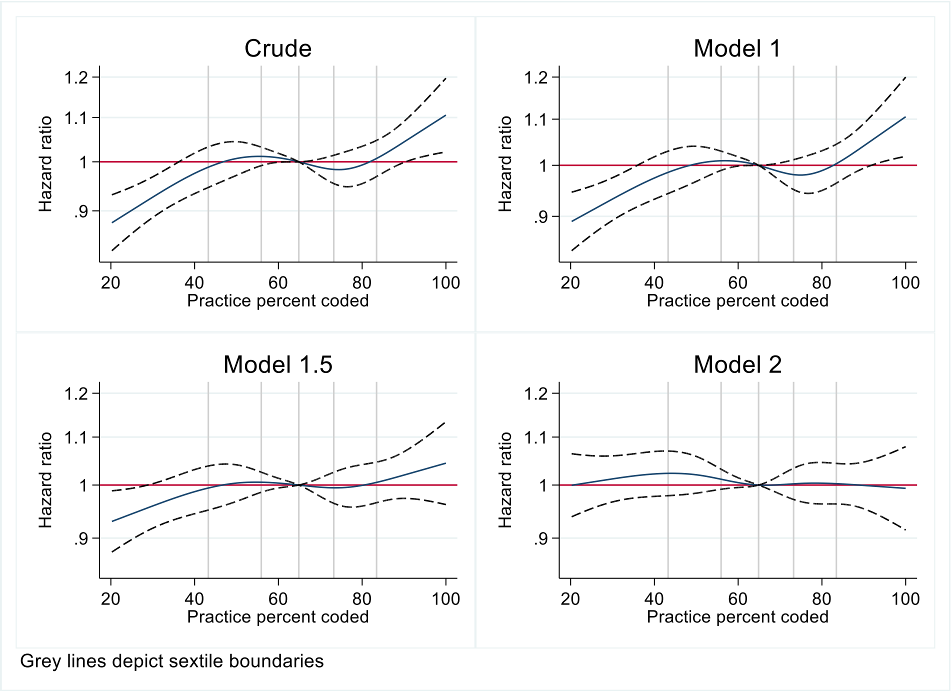

Supplementary Figure 21. Hazard ratio splines for time to **death**, compared to median (87.9%) practice percent coded, in **CKD stages 3b-5 only** (sequential adjustments)

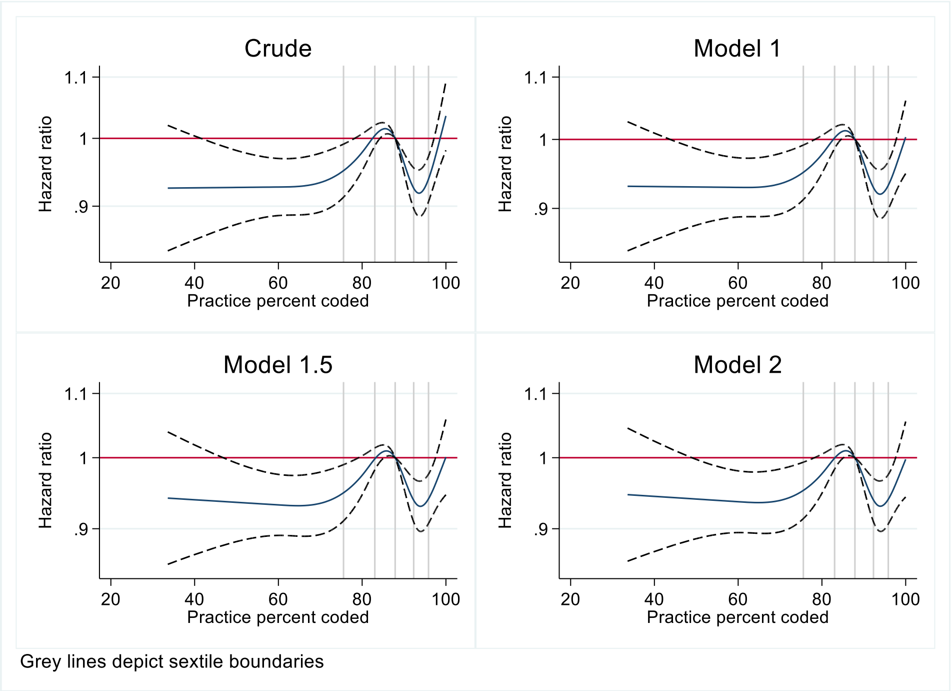

Supplementary Figure 22. Hazard ratio splines for time to **death**, compared to median (78.6%) practice percent coded, in **diabetes only** (sequential adjustments)

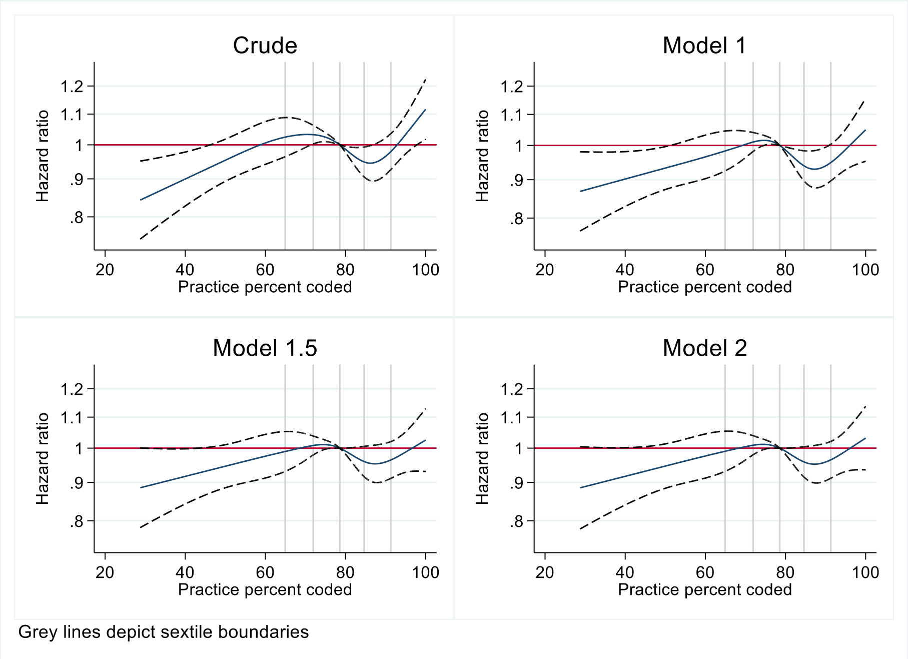

Supplementary Figure 23. Hazard ratio splines for time to **death**, compared to median (71.4%) practice percent coded, in **no diabetes** only (sequential adjustments)

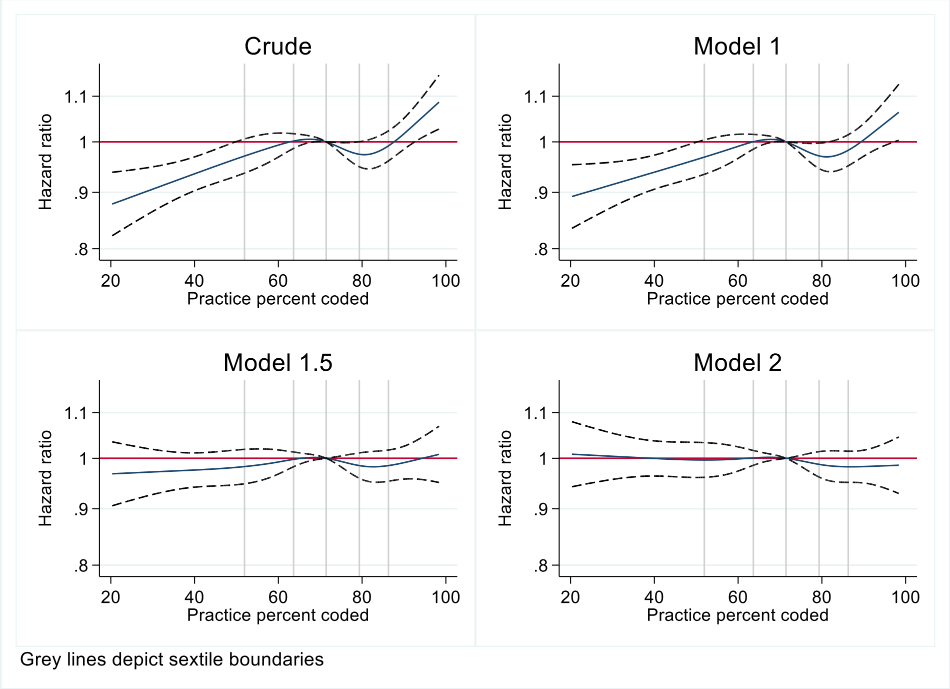

Supplementary Table 5. Fully adjusted hazard ratios (95% CI) per 10% increase in practice percent coded CKD for each outcome analysed, overall and by subgroup

	CV events	HF	Death
**All CKD**	0.973 (0.955, 0.991)*	0.936 (0.905, 0.967)*	0.999 (0.986, 1.011)
**CKD stage 3a**	0.985 (0.964, 1.007)	0.950 (0.911, 0.992)*	N/A**
**CKD stage 3b-5**	0.935 (0.893, 0.980)*	0.913 (0.846, 0.986)*	N/A**
**Diabetes**	0.963 (0.921, 1.006)	0.975 (0.906, 1.049)	N/A**
**No diabetes**	0.970 (0.948, 0.991)*	0.915 (0.879, 0.953)*	N/A**

Analysis not performed for outcomes and subgroups where assumption of linearity not supported by visual checks. (Descriptive likelihood ratio tests confirmed improved fit for linear term over 5-knot spline model in model 2 adjusted analyses, based on p-value threshold of 0.05, for all reported results, providing further justification for linearity assumption.)
Example interpretation: An adjusted hazard ratio of 0.973 for CV events represents a 6.4% reduction in the rate of CV hospitalisations for each 10% increase in practice CKD coding.

Supplementary Figure 24. Hazard ratio slope estimates (and 95% CI) for CV hospitalisations, HF hospitalisations and deaths, assuming linear differences in practice CKD coding performance compared to median practice coding, in the overall CKD population and within subgroups. *Analyses were carried out in the middle 4 sextiles of practices only (the two-thirds of most typically performing practices), with adjustment for all practice factors (model 2)*

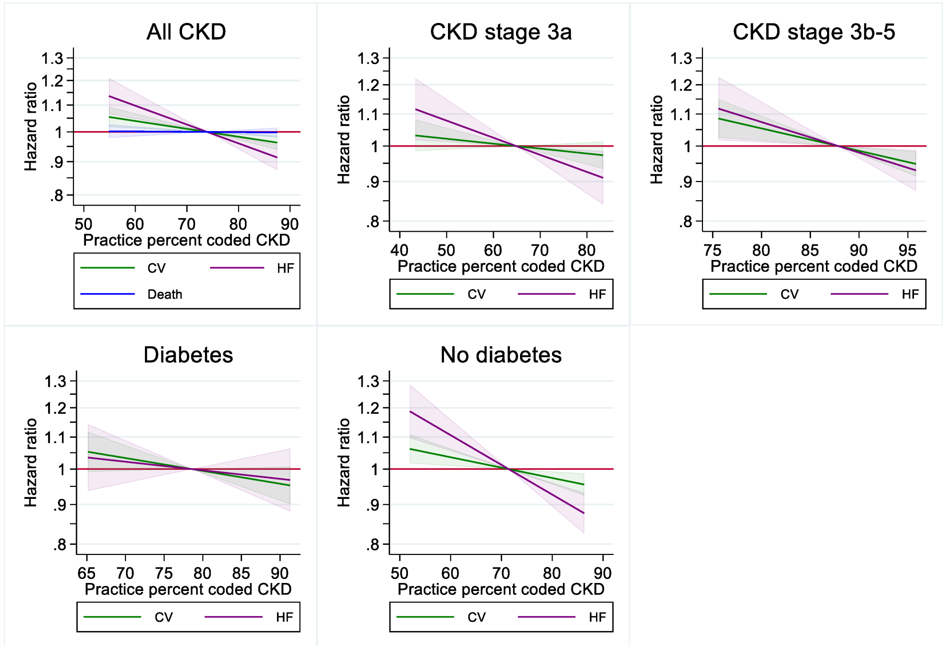

Note: X-scales differ between subgroups, covering middle 4 sextiles within each subgroup

Cleary F, *et al. BMJ Open* 2022; 12:e064513. doi: 10.1136/bmjopen-2022-064513

Supplementary Table 6. Adjusted hazard ratios for the association between practice behaviour variables and **AKI** events, sorted by point estimate

Practice behaviour	HR (95% CI)
Percent ACR/PCR test in last year in CKD (>58.7%)	0.959 (0.935, 0.984)*
Percent flu vaccination in last year in CKD (>78.8%)	0.965 (0.940, 0.990)*
Percent usage of ACEi/ARBs in hypertension (>76.6%)	0.967 (0.944, 0.990)*
Percent usage of statins in CVD (>93.0%)	0.969 (0.944, 0.995)*
Percent usage of statins in diabetes (>84.1%)	0.989 (0.964, 1.015)
Percent pneumococcus vaccination in past 5 years in CKD stages 4-5 (>12.5%)	1.008 (0.985, 1.032)
Percent meeting blood pressure target in last year in CKD (>57.8%)	1.030 (1.004, 1.055)*

Supplementary Table 7. Adjusted hazard ratios for the association between practice behaviour variables and **HF** events, sorted by point estimate

Practice behaviour	HR (95% CI)
Percent usage of ACEi/ARBs in hypertension (>76.6%)	0.935 (0.888, 0.984)*
Percent ACR/PCR test in last year in CKD (>58.7%)	0.951 (0.901, 1.004)
Percent flu vaccination in last year in CKD (>78.8%)	0.959 (0.908, 1.013)
Percent meeting blood pressure target in last year in CKD (>57.8%)	0.976 (0.926, 1.028)
Percent usage of statins in CVD (>93.0%)	0.985 (0.932, 1.042)
Percent pneumococcus vaccination in past 5 years in CKD stages 4-5 (>12.5%)	1.023 (0.973, 1.075)
Percent usage of statins in diabetes (>84.1%)	1.040 (0.984, 1.100)

Supplementary Table 8. Adjusted hazard ratios for the association between practice behaviour variables and **deaths**, sorted by point estimate with descriptive p-values

Practice behaviour	HR (95% CI)
Percent usage of statins in CVD (>93.0%)	0.950 (0.930, 0.970)*
Percent usage of ACEi/ARBs in hypertension (>76.6%)	0.965 (0.947, 0.984)*
Percent usage of statins in diabetes (>84.1%)	0.971 (0.951, 0.991)*
Percent pneumococcus vaccination in past 5 years in CKD stages 4-5 (>12.5%)	1.002 (0.983, 1.020)
Percent flu vaccination in last year in CKD (>78.8%)	1.007 (0.987, 1.027)
Percent meeting blood pressure target in last year in CKD (>57.8%)	1.010 (0.992, 1.030)
Percent ACR/PCR test in last year in CKD (>58.7%)	1.012 (0.992, 1.033)

Cleary F, *et al. BMJ Open* 2022; 12:e064513. doi: 10.1136/bmjopen-2022-064513

Supplementary Figure 25. Results from prior NCKDA analyses, copied from manuscript reference [12]: Hazard ratios for CV events, AKI and mortality in uncoded vs coded patients, adjusted for patient age, sex, and presence of coded diabetes, hypertension and CVD, stratified by latest eGFR measurement, among patients with biochemical evidence of CKD

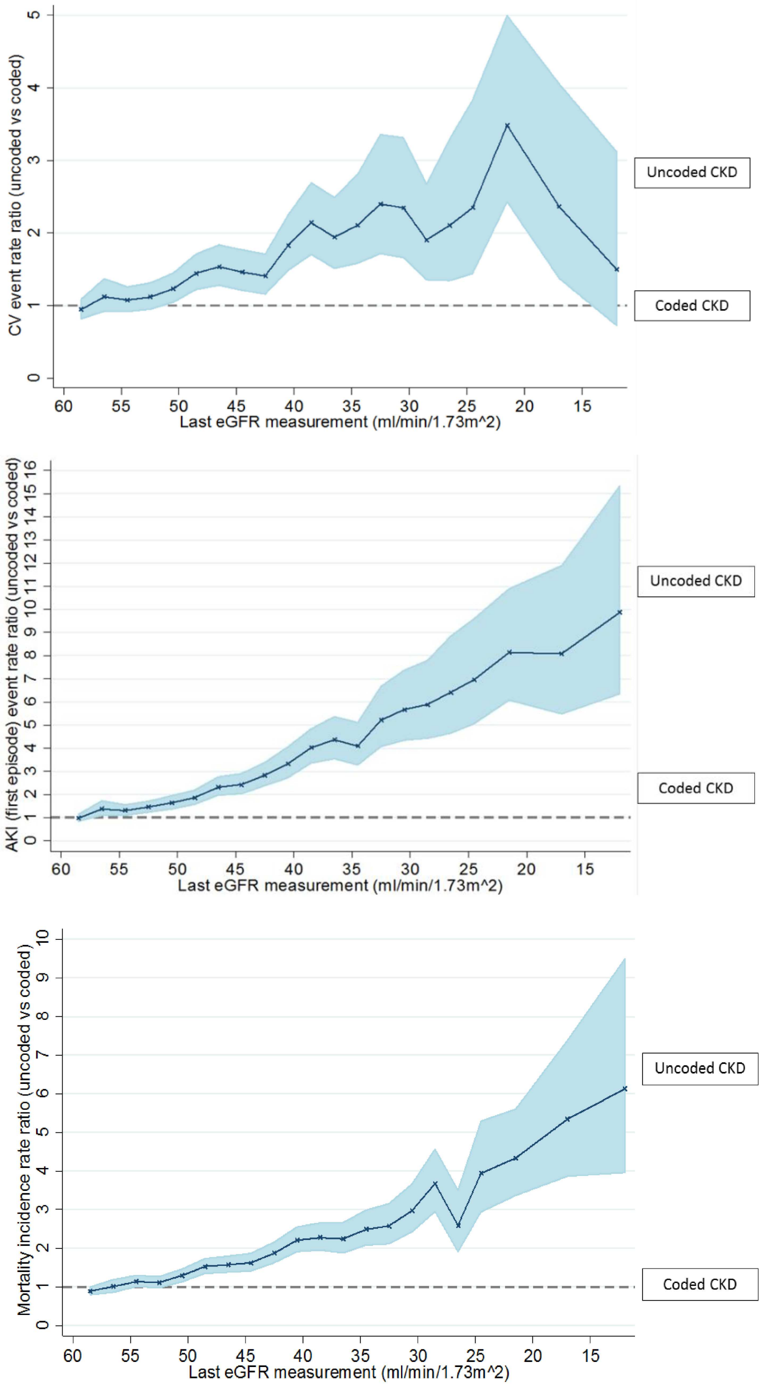

Cleary F, *et al. BMJ Open* 2022; 12:e064513. doi: 10.1136/bmjopen-2022-064513

Supplementary Information 3. Reporting of studies Conducted using Observational Routinely-collected Data (RECORD) checklist

Section/Topic	Item #	Recommendation	Reported on page #
**Title and abstract**	1	(*a*) Indicate the study's design with a commonly used term in the title or the abstract	1, 2
		(*b*) Provide in the abstract an informative and balanced summary of what was done and what was found	2
		RECORD 1.1: The type of data used should be specified in the title or abstract. When possible, the name of the databases used should be included.	2
		RECORD 1.2: If applicable, the geographic region and timeframe within which the study took place should be reported in the title or abstract.	2
		RECORD 1.3: If linkage between databases was conducted for the study, this should be clearly stated in the title or abstract.	2
**Introduction**			
Background/rationale	2	Explain the scientific background and rationale for the investigation being reported	5-6
Objectives	3	State specific objectives, including any prespecified hypotheses	6
**Methods**			
Study design	4	Present key elements of study design early in the paper	6
Setting	5	Describe the setting, locations, and relevant dates, including periods of recruitment, exposure, follow-up, and data collection	6-8
Participants	6	(*a*) Give the eligibility criteria, and the sources and methods of selection of participants. Describe methods of follow-up	6-8
		(*b*) For matched studies, give matching criteria and number of exposed and unexposed	N/A
		RECORD 6.1: The methods of study population selection (such as codes or algorithms used to identify subjects) should be listed in detail. If this is not possible, an explanation should be provided.	7
		RECORD 6.2: Any validation studies of the codes or algorithms used to select the population should be referenced. If validation was conducted for this study and not published elsewhere, detailed methods and results should be provided.	N/A
		RECORD 6.3: If the study involved linkage of databases, consider use of a flow diagram or other graphical display to demonstrate the data linkage process, including the number of individuals with linked data at each stage.	6 (described only, no diagram)

Cleary F, *et al. BMJ Open* 2022; 12:e064513. doi: 10.1136/bmjopen-2022-064513

Variables	7	Clearly define all outcomes, exposures, predictors, potential confounders, and effect modifiers. Give diagnostic criteria, if applicable	7-10; Supplementary Table 1
		RECORD 7.1: A complete list of codes and algorithms used to classify exposures, outcomes, confounders, and effect modifiers should be provided. If these cannot be reported, an explanation should be provided.	7-10; Supplementary Table 1
Data sources/ measurement	8*	For each variable of interest, give sources of data and details of methods of assessment (measurement). Describe comparability of assessment methods if there is more than one group	6-10
Bias	9	Describe any efforts to address potential sources of bias	10-11
Study size	10	Explain how the study size was arrived at	7
Quantitative variables	11	Explain how quantitative variables were handled in the analyses. If applicable, describe which groupings were chosen and why	8-12
Statistical methods	12	(*a*) Describe all statistical methods, including those used to control for confounding	10-12
		(*b*) Describe any methods used to examine subgroups and interactions	11
		(*c*) Explain how missing data were addressed	7-11
		(*d*) If applicable, explain how loss to follow-up was addressed	8, 10
		(*e*) Describe any sensitivity analyses	11
		RECORD 12.1: Authors should describe the extent to which the investigators had access to the database population used to create the study population.	6-7
		RECORD 12.2: Authors should provide information on the data cleaning methods used in the study.	7-10
		RECORD 12.3: State whether the study included person-level, institutional-level, or other data linkage across two or more databases. The methods of linkage and methods of linkage quality evaluation should be provided.	6
**Results**			
Participants	13*	(a) Report numbers of individuals at each stage of study—eg numbers potentially eligible, examined for eligibility, confirmed eligible, included in the study, completing follow-up, and analysed	12, 14, 17
		(b) Give reasons for non-participation at each stage	12, 17; Also see methods p.6-8
		(c) Consider use of a flow diagram	12

		RECORD 13.1: Describe in detail the selection of the persons included in the study (i.e., study population selection) including filtering based on data quality, data availability and linkage. The selection of included persons can be described in the text and/or by means of the study flow diagram.	7, 12
Descriptive data	14*	(a) Give characteristics of study participants (eg demographic, clinical, social) and information on exposures and potential confounders	14
		(b) Indicate number of participants with missing data for each variable of interest	See methods p.6-10
		(c) Summarise follow-up time (eg, average and total amount)	14, 17
Outcome data	15*	Report numbers of outcome events or summary measures over time	17; Also Sup. Figures 2-3, Sup. Table 2
Main results	16	(*a*) Give unadjusted estimates and, if applicable, confounder-adjusted estimates and their precision (eg, 95% confidence interval). Make clear which confounders were adjusted for and why they were included	17-19
		(*b*) Report category boundaries when continuous variables were categorized	14, 17-19
		(*c*) If relevant, consider translating estimates of relative risk into absolute risk for a meaningful time period	17-18
Other analyses	17	Report other analyses done—eg analyses of subgroups and interactions, and sensitivity analyses	17-19
**Discussion**			
Key results	18	Summarise key results with reference to study objectives	19
**Limitations**	19	Discuss limitations of the study, taking into account sources of potential bias or imprecision. Discuss both direction and magnitude of any potential bias	19, 21
		RECORD 19.1: Discuss the implications of using data that were not created or collected to answer the specific research question(s). Include discussion of misclassification bias, unmeasured confounding, missing data, and changing eligibility over time, as they pertain to the study being reported.	20-21
Interpretation	20	Give a cautious overall interpretation of results considering objectives, limitations, multiplicity of analyses, results from similar studies, and other relevant evidence	20-22
Generalisability	21	Discuss the generalisability (external validity) of the study results	20-21
**Other information**			

Funding	22	Give the source of funding and the role of the funders for the present study and, if applicable, for the original study on which the present article is based	23
Accessibility of protocol, raw data, and programming code		RECORD 22.1: Authors should provide information on how to access any supplemental information such as the study protocol, raw data, or programming code.	24; Supplementary materials

*Information should be provided separately for exposed and unexposed groups in cohort studies
