# Supplementary material for: Association between practice coding of chronic kidney disease (CKD) in primary care and subsequent hospitalisations and death: a cohort analysis using national audit data
Source: BMJ Open. 2022 Oct 11;12(10):e064513. doi: 10.1136/bmjopen-2022-064513 (PMC9558803; doi:10.1136/bmjopen-2022-064513)
Supplement: Author's manuscript [file bmjopen-2022-064513.draft_revisions.pdf]

BMJ Open

BMJ Open is committed to open peer review. As part of this commitment we make the peer review history of every article we publish publicly available.

When an article is published we post the peer reviewers' comments and the authors' responses online. We also post the versions of the paper that were used during peer review. These are the versions that the peer review comments apply to.

The versions of the paper that follow are the versions that were submitted during the peer review process. They are not the versions of record or the final published versions. They should not be cited or distributed as the published version of this manuscript.

BMJ Open is an open access journal and the full, final, typeset and author-corrected version of record of the manuscript is available on our site with no access controls, subscription charges or pay-per-view fees (http://bmjopen.bmj.com).

If you have any questions on BMJ Open's open peer review process please email info.bmjopen@bmj.com

BMJ Open

Association between practice coding of chronic kidney disease (CKD) in primary care and subsequent hospitalisations and death: a cohort analysis using national audit data

Journal:	*BMJ Open*
Manuscript ID	bmjopen-2022-064513
Article Type:	Original research
Date Submitted by the Author:	06-May-2022

Complete List of Authors:	Cleary, Faye; London School of Hygiene and Tropical Medicine Department of Non-communicable Disease Epidemiology<br>Kim, Lois; University of Cambridge, Cardiovascular Epidemiology Unit<br>Prieto-Merino, David; London School of Hygiene and Tropical Medicine Department of Non-communicable Disease Epidemiology<br>Wheeler, David; University College London, Department of Renal Medicine<br>Steenkamp, Retha; UK Kidney Association<br>Fluck, Richard; Royal Derby Hospital, Department of Renal Medicine<br>Adlam, David; University of Leicester, Department of Cardiovascular Sciences and NIHR Leicester Biomedical Research Centre<br>Denaxas, S; University College London, Institute of Health Informatics; British Heart Foundation, Data Science Centre<br>Griffith, Kathryn; No affiliation, retired<br>Loud, Fiona; Kidney Care UK<br>Hull, Sally; Queen Mary University of London, Wolfson Institute of Population Health<br>Caplin, Ben; University College London, Department of Renal Medicine<br>Nitsch, Dorothea; London School of Hygiene & Tropical Medicine, Department of Non-Communicable Disease Epidmiology

Keywords:	Chronic renal failure < NEPHROLOGY, EPIDEMIOLOGY, PRIMARY CARE, PUBLIC HEALTH, Quality in health care < HEALTH SERVICES ADMINISTRATION & MANAGEMENT

SCHOLARONE™
Manuscripts

I, the Submitting Author has the right to grant and does grant on behalf of all authors of the Work (as defined in the below author licence), an exclusive licence and/or a non-exclusive licence for contributions from authors who are: i) UK Crown employees; ii) where BMJ has agreed a CC-BY licence shall apply, and/or iii) in accordance with the terms applicable for US Federal Government officers or employees acting as part of their official duties; on a worldwide, perpetual, irrevocable, royalty-free basis to BMJ Publishing Group Ltd ("BMJ") its licensees and where the relevant Journal is co-owned by BMJ to the co-owners of the Journal, to publish the Work in this journal and any other BMJ products and to exploit all rights, as set out in our licence.

The Submitting Author accepts and understands that any supply made under these terms is made by BMJ to the Submitting Author unless you are acting as an employee on behalf of your employer or a postgraduate student of an affiliated institution which is paying any applicable article publishing charge ("APC") for Open Access articles. Where the Submitting Author wishes to make the Work available on an Open Access basis (and intends to pay the relevant APC), the terms of reuse of such Open Access shall be governed by a Creative Commons licence – details of these licences and which Creative Commons licence will apply to this Work are set out in our licence referred to above.

Other than as permitted in any relevant BMJ Author's Self Archiving Policies, I confirm this Work has not been accepted for publication elsewhere, is not being considered for publication elsewhere and does not duplicate material already published. I confirm all authors consent to publication of this Work and authorise the granting of this licence.

For peer review only

Title:

Association between practice coding of chronic kidney disease (CKD) in primary care and subsequent hospitalisations and death: a cohort analysis using national audit data

Authors:

Faye Cleary[1]*, Lois Kim[2], David Prieto-Merino[1], David C Wheeler[3], Retha Steenkamp[4], Richard Fluck[5], David Adlam[6], Spiros Denaxas[7,8], Kathryn Griffith[9], Fiona Loud[10], Sally Hull[11], Ben Caplin[3], Dorothea Nitsch[1]

[1] Department of Non-Communicable Disease Epidemiology, Faculty of Epidemiology and Population Health, London School of Hygiene and Tropical Medicine, Keppel Street, London, WC1E 7HT, UK

[2] Cardiovascular Epidemiology Unit, Department of Public Health and Primary Care, University of Cambridge, Strangeways Research Laboratory, 2 Worts' Causeway, Cambridge, CB1 8RN, UK

[3] Department of Renal Medicine, University College London, Rowland Hill Street, London, NW3 2PF, UK

[4] UK Kidney Association, Brandon House Building 20a1, Southmead Road, Bristol, BS34 7RR, UK

[5] Department of Renal Medicine, Royal Derby Hospital, Uttoxeter Road, Derby, DE22 3NE, UK

[6] Department of Cardiovascular Sciences and NIHR Leicester Biomedical Research Unit, University of Leicester, UK

[7] Institute of Health Informatics, University College London, 222 Euston Road, London, NW1 2DA, UK

[8] British Heart Foundation Data Science Centre, Greater London House, 180 Hampstead Road, London, NW1 7AW, UK

[9] No affiliation, retired

[10] Kidney Care UK, 3 The Windmills, St Mary's Cl, Alton, GU34 1EF, UK

[11] Wolfson Institute of Population Health, Queen Mary University of London, London, UK

*Corresponding author

Email: faye.cleary@lshtm.ac.uk

Word count: 3,995

For peer review only - http://bmjopen.bmj.com/site/about/guidelines.xhtml

ABSTRACT

Objective To examine the association between practice percentage coding of chronic kidney disease (CKD) in primary care with risk of subsequent hospitalisations and death

Design Retrospective cohort study using linked electronic healthcare records

Setting 637 GP practices in England

Participants 167,208 patients with CKD stages 3-5 identified by 2 measures of estimated glomerular filtration rate <60 ml/min/1.73m^2, separated by at least 90 days, excluding those with coded initiation of renal replacement therapy (RRT).

Main outcome measures Hospitalisations with cardiovascular (CV) events, heart failure (HF), acute kidney injury (AKI); all-cause mortality

Results Participants were followed for (median) 3.8 years for hospital outcomes and 4.3 years for deaths. Rates of hospitalisations with CV events and HF were lower in practices with higher percentage CKD coding, most markedly in the two-thirds of most typically performing practices (defined as 17th to 83rd percentile; 55% to 88% coded). Compared to patients in the median performing practice (74% coded), patients in practices coding 55% of CKD cases had a higher rate of CV hospitalisations (HR 1.061 [95% CI 1.015-1.109]) and HF hospitalisations (HR 1.097 [95% CI 1.013-1.187]) and patients in practices coding 88% of CKD cases had a reduced rate of CV hospitalisations (HR 0.957 [95% CI 0.920-0.996]) and HF hospitalisations (HR 0.918 [95% CI 0.855-0.985]). We estimate that 9.0% of CV hospitalisations and 16.0% of HF hospitalisations could be prevented by improving practice CKD coding from 55% to 88%. Analysis of practice behaviours suggests prescription of anti-hypertensives a dominant predictor of a reduction in hospitalisation rates for CKD patients, followed by albuminuria testing and use of statins.

For peer review only

Conclusions Higher levels of CKD coding by GP practices were associated with lower rates of CV and HF events. Increased use of anti-hypertensives and regular albuminuria testing may explain better outcomes, although residual confounding cannot be ruled out.

Keywords

Epidemiology, Chronic renal failure, Primary care, Public health, Quality in health care

For peer review only

For peer review only - http://bmjopen.bmj.com/site/about/guidelines.xhtml

Strengths and limitations of this study

- A large database of 167,208 patients with biochemical evidence of CKD was used for analysis, covering 637 GP practices in England who volunteered to participate in an audit of care and which were representative of the general population in terms of age and sex.
- Risk of confounding due to patient characteristics is reduced by studying the association between practice-level (rather than patient-level) CKD coding and patient-level outcomes, where practice case-mix is not expected to differ with practice coding rates.
- Practice behaviours associated with CKD coding performance that are not believed to occur as a consequence of CKD coding may confound associations but were adjusted for as far as possible.
- Average duration of follow-up between assessment of practice coding performance and end of data collection for outcomes was limited to approximately 4 years; longer term effects of CKD coding may therefore not be captured in this study.

For peer review only

For peer review only - http://bmjopen.bmj.com/site/about/guidelines.xhtml

INTRODUCTION

Chronic kidney disease (CKD) is a growing public health problem.[1-3] Consequences of CKD include cardiovascular (CV) morbidity, acute kidney injury (AKI) and premature mortality, with increasing risks as disease progresses.[4] The burden of CKD and associated healthcare costs are increasing,[5,6] yet recognition of the disease in routine practice is often poor and varies between healthcare providers.[7,8] This may lead to delayed intervention and worsen prognosis in many CKD patients.

In the UK, computer systems used by GP practices allow electronic coding of patient clinical information, enabling consistent and specific recording and ease of access to coded data.[9] The National Institute for Health and Care Excellence (NICE) provides recommendations for regular renal function testing and CKD management in primary care,[10] and the Quality and Outcomes Framework (QOF) provides financial incentives for GPs to maintain a practice register of patients with CKD stages 3-5.[11]

The National Chronic Kidney Disease Audit (NCKDA) conducted in England and Wales in 2015-2016 found that approximately 30% of biochemically confirmed CKD stage 3-5 cases were not given an appropriate CKD code in primary care electronic healthcare records (EHRs).[8] Among patients with biochemical evidence of CKD stage 3-5, those registered with a CKD code had significantly lower rates of CV hospitalisations, AKI and mortality than those without CKD codes.[12] However, analyses only adjusted for age, sex and coded diabetes, hypertension and CV disease, due to limited data availability. The audit report cautioned therefore that a causal association cannot be established for the reported benefits of CKD coding, as results will be affected by confounding by patient health seeking behaviours and unmeasured morbidities. Some have cautioned against overdiagnosis of CKD that may fail to benefit the overall health of the population,[13] and more research is needed to study the benefits of CKD coding.

For peer review only

For peer review only - http://bmjopen.bmj.com/site/about/guidelines.xhtml

In attempt to overcome the issue of unmeasured confounding experienced in original analyses of the NCKDA,[12] we set out to examine the association between completeness of CKD coding of the GP practice at which patients were registered and individual adverse outcomes known to be associated with CKD. We hypothesised that practice CKD coding performance would not be associated with individual patient characteristics within practices (case-mix), thereby removing some potential confounding, and, if appropriately adjusted for practice behaviours, analysis would provide stronger evidence for a causal effect of coding of CKD on outcomes. Additional aims were to explore the role of practice behaviours that may occur as a result of CKD coding in reducing risk of adverse outcomes. Evidence that higher levels of practice CKD coding improved patient outcomes would have important ramifications for GPs and may influence policy to improve recognition of CKD in primary care.

METHODS

Study Design

We carried out a retrospective cohort study using routinely collected EHRs.

Data sources

The NCKDA database holds selected data from the EHRs of 695 GP practices in England and was used to identify a cohort of CKD patients for analysis and to define exposure variables. Data extraction ranged from March 2015 to July 2016 as practices were gradually recruited into the audit, with the majority of practices recruited by July 2015. Details of the audit and data collection strategy are specified elsewhere.[8] The NCKDA database was linked to Hospital Episode Statistics (HES) holding information on all hospital admissions in England, and Office for National Statistics (ONS) mortality data, to follow up patients for adverse outcomes. Linkage was carried out by NHS Digital using NHS number, and hospital record information with pseudo-anonymised linkage IDs were provided for analysis.

For peer review only

For peer review only - http://bmjopen.bmj.com/site/about/guidelines.xhtml

The NCKDA research database has national ethical approval (REC reference: 17/LO/0049), section 251 approval (CAG reference number: 17CAG0058). NCKDA steering group approval[14] and LSHTM ethics approval (reference: 21689) were granted for this analysis.

Study population

The analysis cohort included all adult patients in the NCKDA database extracted from eligible GP practices in England and with biochemical evidence of CKD (hereafter referred to simply as "CKD" and/or "confirmed CKD"), defined as at least 2 records of estimated glomerular filtration rate (eGFR) <60 ml/min/m^2 separated by at least 90 days, with the most recent measure recorded within the last 2 years prior to data extraction and excluding any patients with coded initiation of renal replacement therapy (RRT).

Primary exposure

At the time of data extractions (2015-2016), Read codes were used to electronically record patient findings in GP computer systems.[15] Variables defined based on eGFR use the isotope-dilution mass spectroscopy (IDMS) calibrated Modification of Diet in Renal Disease (MDRD) study equation, the standard GFR-estimating equation in use during the period of data collection.

Practice CKD coding performance was characterised as the percentage of CKD patients in a practice with a CKD stages 3-5 Read code, hereafter referred to as practice CKD coding (performance) or percent coded CKD. Practice CKD coding performance was defined at practice data extraction, which marked the index date for commencement of follow-up for outcomes.

Practices with fewer than 50 total CKD cases were excluded from analysis due to anticipated excess noise in measurement of the primary exposure.

Outcomes

Four outcomes of interest were studied: (i) hospitalisation for CV events, (ii) hospitalisation for heart failure (HF), (iii) hospitalisation with AKI, and (iv) all-cause mortality, defined in **Supplementary Table**

For peer review only - http://bmjopen.bmj.com/site/about/guidelines.xhtml

1. Follow-up began at the time of practice data extraction and was capped at 1st March 2019 for hospital outcomes and 1st September 2019 for deaths.

Practice features

Features of a practice that may confound the association between practice CKD coding and patient adverse outcomes were defined and categorised as: those reflecting overall practice risk profile; practice testing behaviours for CKD; and characteristics of the identified practice CKD population. Practice features that may improve CKD outcomes, some of which may lie on the causal pathway between practice CKD coding and patient adverse outcomes were also defined. Practice percentage variables were defined by summing the number of patients meeting relevant risk factor criteria in each practice and dividing by relevant practice denominators. Practice list size data were used to determine size of the adult population in each practice.

Patient-level risk factors identified in NCKDA data (and used to calculate practice percentages) were defined based on presence of any relevant Read code in the EHR prior to data extraction and included:

- Diabetes – any diabetes code not superseded by a diabetes resolved code
- Hypertension – any hypertension code
- Cardiovascular disease (CVD) – any CVD code
- CKD stages 3b-5 – confirmed CKD and latest eGFR < 45
- Statin use - any statin prescription or contraindication code
- Anti-hypertensive use - any prescription or contraindication code for angiotensin-converting enzyme inhibitors (ACEi) or angiotensin-II receptor blockers (ARBs)
- Blood pressure (BP) targets met - last BP within target range within last year before data extraction: SBP < 130 and DBP < 80 in those with diabetes or last urine albumin to creatinine ratio (ACR) ≥ 700 or last protein to creatinine ratio (PCR) ≥ 100; SBP < 140 and DBP < 90 in all other patients

For peer review only - http://bmjopen.bmj.com/site/about/guidelines.xhtml

- Flu vaccination – any influenza vaccination code in the last year
- Pneumococcal vaccination – any pneumococcal vaccination code in the last 5 years

Additional patient-level risk factors not available in NCKDA data were defined using HES data and included:

- Recent chronic obstructive pulmonary disease (COPD) admission - admission in last 3 years prior to (NCKDA) practice data extraction with a COPD ICD-10 code (J44) as primary diagnosis
- Recent cancer admission - admission in last 3 years prior to (NCKDA) practice data extraction with a cancer ICD-10 code (C00-C97, excluding non-melanoma skin cancers C44) as primary diagnosis

Practice characteristics reflecting overall practice risk profile:

GPs' awareness on how to identify CKD may depend on the overall burden of conditions associated with CKD in their practice. *Practice prevalence of diabetes, hypertension and CVD* were determined by summing the number of adult patients meeting patient-level co-morbidity definitions, with adult population size as denominator. Practice list size data stratified by age and sex were used to determine *mean practice age* and *percent of adults that were male*. Practice deprivation was summarised using the *median rank of the index of multiple deprivation (IMD) score* among all patients extracted from the GP practice (which was limited to patients with CKD risk factors, creatinine assessments or renal codes).

Practice testing behaviours:

Testing behaviours which may impact the patient vintage (i.e. the underlying duration of CKD at detection by the GP) and the types of CKD patients selected for analysis were also defined within practices, including *percentage of diabetes patients with a GFR test in the last year*, *percentage of*

For peer review only - http://bmjopen.bmj.com/site/about/guidelines.xhtml

CKD patients with a GFR test in the last year and *percentage of the practice adult population with confirmed CKD*.

Practice characteristics of the detected CKD population:

Underlying practice morbidity and testing behaviours may impact the types of CKD patients detected and therefore included in analysis. *Percent CKD stages 3b-5*, *percent recent COPD admission* and *percent recent cancer admission* were defined using patient-level risk factor definitions, with number of total CKD cases as denominator.

Practice behaviours that may improve CKD outcomes:

Practice behaviours expected to be related to CKD coding, some of which may be on the causal pathway from CKD to improved outcomes were defined as: *percent usage of ACEi/ARBs in hypertension*, *percent usage of statins in diabetes*, *percent usage of statins in CVD*, *percent meeting blood pressure target in last year in CKD*, *percent ACR/PCR test in last year in CKD*, *percent flu vaccination in last year in CKD*, and *percent pneumococcus vaccination in past 5 years in CKD stages 4-5*. Practice behaviour variables were dichotomised at the median value.

Statistical methods

Baseline characteristics of the study population were summarised by sextile of practice percent coded CKD to determine balance in patient characteristics according to primary exposure. Practice characteristics were also summarised by sextile of practice CKD coding to identify any associations with other practice characteristics.

Main Cox regression analyses:

Cox proportional hazards regression (time to first event) was used to evaluate the association between practice CKD coding and each of the 4 patient outcomes. Hospitalisation outcomes were censored for death. A 5-knot spline was used for the primary exposure, providing flexibility to demonstrate the nature of association between practice coding and outcomes across the spectrum

For peer review only - http://bmjopen.bmj.com/site/about/guidelines.xhtml

of practice CKD coding performance, without overfitting. The following adjustments for practice characteristics (included as continuous covariates) were carried out sequentially:

Model 1: adjusted for ***practice characteristics reflecting overall practice risk profile***

[Primary analysis, planned a priori]

Model 2: adjusted for ***practice characteristics reflecting overall practice risk profile, practice testing behaviours,*** and ***practice characteristics of the detected CKD population***

[Secondary analysis, data-driven]

Adjusted hazard ratio (HR) curves for outcomes with 95% confidence intervals (CIs) were plotted across the spectrum of practice CKD coding, compared to average (median) practice CKD coding. Attributable fractions for the number of (first) events preventable by the median follow-up time among CKD patients in practices at lower coding levels (17th percentile, bottom of sextile 2) if such practices instead coded at higher coding levels (83rd percentile, top of sextile 5) were estimated under assumption of causality following adjusted Cox regression (model 2), detailed in **Supplementary Information 1**.

Additional analyses with a single linear continuous covariate for percent coded CKD were carried out after visual inspection of an approximately linear relationship for some model 2 HR curves, allowing a more convenient clinical interpretation. These models were restricted to sextiles 2-5 of practice coding only (representing the 67% of most averagely performing practices) where linear trends were most apparent. Descriptive likelihood ratio tests were used to assess improvement in model fit using spline terms vs. a single linear covariate.

Subgroup analyses:

Subgroup analyses were carried out by diabetes status and by CKD severity (Stage 3a, Stage 3b-5). Practice percent coded CKD was re-calculated within each subgroup for analysis, since coding behaviour differed substantially between subgroups.

For peer review only - http://bmjopen.bmj.com/site/about/guidelines.xhtml

Analyses of practice behaviours that may improve CKD outcomes:

Further Cox regression analyses aimed to identify practice behaviours associated with improvements in all 4 patient outcomes , with adjustment for all (model 2) confounders as well as ***practice behaviours that may improve CKD outcomes***. Practice CKD coding covariates were excluded from analysis to identify practice factors most predictive of outcomes, regardless of CKD coding performance and not conditional on CKD coding.

Patient and public involvement

Kidney Care UK supported the research questions, grant applications and related record linkage applications of the NCKDA. After NCKDA discontinuation, Kidney Care UK helped with ethics and section 251 permissions to maintain database access for research purposes. A patient representative (Fiona Loud) was involved in the NCKDA from inception, is a co-author and critically reviewed content of this paper.

RESULTS

Data completeness

Of 695 practices in England captured in the NCKDA database, 637 practices (92%) met criteria for analysis (at least 50 CKD cases), covering 99% of all CKD patients from the original database (n=167,208) (**Figure 1**). CKD coding rates did not differ after excluding ineligible practices, overall or by subgroup, but sample sizes were smaller in some subgroups after excluding practices with fewer than 50 CKD cases (**Supplementary Table 2**).

For peer review only - http://bmjopen.bmj.com/site/about/guidelines.xhtml

Patient characteristics

Study population characteristics were generally well balanced between sextiles of practice CKD coding (**Table 1**). There were trends of slightly higher rates of diabetes, hypertension and CVD coding and lower eGFR in patients in the highest coding practices (sextile 6) and slightly lower rates of co-morbidities and higher eGFR in the lowest coding practices (sextile 1), indicating potential differences in either true underlying morbidity or risk factor coding in patients in practices performing at the extremes. Median month of data extraction and resulting follow-up duration were well balanced between sextiles, suggesting good balance in seasonal coverage.

Practice characteristics

Median practice percent coded CKD was 73.9% in the overall CKD population. It was higher in CKD stages 3b-5 (87.9%) than CKD stage 3a (64.8%), and higher in those with diabetes (78.6%) than those without diabetes (71.4%). Coding performance was more variable in early stage CKD and non-diabetic CKD (**Supplementary Figure 1**).

Practice characteristics were generally well balanced in coding sextiles 2-5 (**Table 2**). At the extremes (sextiles 1 and 6), higher coding practices had on average higher prevalence of coded co-morbidities, were more deprived, and performed more regular and complete GFR testing in those at risk. Practice behaviours that may improve CKD outcomes showed trends of improved performance in higher coding practices, across the spectrum of CKD coding. In particular, ACR/PCR testing was substantially higher in higher coding practices, and flu vaccination rates were also markedly higher.

For peer review only - http://bmjopen.bmj.com/site/about/guidelines.xhtml

Table 1. Study population characteristics by practice coding sextile

**Practice coding sextile**	**S1**	**S2**	**S3**	**S4**	**S5**	**S6**
**Practice percent coded CKD**	**<54.8%**	**54.8% - 65.5%**	**65.5% - 73.9%**	**73.9% - 80.7%**	**80.7% - 87.5%**	**≥87.5%**
Number of CKD patients	N = 29,389	N = 30,360	N = 28,248	N = 28,824	N = 26,731	N = 23,656
Coded CKD	13,105 (44.6%)	18,505 (61.0%)	19,744 (69.9%)	22,326 (77.5%)	22,524 (84.3%)	21,728 (91.8%)
Age, mean (SD)	76.7 (11.2)	77.1 (11.0)	77.0 (10.9)	77.2 (10.8)	77.1 (10.7)	77.5 (10.7)
Male	11,686 (39.8%)	12,240 (40.3%)	11,464 (40.6%)	11,709 (40.6%)	10,664 (39.9%)	9,493 (40.1%)
Diabetes	7,013 (23.9%)	7,657 (25.2%)	6,887 (24.4%)	7,288 (25.3%)	6,544 (24.5%)	6,148 (26.0%)
Hypertension	19,475 (66.3%)	20,749 (68.3%)	19,300 (68.3%)	19,798 (68.7%)	18,860 (70.6%)	17,207 (72.7%)
CVD	9,324 (31.7%)	9,945 (32.8%)	9,087 (32.2%)	9,642 (33.5%)	8,819 (33.0%)	8,086 (34.2%)
Biochemical CKD Stage						
3a	18,927 (64.4%)	19,201 (63.2%)	17,938 (63.5%)	18,151 (63.0%)	16,858 (63.1%)	14,774 (62.5%)
3b	8,384 (28.5%)	8,938 (29.4%)	8,254 (29.2%)	8,576 (29.8%)	7,847 (29.4%)	7,089 (30.0%)
4	1,838 (6.3%)	1,937 (6.4%)	1,812 (6.4%)	1,831 (6.4%)	1,796 (6.7%)	1,572 (6.6%)
5	240 (0.8%)	284 (0.9%)	244 (0.9%)	266 (0.9%)	230 (0.9%)	221 (0.9%)
Time since last GFR measure (months), median (IQR)	5.0 (2.0, 9.4)	4.5 (1.8, 8.7)	4.6 (1.9, 8.6)	4.4 (1.8, 8.5)	4.3 (1.8, 8.3)	4.5 (1.9, 8.3)
Prior COPD admission in last 3 years	346 (1.2%)	352 (1.2%)	365 (1.3%)	366 (1.3%)	323 (1.2%)	285 (1.2%)
Prior cancer admission in last 3 years	1,278 (4.3%)	1,332 (4.4%)	1,266 (4.5%)	1,212 (4.2%)	1,122 (4.2%)	1,044 (4.4%)
Follow-up duration (months) for HES outcomes (months), median (IQR)	47 (36, 47)	47 (36, 47)	45 (36, 47)	46 (33, 47)	45 (35, 47)	45 (33, 47)

For peer review only - http://bmjopen.bmj.com/site/about/guidelines.xhtml

Table 2. Practice characteristics by practice coding sextile

**Practice coding sextile**	**S1**	**S2**	**S3**	**S4**	**S5**	**S6**
**Practice percent coded CKD**	**<54.8%**	**54.8% - 65.5%**	**65.5% - 73.9%**	**73.9% - 80.7%**	**80.7% - 87.5%**	**≥87.5%**
Number of practices	N = 106	N = 106	N = 106	N = 107	N = 106	N = 106
Practice percent coded CKD, mean (SD)	44.2% (9.8%)	61.2% (3.5%)	70.0% (2.4%)	77.5% (2.0%)	84.1% (2.2%)	91.7% (3.1%)
Practice characteristics reflecting practice risk profile						
Mean adult age, mean (SD)[M1,M2,CP]	48.9 (4.1)	49.5 (4.7)	49.1 (3.2)	49.2 (3.9)	49.5 (3.8)	49.4 (3.4)
Percent male, mean (SD) [M1,M2,CP]	49.3% (1.9%)	48.6% (2.6%)	49.3% (1.9%)	49.1% (2.0%)	49.3% (2.0%)	49.1% (2.5%)
Practice median rank of IMD, median (IQR) [M1,M2,CP,**]	18,236 (9,574, 23,480)	17,887 (11,835, 22,921)	17,363 (9,987, 24,460)	17,114 (10,392, 21,711)	15,793 (10,946, 21,345)	16,555 (12,742, 22,962)
Diabetes prevalence, mean (SD) [M1,M2,CP]	5.6% (1.3%)	5.9% (1.4%)	6.0% (1.5%)	6.0% (1.5%)	6.0% (1.3%)	6.3% (1.5%)
Hypertension prevalence, mean (SD) [M1,M2,CP]	16.1% (4.1%)	16.8% (3.9%)	16.6% (3.7%)	17.1% (4.1%)	17.6% (3.7%)	18.7% (4.2%)
CVD prevalence, mean (SD) [M1,M2,CP]	5.5% (1.8%)	5.9% (2.4%)	5.8% (1.6%)	6.0% (1.8%)	6.2% (1.6%)	6.4% (1.8%)
Practice testing behaviours						
Percent GFR test in last year in diabetes, mean (SD) [M2,CP]	86.9% (12.8%)	89.8% (5.3%)	89.7% (4.7%)	89.7% (5.2%)	89.7% (10.4%)	90.3% (5.0%)
Median months since last GFR test in diabetes, median (IQR)	4.6 (3.7, 5.4)	4.2 (3.5, 5.0)	4.2 (3.7, 5.1)	4.4 (3.7, 5.1)	3.8 (3.3, 4.7)	4.4 (3.8, 5.1)
Percent GFR test in last year in CKD, mean (SD) [M2,CP]	83.3% (12.7%)	86.6% (4.9%)	87.0% (4.7%)	87.2% (5.6%)	86.6% (10.6%)	88.3% (5.5%)
Median months since last GFR test in CKD, median (IQR)	4.8 (4.1, 5.7)	4.6 (4.1, 5.2)	4.7 (4.0, 5.2)	4.5 (3.8, 5.3)	4.2 (3.6, 5.0)	4.4 (3.9, 5.2)
Biochemical CKD prevalence, mean (SD) [M2,CP]	4.2% (1.9%)	4.0% (1.5%)	3.9% (1.4%)	3.8% (1.5%)	4.0% (1.4%)	4.0% (1.5%)

For peer review only - http://bmjopen.bmj.com/site/about/guidelines.xhtml

Practice characteristics of the detected CKD population						
Percent CKD stages 3b-5, mean (SD) [M2,CP]	37.0% (5.4%)	37.5% (4.9%)	37.5% (4.8%)	38.3% (5.2%)	38.0% (4.3%)	38.0% (5.0%)
Percent recent COPD admission, mean (SD) [M2,CP]	1.2% (1.0%)	1.2% (0.7%)	1.4% (0.9%)	1.3% (1.0%)	1.3% (1.1%)	1.2% (1.0%)
Percent recent cancer admission, mean (SD) [M2,CP]	4.3% (1.7%)	4.4% (1.9%)	4.4% (1.8%)	4.2% (1.8%)	4.1% (1.8%)	4.4% (2.0%)
Practice behaviours that may improve CKD outcomes						
Percent usage of ACE/ARBs in hypertension, mean (SD)[CP]	76.0% (6.2%)	75.5% (6.1%)	77.1% (5.3%)	75.9% (5.9%)	76.5% (4.6%)	78.2% (5.5%)
Percent usage of statins in diabetes, mean (SD) [CP]	82.2% (5.8%)	83.2% (5.8%)	82.9% (5.5%)	84.2% (5.8%)	84.8% (5.3%)	85.0% (5.5%)
Percent usage of statins in CVD, mean (SD) [CP]	91.4% (3.4%)	92.2% (3.5%)	92.8% (2.9%)	93.0% (2.6%)	93.1% (2.6%)	93.0% (3.5%)
Percent meeting blood pressure target in last year in CKD, mean (SD) [CP]	56.8% (7.4%)	57.0% (6.2%)	57.9% (7.0%)	56.8% (7.4%)	58.3% (7.4%)	60.2% (6.7%)
Percent ACR/PCR test in last year in CKD, mean (SD) [CP]	40.1% (13.3%)	49.1% (13.4%)	55.9% (11.9%)	61.3% (13.3%)	62.5% (17.0%)	68.9% (15.7%)
Percent flu vaccination in last year in CKD, mean (SD) [CP]	73.3% (15.4%)	77.0% (7.4%)	77.6% (5.7%)	78.8% (5.6%)	79.3% (9.5%)	80.5% (6.3%)
Percent pneumococcus vaccination in past 5 years in CKD stages 4-5, mean (SD) [CP]	15.3% (14.9%)	18.2% (16.8%)	15.2% (14.5%)	19.1% (17.0%)	17.1% (15.7%)	18.2% (18.6%)

*Prevalence statistics use adult population as denominator
** IMD is ranked for all LSOAs in England (1=worst, 32,844=best); summary statistics are based on IMD rankings of patients extracted from practices only and should be interpreted carefully
[M1] indicates variables included in statistical analysis model 1 (primary analysis)
[M2] indicates variables included in statistical analysis model 2 (secondary analysis)
[CP] indicates variables included in statistical analysis of practice behaviour variables

For peer review only - http://bmjopen.bmj.com/site/about/guidelines.xhtml

Outcomes

Of 167,208 CKD patients identified from the NCKDA database, 563 died before start of follow-up, leaving 166,645 eligible for outcomes analysis. Median follow-up duration was 3.8 years (range 1 day – 3.9 years) for HES outcomes and 4.3 years (range 1 day – 4.4 years) for mortality, with no meaningful differences in follow-up between sextiles. Crude event rates by sextile are shown in **Supplementary Table 3** and **Supplementary Figures 2-3**.

Main adjusted Cox regression analyses

Figure 2 demonstrates how individual patient risks of the 4 studied outcomes differ according to practice CKD coding, compared to a patient in an averagely performing practice. In model 1 analyses, inverted S-shaped HR curves suggest that confounding at the extremes of practice coding may distort the association between practice CKD coding and adverse outcomes. After further adjustments (model 2), curves become flatter (approaching linearity). Wide CIs at lower levels of CKD coding reflect poor precision in HR estimates due to sparse data. (Crude analyses and additional sequential adjustments in the CKD population and subgroups [detailed in **Supplementary Information 2**] are shown in **Supplementary Figures 4-23.)**

There are strong trends, particularly among sextiles 2-5 (55% to 88% coded), of reduced rates of outcomes with improved practice CKD coding in fully adjusted analyses (model 2). Compared to patients in the averagely performing practice (74% coded), patients in practices coding only 55% of CKD cases had a significantly higher rate of CV hospitalisations (HR 1.061 [95% CI 1.015-1.109]) and HF hospitalisations (HR 1.097 [95% CI 1.013-1.187]). Patients in practices coding 88% of CKD cases had a significantly reduced rate of CV hospitalisations (HR 0.957 [95% CI 0.920-0.996]) and HF hospitalisations (HR 0.918 [95% CI 0.855-0.985]), compared to the averagely performing practice. The percentage of preventable events over a period of 3.8 years (median follow-up time) for an improvement in practice coding from 55% to 88% of CKD cases (attributable fraction) was 9.0% for first CV hospitalisations and 16.0% for first HF hospitalisations, under assumption of causality after

For peer review only - http://bmjopen.bmj.com/site/about/guidelines.xhtml

Cox modelling (model 2). Trends of a small reduction in AKI but no substantial change in rate of deaths were also observed as CKD coding increased. (Additional results of analysis of a single linear practice coding term [where appropriate] are shown in **Supplementary Table 4** and **Supplementary Figure 24**.)

Subgroup analyses showed a steeper reduction in CV hospitalisations and HF hospitalisations with increasing practice coding among CKD stages 3b-5 than in CKD stage 3a both in spline regression analyses and those assuming linear effects of practice CKD coding (**Supplementary Figures 5, 6, 10, 11 and 24**).

Practice behaviours analyses

Analysis of the association between practice behaviours and CV hospitalisations adjusted for all confounders showed a significant reduction in rate of CV hospitalisations for CKD patients belonging to practices with greater than average usage (median 76.6%) of ACEi/ARBs in hypertension compared to practices with lower than average usage (HR 0.956 [95% CI 0.929, 0.983]) (**Table 3**). Practice ACR/PCR testing in CKD was also associated with a reduction in rates of CV hospitalisations in CKD (HR 0.968 [95% CI 0.939-0.998]). Results for analyses of AKI, HF and deaths are available in **Supplementary Tables 5 - 7**. In brief, usage of ACEi/ARBs was the most consistently dominant predictor, being strongly associated with a reduction in events across all outcomes. This was followed by ACR/PCR testing, which was associated with a reduction in all outcomes except deaths, and usage of statins in CVD, which was associated with a reduction in rate of CV and AKI events.

For peer review only - http://bmjopen.bmj.com/site/about/guidelines.xhtml

Table 3. Adjusted HRs for the association between practice behaviour variables and CV events, sorted by point estimate

Practice behaviour	HR (95% CI)
Percent usage of ACEi/ARBs in hypertension (>76.6%)	0.956 (0.929, 0.983)*
Percent ACR/PCR test in last year in CKD (>58.7%)	0.968 (0.939, 0.998)*
Percent usage of statins in CVD (>93.0%)	0.972 (0.942, 1.003)
Percent pneumococcus vaccination in past 5 years in CKD stages 4-5 (>12.5%)	0.982 (0.955, 1.010)
Percent meeting blood pressure target in last year in CKD (>57.8%)	0.992 (0.963, 1.028)
Percent usage of statins in diabetes (>84.1%)	0.995 (0.965, 1.026)
Percent flu vaccination in last year in CKD (>78.8%)	0.998 (0.968, 1.028)

Analysis adjusted for practice characteristics: mean age, percent male, median rank of index of multiple deprivation, diabetes prevalence, hypertension prevalence, CVD prevalence, percent of CKD cases at stages 3b-5, percent of CKD patients admitted for COPD in last 3 years, percent of CKD patients admitted for cancer in last 3 years, percent GFR test in last year in diabetes, percent GFR test in last year in CKD, percent of adult population with CKD

DISCUSSION

Higher levels of practice CKD coding were associated with lower rates of hospitalisation for CV and HF events among patients with confirmed CKD, after adjusting for practice characteristics. Reductions in hospitalisation rates were strongest in CKD stages 3b-5, although greatest opportunities for improvement are in CKD stage 3a where practice variation in coding was much wider. There was no difference in death rates according to practice CKD coding (although the relationship was less clear among CKD stages 3b-5). Findings were limited by duration of follow-up with longer-term benefits of CKD coding not yet apparent. Practice behaviours associated with CKD coding including usage of ACEi/ARB therapy and ACR/PCR testing were independent predictors of reduction in hospitalisation rates.

For peer review only - http://bmjopen.bmj.com/site/about/guidelines.xhtml

There are very limited studies looking at the impact of recognition and diagnostic coding of CKD in primary care as many health systems use coded disease as opposed to laboratory records to identify patients. However, recent studies have shown an association between CKD coding and interventions known to reduce CV risk such as prescription of statins and anti-hypertensive agents,[16,17] and CKD coding may play a role in triggering further long-term treatment efforts with potential to reduce patient risks. Our study identified a reduced burden of CV and HF hospitalisations for practices coding more CKD, and a reduced burden of hospitalisations for practices providing more interventions (associated with CKD coding) that are likely to improve CKD outcomes. Numerous studies have also demonstrated disparities in CKD coding efforts with younger patients, those from deprived backgrounds, and ethnic minorities less commonly coded than their counterparts,[7,16] leading to concerns around equity of care.

This study used data captured pre-pandemic, but importance of diagnostic codes on patient files has become even more pertinent during the pandemic. At present in the UK, eligibility for COVID-19 treatment pathways requires presence of diagnostic CKD codes in the healthcare record.[18,19] Patients with CKD stages 4-5 have been deemed suitable for antiviral treatments after a positive COVID test,[18] yet only those with specified CKD codes are detectable under current systems.[19] In the NCKDA database, 9% of CKD patients with most recent eGFR <30 ml/min/1.73m^2 have no CKD diagnostic code on the healthcare record. A subset of eligible patients will therefore not be captured and prioritised for treatment. The percentage of patients missed for treatment could be even higher if patients don't have up-to-date CKD stage codes matching those in data extraction algorithms. More research is needed to investigate the impact of CKD coding on COVID outcomes.

A key strength of this study is the large sample size, including data from 167,208 CKD patients. Data were extracted from GP practices in England with a similar age-sex distribution to the whole population, so findings are likely to be generalisable to the wider population. By studying the association between practice-level CKD coding and patient-level outcomes, we were able to

For peer review only - http://bmjopen.bmj.com/site/about/guidelines.xhtml

eliminate a lot of confounding due to individual patient characteristics that would be present in a conventional study design using patient-level exposure with unmeasured confounders. This was demonstrated by the balanced risk profile in patient characteristics observed across sextiles (which is likely to extend also to unmeasured characteristics). This is a major benefit over original analyses of the NCKDA[12] (**Supplementary Figure 25**) which showed a very strong association between individual patient CKD coding and risk of outcomes (CV events, AKI, death) but with a high risk of confounding due to coding efforts being associated with perceived patient risk, and some potentially important risk factors missing from the database.

A potential weakness is that included practices had volunteered to participate in an audit of care. CKD coding may have been higher in recruited practices than the general population which may have impacted on estimated strengths of associations; benefits of coding in the wider practice population may be larger than estimated. Risk factor evaluation mostly relied on co-morbidity coding and it is not clear whether small differences in risk factor prevalence reflected true morbidity or GP behaviour. Assessment of eligibility of patients for analysis also relied on availability of repeat creatinine tests over time, which may depend on patient risk, and earlier stage CKD cases or more severe cases managed solely in secondary care may be disproportionately missing. Nevertheless, this identified CKD population may stand to benefit most imminently from improvements in primary care, assuming GPs target further coding efforts to patients already identified as at risk and with creatinine test results compatible with CKD. While there was a small signal of more frequent creatinine testing with increasing practice CKD coding, this was only at the extremes, and distribution of CKD severity appeared generally very well balanced across practice coding sextiles. Practice characteristics were analysed differently depending on whether they were likely to confound analyses or lie on the causal pathway, however we could not verify if our assumptions were reasonable, and misspecification could affect reliability of conclusions. For example, practice management of hypertension with ACEi/ARB therapy may plausibly confound analyses (if hypertension management and CKD coding share a common cause, such as practice funding or

For peer review only - http://bmjopen.bmj.com/site/about/guidelines.xhtml

clinical expertise) or lie on the causal pathway (if management of hypertension occurs as a consequence of CKD coding). These data precede the use of SLGT2-inhibitor drug treatment in heart failure and albuminuric kidney disease in UK primary care.

Conclusions

Rates of CV and HF events were lower for patients belonging to practices coding more CKD, supporting the argument that CKD coding in primary care may contribute to improvement in patient outcomes. While the presence of unmeasured confounding cannot be ruled out, this is in agreement with other studies conducted in this setting.[16,17,20] High quality evidence supporting our findings is available from clinical trials and systematic reviews which underline the benefits of use of interventions in early stage CKD, including ACEi/ARB therapy to control hypertension and statin therapy to reduce CV risk.[21-23] This study suggests that reductions in key adverse events for patients with CKD could be made by improvements to GP practice identification and coding of CKD as these are associated with subsequent care efforts that are known to prevent poor outcomes.

For peer review only

FUNDING

This research was funded by the UK MRC, grant reference MR/N013638/1. The funding body had no role in the design of the study, collection, analysis, or interpretation of data and no role in writing of the manuscript.

COMPETING INTERESTS

David C Wheeler has an ongoing consultancy contract with AstraZeneca and has received honoraria, consultancy fees or speaker fees from Amgen, Astellas, Bayer, Boehringer Ingelheim, GlaxoSmithKline, Gilead, Janssen, Napp/Mundipharma, Merck Sharp and Dohme, Tricida, Vifor and Zydus. David Adlam has received research funding from Abbott vascular to support a clinical research fellow; he has also received funding from AstraZeneca inc. for unrelated research and has undertaken consultancy for General Electric inc. to support research funds. Dorothea Nitsch reports grants unrelated to this work from the National Institute for Health Research, Medical Research Council (MRC), the Health Foundation and GlaxoSmithKline.

AUTHOR CONTRIBUTIONS

FC analysed and interpreted the data and was the main author of the manuscript. DN, FC, BC, SH, LK and DP contributed to conception of plans for analysis. All authors read and approved the final manuscript, and contributed insights in the interpretation of results which were incorporated in the manuscript text.

ACKNOWLEDGMENTS

We thank all the patients who have provided their data to the audit analyses, and patient organisations, especially KidneyCare UK and the National Kidney Foundation, and those involved in the set up and conduct of the National Chronic Kidney Disease Audit, which provided a large database of patients with CKD, without which this research would not have been possible. This includes (apart from the listed authors): Matthew Harker, Yvonne Silove, Tasneem Hoosain, Nick

For peer review only

For peer review only - http://bmjopen.bmj.com/site/about/guidelines.xhtml

Wilson, Ronnie Moodley, Chris Gush, Liam Smeeth, Ron Cullen, Fergus Caskey, Andy Syme, Richard Gunn, Paul Wright, Hugh Gallagher, Sion Edwards, Nick Palmer, Anita Sharma, Kate Cheema, Kathleen Mudie.

STROBE REPORTING CHECKLIST

This article adheres to STROBE reporting guidelines for cohort studies in epidemiology. A checklist is provided in **Supplementary Information 3**.

DATA SHARING

The data that support the findings of this study are stored at University College London (UCL) but restrictions apply to the availability of these data, which were used under license for the current study, and so are not publicly available.

ETHICS STATEMENT

The National Chronic Kidney Disease Audit (NCKDA) research database has national ethical approval (REC reference: 17/LO/0049), section 251 approval (CAG reference number: 17CAG0058). NCKDA steering group approval and LSHTM ethics approval (reference: 21689) were granted for this analysis. The NCKDA was implemented in England and Wales in 2015-2016 to review variation in patient care with regards to the identification and management of patients with chronic kidney disease in primary care. It involved extraction of GP practice medical records for a very large number of patients, with linkage to hospital outcomes and mortality databases. The NCKDA database is now being used for further research on which aspects of primary care management improve long-term patient outcomes of patients at risk of or with kidney disease. Research ethics committee approval has been obtained to use pseudonymised clinical data from the NCKDA for research purposes. The pseudonymised database is held at London School of Hygiene and Tropical Medicine (LSHTM) for analysis. There are no personal identifiers (such as names, NHS-numbers or addresses) which allow a direct link between a person's name and their health information held in this clinical dataset; instead

For peer review only

each record has an LSHTM study-id (pseudo-identifier) attached. A study-id is a unique number added to an individual's record for use in the study. The information on NHS numbers and corresponding study-id (pseudo-identifiers) are held separately at University College London in a super-secure server.

For peer review only

For peer review only - http://bmjopen.bmj.com/site/about/guidelines.xhtml

REFERENCES

1. GBD Chronic Kidney Disease Collaboration. Global, regional, and national burden of chronic kidney disease, 1990-2017: a systematic analysis for the Global Burden of Disease Study 2017. *Lancet* 2020;395(10225):709-33.
2. Jager KJ, Fraser SDS. The ascending rank of chronic kidney disease in the global burden of disease study. *Nephrology Dialysis Transplantation* 2017;32(suppl_2):ii121-ii28.
3. Schoolwerth AC, Engelgau MM, Hostetter TH, et al. Chronic kidney disease: a public health problem that needs a public health action plan. *Preventing Chronic Disease* 2006;3(2):A57.
4. Kidney Disease: Improving Global Outcomes (KDIGO) CKD Work Group. KDIGO 2012 Clinical Practice Guideline for the Evaluation and Management of Chronic Kidney Disease. *Kidney inter.*, Suppl 2013; 3: 1–150.
5. Kerr M, Bray B, Medcalf J, et al. Estimating the financial cost of chronic kidney disease to the NHS in England. *Nephrol Dial Transplant* 2012;27 Suppl 3:iii73-80.
6. UK Renal Registry (2020) UK Renal Registry 22nd Annual Report – data to 31/12/2018, Bristol, UK. 2020. https://ukkidney.org/audit-research/annual-report/22nd-annual-report-data-31122018. Accessed 27 Jan 2022.
7. Kim LG, Cleary F, Wheeler DC, et al. How do primary care doctors in England and Wales code and manage people with chronic kidney disease? Results from the National Chronic Kidney Disease Audit. *Nephrology Dialysis Transplantation* 2017;33(8):1373-79.
8. Nitsch D, Caplin B, Hull S, et al on behalf of the National CKD Audit and Quality Improvement Programme in Primary Care. First National CKD Audit Report. 2017. https://www.lshtm.ac.uk/files/ckd_audit_report.pdf. Accessed 28 Jul 2021.
9. NHS Digital. SNOMED CT. https://digital.nhs.uk/services/terminology-and-classifications/snomed-ct (2021). Accessed 27 Jan 2022.

For peer review only

For peer review only - http://bmjopen.bmj.com/site/about/guidelines.xhtml

10. National Institute for Health and Care Excellence. Chronic Kidney Disease in Adults: Assessment and Management (NICE Guideline CG182). 2014. https://www.nice.org.uk/guidance/cg182. Accessed 28 Jul 2021.
11. National Institute for Health and Care Excellence. NICE Quality and Outcomes Framework indicator. https://www.nice.org.uk/standards-and-indicators/qofindicators. Accessed 27 Jan 2022.
12. Mudie K, Cleary F, Caplin B, et al on behalf of the National CKD Audit and Quality Improvement Programme in Primary Care. Second National CKD Audit Report. 2017. https://www.lshtm.ac.uk/media/9951. Accessed 28 Jul 2021.
13. Capurro D, Coghlan S, Pires DEV. Preventing Digital Overdiagnosis. *JAMA* 2022;327(6):525-6.
14. London School of Hygiene and Tropical Medicine. National CKD Audit Steering Committee. https://www.lshtm.ac.uk/research/centres-projects-groups/ckdaudit#steering-committee. Accessed 27 Jan 2022.
15. NHS Digital. Read Codes. https://digital.nhs.uk/services/terminology-and-classifications/read-codes (2020). Accessed 27 Jan 2022.
16. Molokhia M, Okoli GN, Redmond P, et al. Uncoded chronic kidney disease in primary care: a cross-sectional study of inequalities and cardiovascular disease risk management. *British Journal of General Practice* 2020;70(700):e785-e92.
17. Hull SA, Rajabzadeh V, Thomas N, et al. Improving coding and primary care management for patients with chronic kidney disease: an observational controlled study in East London. *British Journal of General Practice* 2019;69(684):e454-e61.
18. NHS Digital. Treatments for coronavirus (COVID-19). https://digital.nhs.uk/coronavirus/treatments (2022). Accessed 18 Feb 2022.
19. NHS Digital. Population Health COVID-19 Treatment Methodology: Coding classifications used. https://digital.nhs.uk/coronavirus/treatments/methodology/coding-classifications-used (2022). Accessed 18 Feb 2022.

For peer review only - http://bmjopen.bmj.com/site/about/guidelines.xhtml

20. Olaitan A, Ashman N, Homer K, et al. Predictors of late presentation to renal dialysis: a cohort study of linked primary and secondary care records in East London. *BMJ Open* 2019;9(6):e028431.

21. Xie X, Liu Y, Perkovic V, et al. Renin-Angiotensin System Inhibitors and Kidney and Cardiovascular Outcomes in Patients With CKD: A Bayesian Network Meta-analysis of Randomized Clinical Trials. *Am J Kidney Dis* 2016;67(5):728-41.

22. Herrington WG, Emberson J, Mihaylova B, et al. Impact of renal function on the effects of LDL cholesterol lowering with statin-based regimens: a meta-analysis of individual participant data from 28 randomised trials. *Lancet Diabetes Endocrinol* 2016;4(10):829-39.

23. Wright JT Jr, Williamson JD, Whelton PK, et al. A Randomized Trial of Intensive versus Standard Blood-Pressure Control. *New England Journal of Medicine* 2015;373(22):2103-16.

For peer review only

For peer review only - http://bmjopen.bmj.com/site/about/guidelines.xhtml

FIGURE LEGENGS

Figure 1. Flow chart of selection of study population (confirmed CKD in last 2 years)

Figure 2. HR curves for all outcomes according to practice percent coded CKD.
Analysis includes all patients with confirmed CKD. Primary exposure is (continuous) practice percent coded CKD, with median practice coding (74% coded) as the reference group. Model 1 adjusts for practice risk profile characteristics: mean age, percent male, median rank of index of multiple deprivation, diabetes prevalence, hypertension prevalence, CVD prevalence. Model 2 adjusts for model 1 variables and additionally for practice characteristics of CKD population and testing behaviours: percent of CKD cases at stages 3b-5, percent of CKD patients admitted for COPD in last 3 years, percent of CKD patients admitted for cancer in last 3 years, percent GFR test in last year in diabetes, percent GFR test in last year in CKD, percent of adult population with CKD. Labels S1 to S6 descriptively indicate sextiles of practice percent coded CKD, with each sextile representing one sixth of all practices.

For peer review only

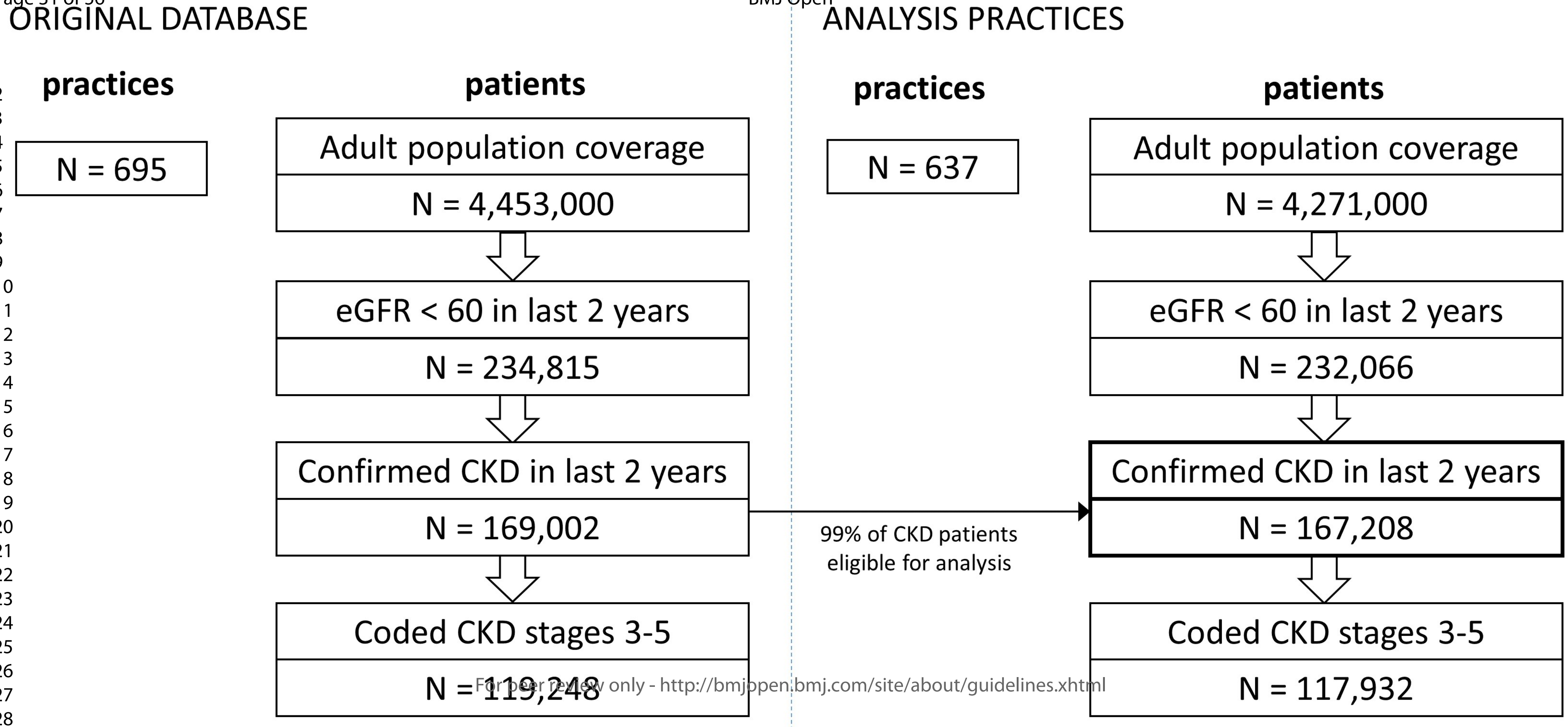

For peer review only - http://bmjopen.bmj.com/site/about/guidelines.xhtml

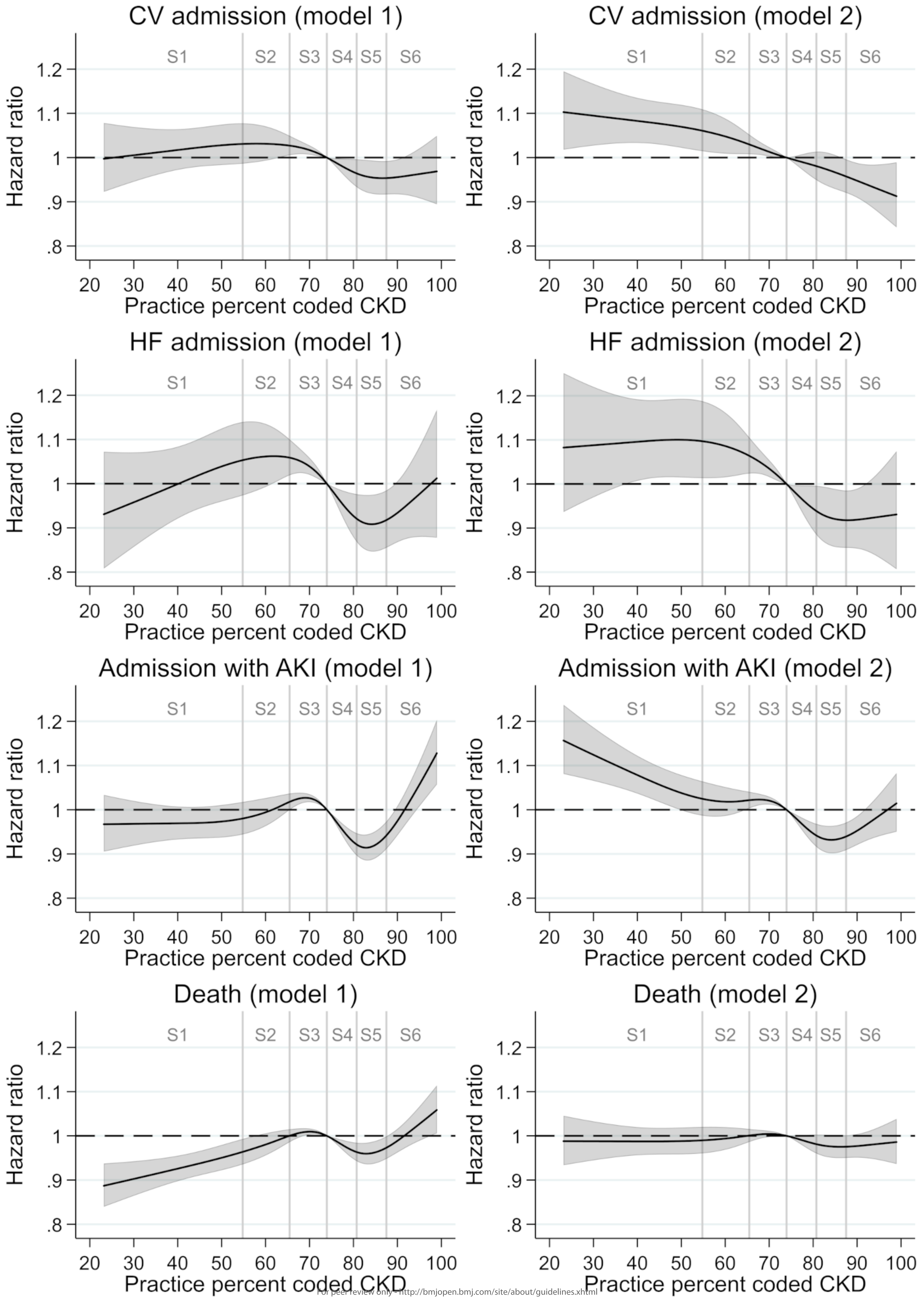
CV admission (model 1)
CV admission (model 2)
HF admission (model 1)
HF admission (model 2)
Admission with AKI (model 1)
Admission with AKI (model 2)
Death (model 1)
Death (model 2)
S1
S2
S3
S4
S5
S6
Hazard ratio
1.2
1.1
1
.9
.8
20
30
40
50
60
70
80
90
100
Practice percent coded CKD

SUPPLEMENTARY MATERIALS

Supplementary Table 1. Outcome definitions

Outcome	Definition	Details	ICD-10 codes
AKI	AKI at admission	Any diagnosis (HES) of AKI recorded in the first episode of care	N17
HF	Admission for HF	A primary diagnosis (HES) of HF recorded in the first episode of care	I50, I11.0, I13, I97.1
CV event	Admission for CV event	A primary diagnosis (HES) of HF, CHD, stroke/TIA, PAD or AAA recorded in the first episode of care	HF (I50, I11.0, I13, I97.1)<br>CHD (I20-I25, I51.6)<br>Stroke/TIA (G45-G46)<br>PAD (I79.0, I79.2, I73.8, I73.9, I74.3, I74.4, I74.5, I70.2)<br>AAA (I71, I74.0)<br>Cerebrovascular disease (I60-I69)
All-cause mortality	All-cause mortality	Any death (ONS)	N/A

Supplementary Table 2. Percentage of practices and patients eligible for analysis after application of sample size eligibility criteria (minimum of 50 CKD cases) and comparison of coding rates in the original database and analysis database, in the CKD population and in subgroups

Population	Total practices	Total patients	Total patients coded	Eligible practices	Eligible patients	Eligible patients coded
All CKD	695	169,002	119,248 (70.6%)	637 (91.7%)	167,208 (98.9%)	117,932 (70.5%)
CKD stage 3a	695	106,981	66,514 (62.2%)	580 (83.5%)	103,615 (96.9%)	64,398 (62.2%)
CKD stages 3b-5	695	62,021	52,734 (85.0%)	477 (68.6%)	56,122 (90.5%)	47,700 (84.5%)
Diabetes	695	42,063	32,099 (76.3%)	362 (52.1%)	33,065 (78.6%)	25,273 (76.4%)
No diabetes	695	126,939	87,149 (68.7%)	605 (87.1%)	124,364 (98.0%)	85,324 (68.6%)

For peer review only - http://bmjopen.bmj.com/site/about/guidelines.xhtml

Supplementary Information 1. Methods for estimating the percentage of CV and HF hospitalisation events that are preventable among CKD patients in practices coding 55% of CKD cases if practice coding improved to 88% (attributable fraction for first events)

In order to estimate the percentage of first hospitalisation events that could be prevented over a period of 3.8 years (median follow-up duration) among patients in practices coding 55% of CKD cases (13th practice coding percentile, lower boundary of sextile 2), if such practices instead coded 88% of CKD cases (83rd practice coding percentile, upper boundary of sextile 5), we adopted the Austin method in [1], which we adapted for use in STATA statistical software. We make an assumption that after adjustment for model covariates, the difference in expected event rates between practice coding groups estimated using the methods listed below is caused by practice coding performance. We followed the following steps to estimate the percentage of first events attributable to lower practice coding (55%), when compared to higher practice coding (88%), for both CV and HF hospitalisations:

1. Fit the fully adjusted Cox regression model for time to first event with 5-knot spline for practice percent coded CKD and other practice covariates (model 2), first centring all continuous covariates to generate a sensible baseline group
2. Estimate the baseline survival function [2] using: "predict s, basesurv", sort data by analysis time and extract the baseline survival probability at t = 3.8 years (median follow-up)
3. Predict the estimated survival probability at t = 3.8 years for every patient in database assuming 54.8% practice percent coded, based on true values of all covariates except with practice percent coded forced to 54.8%, as follows:
   a. Recode data to set value of practice coding variable to 54.8% coded in all patients. (Spline variable values recoded accordingly.)
   b. Use equation $\hat{S}_i(t) = \hat{S}_0(t)^{\exp(\boldsymbol{x}_i\hat{\boldsymbol{\beta}})}$ [3] to estimate individual survival probabilities $\hat{S}_i(t)$ at time t = 3.8 years, where $\hat{S}_0(t)$ is the estimated baseline survival at time t and $\boldsymbol{x}_i\hat{\boldsymbol{\beta}}$ is the individual prediction of the linear predictor evaluated at individual true covariate values $\boldsymbol{x}_i$ (but specified practice percent coded of 54.8%) based on coefficient estimates $\hat{\boldsymbol{\beta}}$
4. Repeat step 3 for practice percent coded forced to 87.5% coded, to obtain individual predicted survival probabilities at t = 3.8 years assuming 87.5% practice percent coded
5. Estimate the expected number of first events occurring over 3.8 years if all practices coded at 54.8% (with other practice characteristics unchanged) by taking the sum of individual probabilities of an event (1 - $\hat{S}_i(t = 3.8)$, using results from (3b)). Repeat for assumption of all practices coding at 87.5% by taking the sum of individual probabilities of an event (1 - $\hat{S}_i(t = 3.8)$, from (4b)).
6. Using results from step 5, compute the percentage of events preventable among individuals in practices coding 54.8% of CKD cases if practices instead coded 87.5% of CKD cases (attributable fraction) as:

$$\%\ events\ preventable\ in\ practices\ coding\ 54.8\%\ of\ CKD\ cases$$

$$= \frac{expected\ events\ assuming\ 54.8\%\ coding - expected\ events\ assuming\ 87.5\%\ coding}{expected\ events\ assuming\ 54.8\%\ coding} \times 100\%$$

For peer review only - http://bmjopen.bmj.com/site/about/guidelines.xhtml

References:

1. Zhang Z, Ambrogi F, Bokov AF, et al. Estimate risk difference and number needed to treat in survival analysis. *Ann Transl Med* 2018;6(7):120.

2. StataCorp. STATA Survival Analysis Reference Manual Release 17. 2021. StataCorp LLC. https://www.stata.com/manuals/st.pdf. Accessed 17 Feb 2022.

3. Rodriguez, G. Approaches to Survival Modelling. In: Lecture Notes on Generalized Linear Models. Princeton University. 2007. https://data.princeton.edu/wws509/notes/c7.pdf. Accessed 17 Feb 2022

For peer review only

Supplementary Figure 1. Distribution of practice CKD coding performance in the CKD analysis population and in subgroups, with red lines depicting sextile boundaries

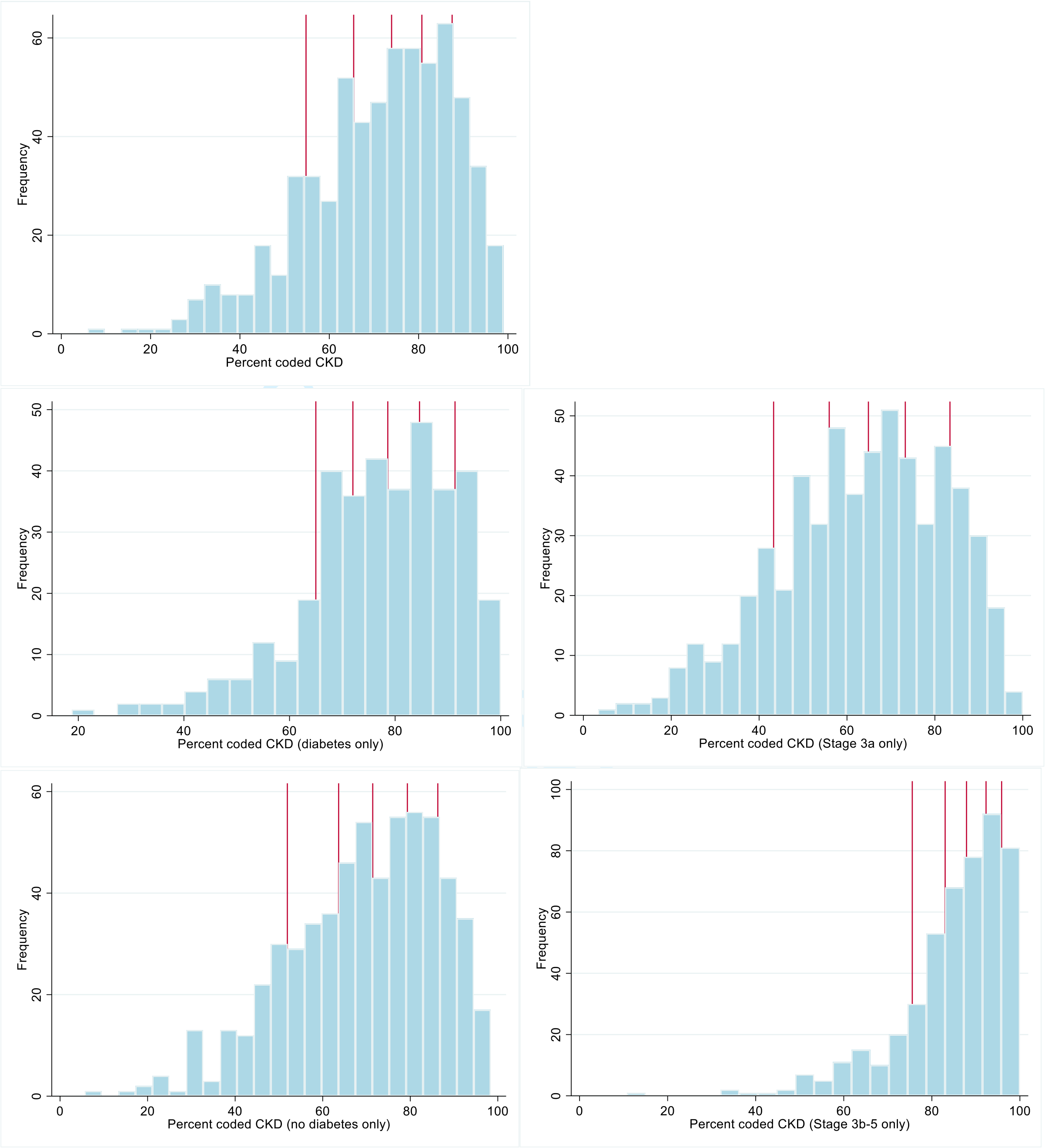

For peer review only - http://bmjopen.bmj.com/site/about/guidelines.xhtml

Supplementary Figure 2. Event rates per 100 patient years and 95% confidence intervals, by practice coding sextile in **all CKD** patients (including **recurring events**)

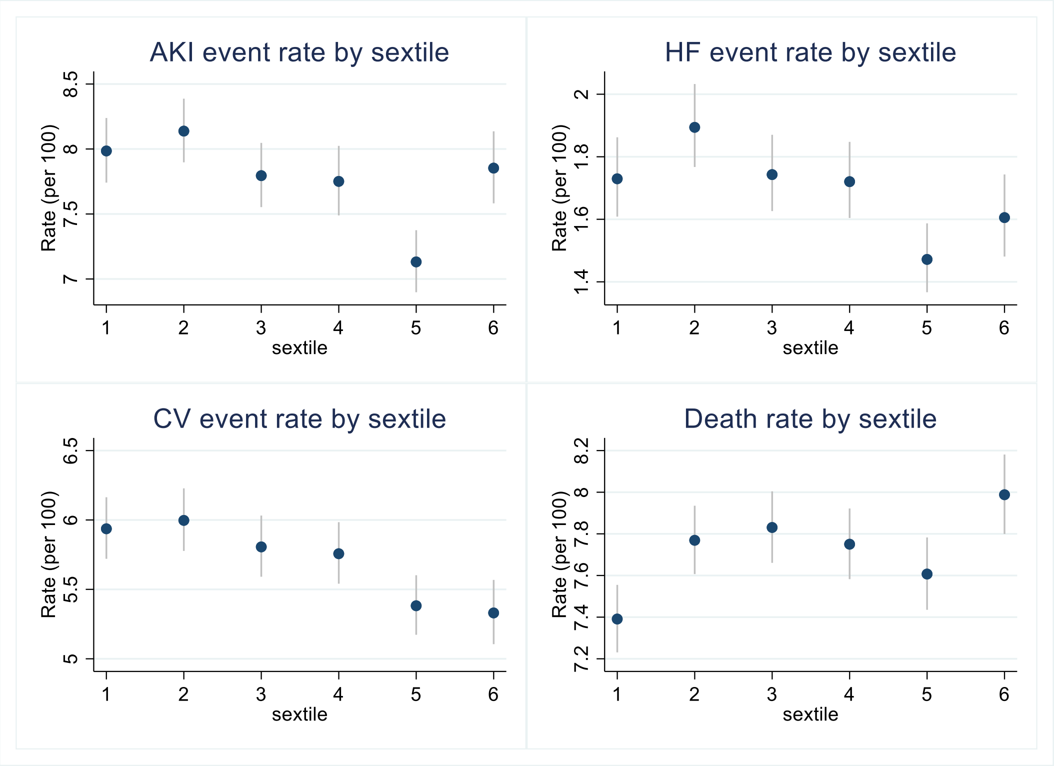

Supplementary Figure 3. Event rates per 100 patient years and 95% confidence intervals, by practice coding sextile in **all CKD** patients (**first events** only)

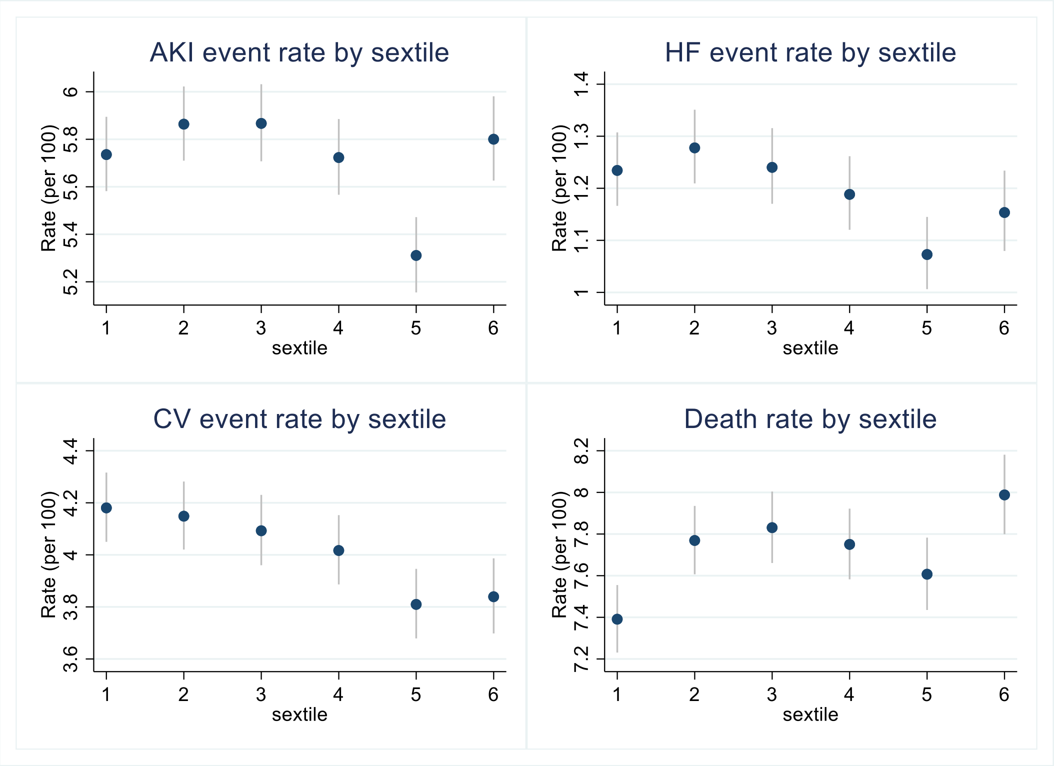

For peer review only - http://bmjopen.bmj.com/site/about/guidelines.xhtml

Supplementary Table 3. Event rates (including recurring events) per 100 patient years and 95% confidence intervals, by practice coding sextile

Practice coding sextile (percent coded CKD)	S1 (<54.8%)	S2 (54.8% - 65.5%)	S3 (65.5% - 73.9%)	S4 (73.9% - 80.7%)	S5 (80.7% - 87.5%)	S6 (≥87.5%)
CV event	5.94 (5.72, 6.16)	6.00 (5.78, 6.23)	5.81 (5.59, 6.03)	5.76 (5.54, 5.98)	5.38 (5.17, 5.60)	5.33 (5.11, 5.57)
HF	1.73 (1.61, 1.86)	1.89 (1.77, 2.03)	1.74 (1.63, 1.87)	1.72 (1.60, 1.85)	1.47 (1.37, 1.59)	1.61 (1.48, 1.74)
AKI	7.99 (7.74, 8.24)	8.14 (7.90, 8.39)	7.80 (7.55, 8.05)	7.75 (7.49, 8.02)	7.13 (6.90, 7.38)	7.85 (7.58, 8.14)
All-cause mortality	7.39 (7.23, 7.55)	7.77 (7.61, 7.93)	7.83 (7.66, 8.00)	7.75 (7.58, 7.92)	7.61 (7.43, 7.78)	7.99 (7.80, 8.18)

Supplementary Information 2. Methods for sequentially adjusted Cox regression analyses shown in supplementary analyses

Main Cox regression analyses, models 1 and 2, adjusted for practice characteristics are described in the main methods (repeated here for completeness) with results for all studied outcomes shown in the main results. Supplementary figures 4-23 show additional sequential adjustments, demonstrating the role of confounding as different variables were incorporated in analyses, as follows:

- Crude analysis: unadjusted
- Model 1: adjusted for ***practice characteristics reflecting practice risk profile*** (mean age, percent male, median rank of IMD, diabetes prevalence, hypertension prevalence, CVD prevalence)
- Model 1.5: adjusted for ***practice characteristics reflecting practice risk profile*** (model 1 variables), as well as ***practice characteristics of the detected CKD population*** (percent of CKD cases stages 3b-5 ["CKD severity"]), percent admitted for COPD in last 3 years, percent admitted for cancer in last 3 years)
- Model 2: adjusted for ***practice characteristics relating to overall practice risk profile*** and ***of the detected CKD population*** (model 1.5 variables), as well as ***testing biases*** which may result in confounding due to different vintages (i.e. duration of underlying disease) (percent GFR test in last year in diabetes, percent GFR test in last year in CKD, percent of adult population with detected CKD)

As in main analyses, adjusted hazard ratios (HRs) for outcomes with 95% confidence intervals were plotted across the spectrum of practice CKD coding, compared to average practice CKD coding.

For peer review only - http://bmjopen.bmj.com/site/about/guidelines.xhtml

Supplementary Figure 4. Hazard ratio splines for time to first **CV event**, compared to median (73.9%) practice percent coded (sequential adjustments)

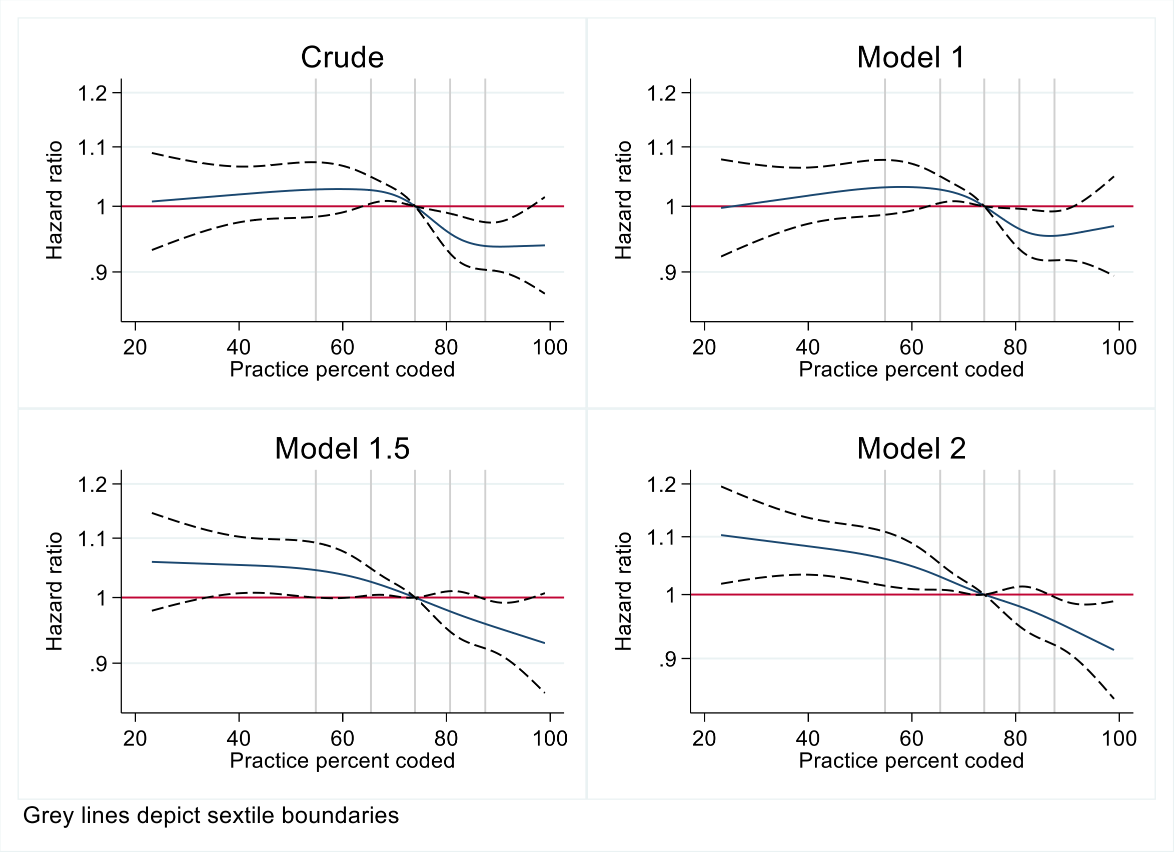

For peer review only - http://bmjopen.bmj.com/site/about/guidelines.xhtml

Supplementary Figure 5. Hazard ratio splines for time to first **CV event**, compared to median (64.9%) practice percent coded, in **CKD stage 3a only** (sequential adjustments)

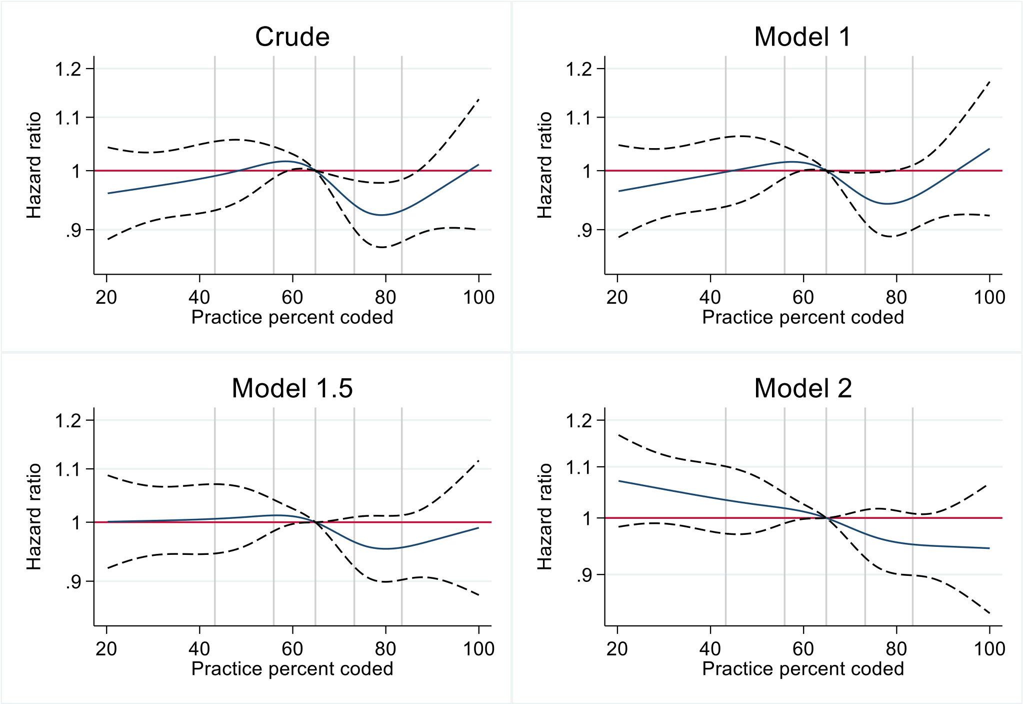

Grey lines depict sextile boundaries

Supplementary Figure 6. Hazard ratio splines for time to first **CV event**, compared to median (87.9%) practice percent coded, in **CKD stages 3b-5 only** (sequential adjustments)

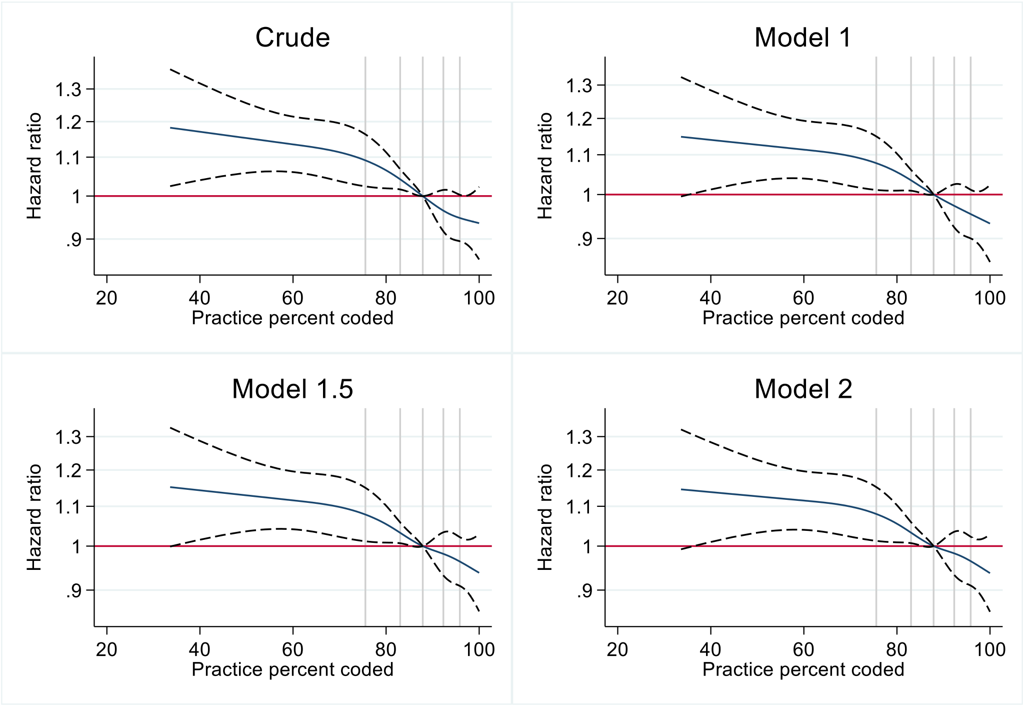

Grey lines depict sextile boundaries

For peer review only - http://bmjopen.bmj.com/site/about/guidelines.xhtml

Supplementary Figure 7. Hazard ratio splines for time to first **CV event**, compared to median (78.6%) practice percent coded, in **diabetes only** (sequential adjustments)

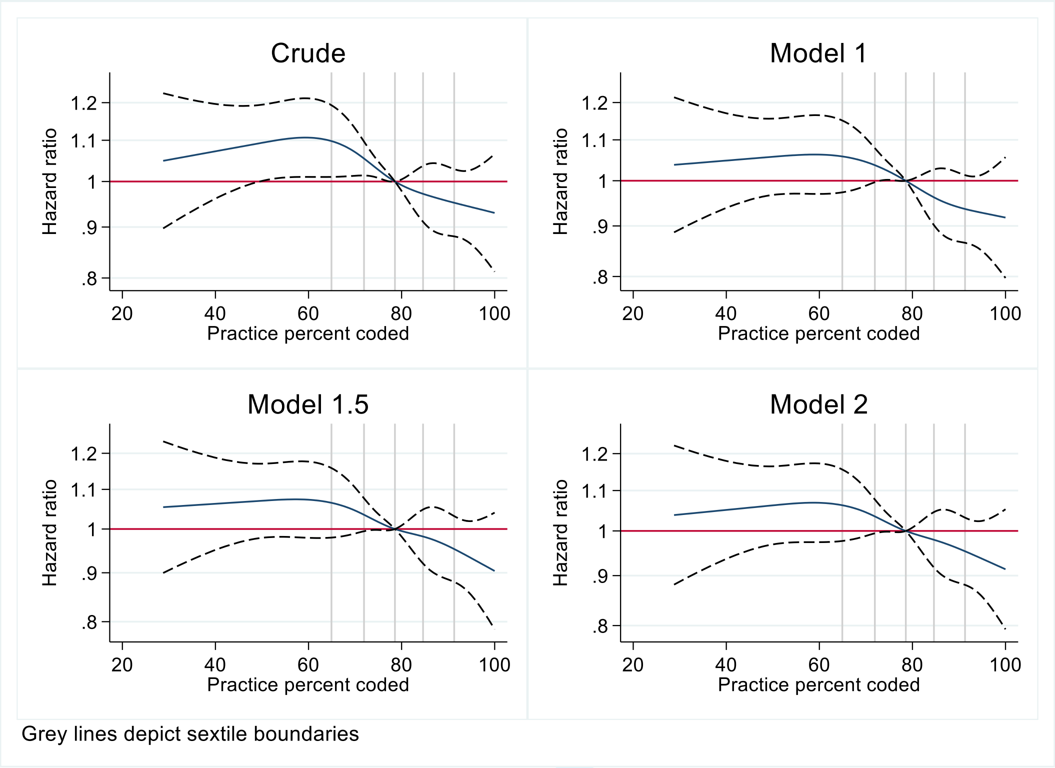

Supplementary Figure 8. Hazard ratio splines for time to first **CV event**, compared to median (71.4%) practice percent coded, in **no diabetes only** (sequential adjustments)

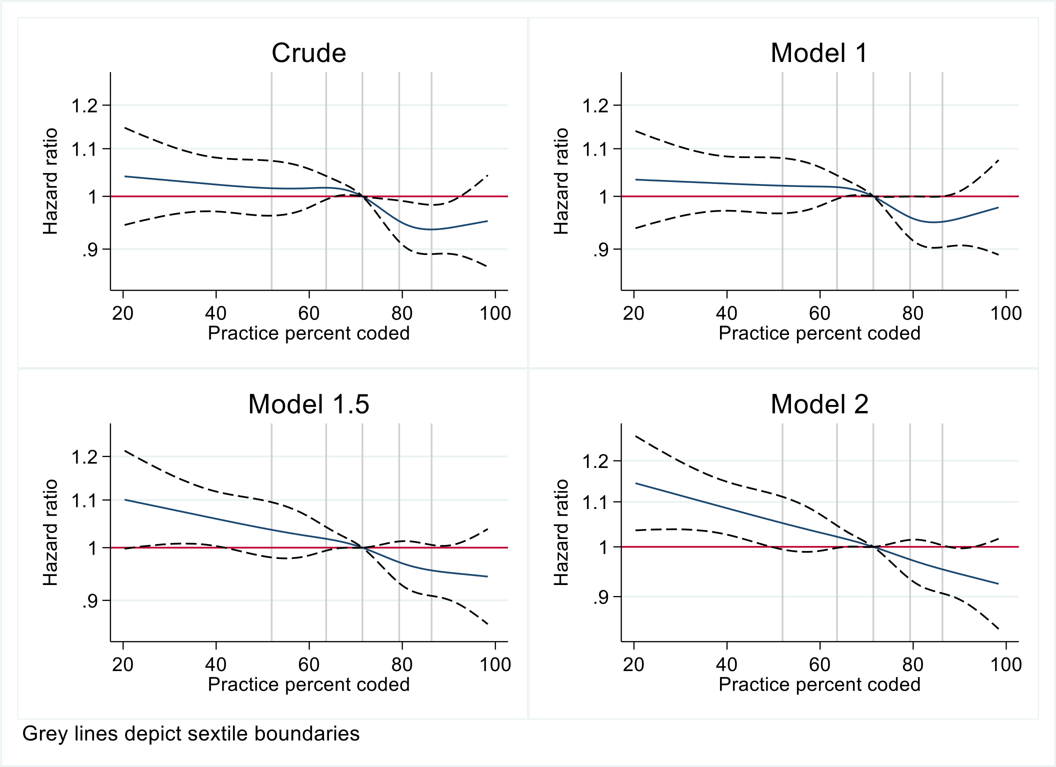

For peer review only - http://bmjopen.bmj.com/site/about/guidelines.xhtml

Supplementary Figure 9. Hazard ratio splines for time to first **HF** event, compared to median (73.9%) practice percent coded (sequential adjustments)

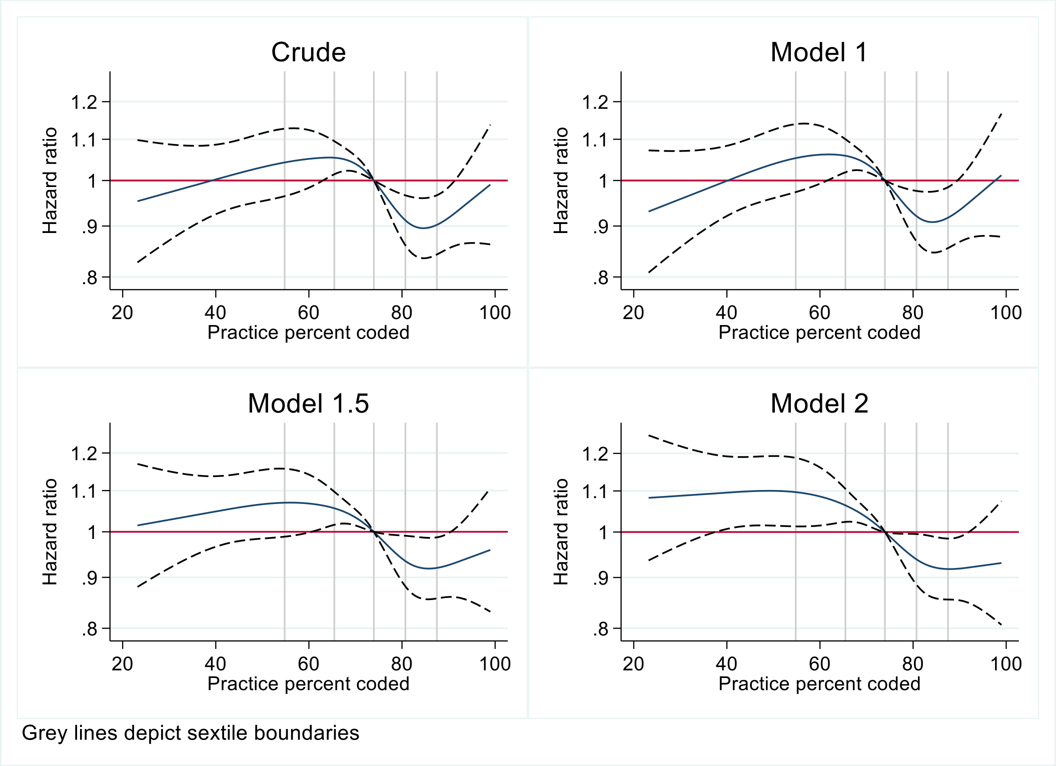

For peer review only - http://bmjopen.bmj.com/site/about/guidelines.xhtml

Supplementary Figure 10. Hazard ratio splines for time to first **HF** event, compared to median (64.9%) practice percent coded, in **CKD stage 3a only** (sequential adjustments)

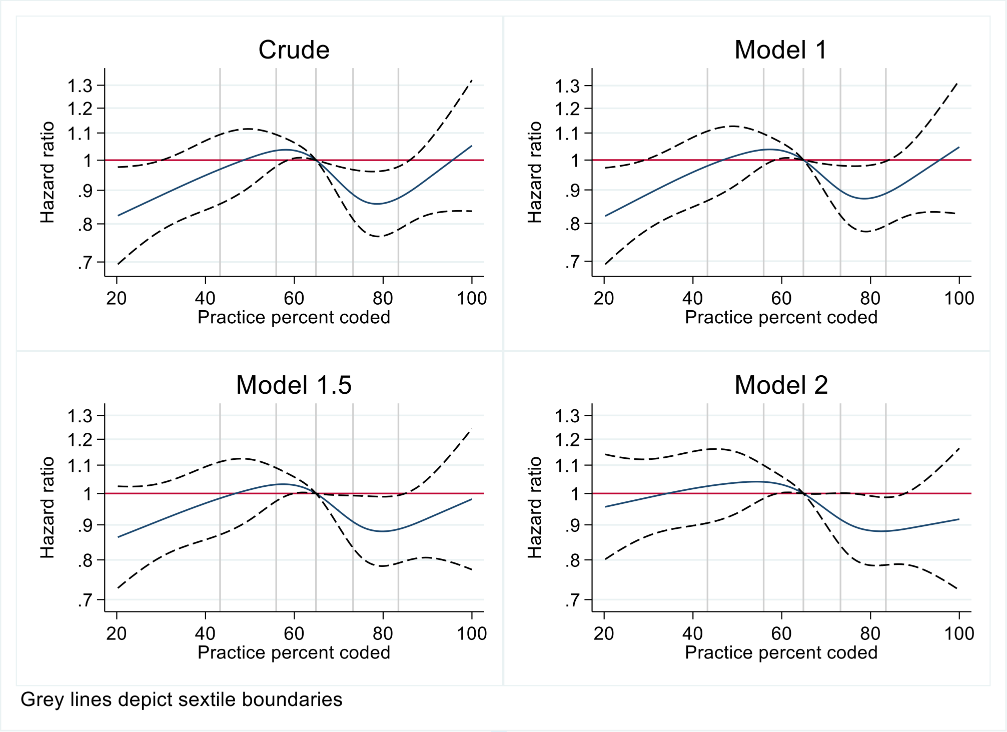

Supplementary Figure 11. Hazard ratio splines for time to first **HF** event, compared to median (87.9%) practice percent coded, in **CKD stages 3b-5** only (sequential adjustments)

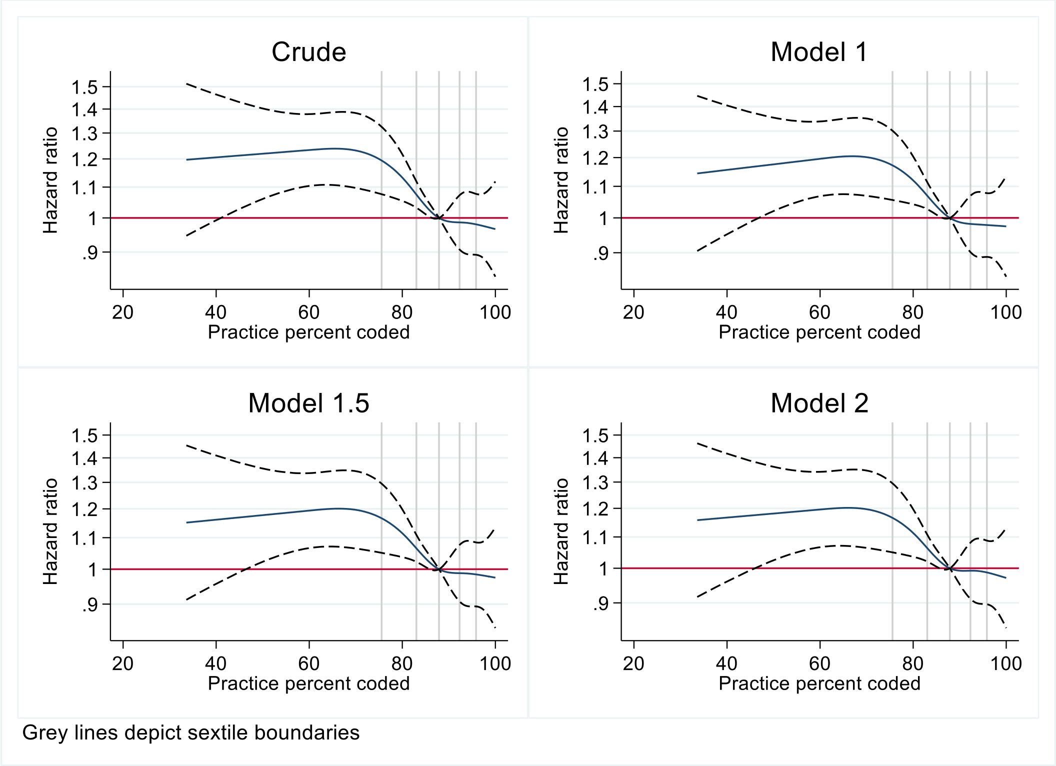

For peer review only - http://bmjopen.bmj.com/site/about/guidelines.xhtml

Supplementary Figure 12. Hazard ratio splines for time to first **HF** event, compared to median (78.6%) practice percent coded, in **diabetes only** (sequential adjustments)

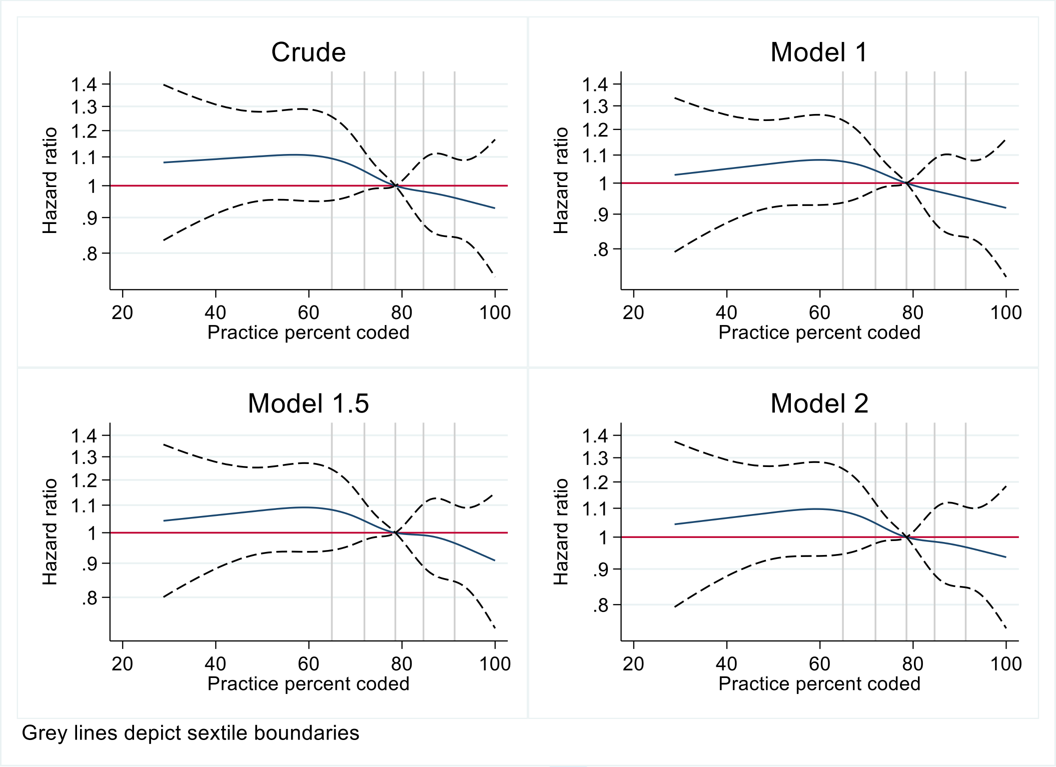

Supplementary Figure 13. Hazard ratio splines for time to first **HF** event, compared to median (71.4%) practice percent coded, in **no diabetes only** (sequential adjustments)

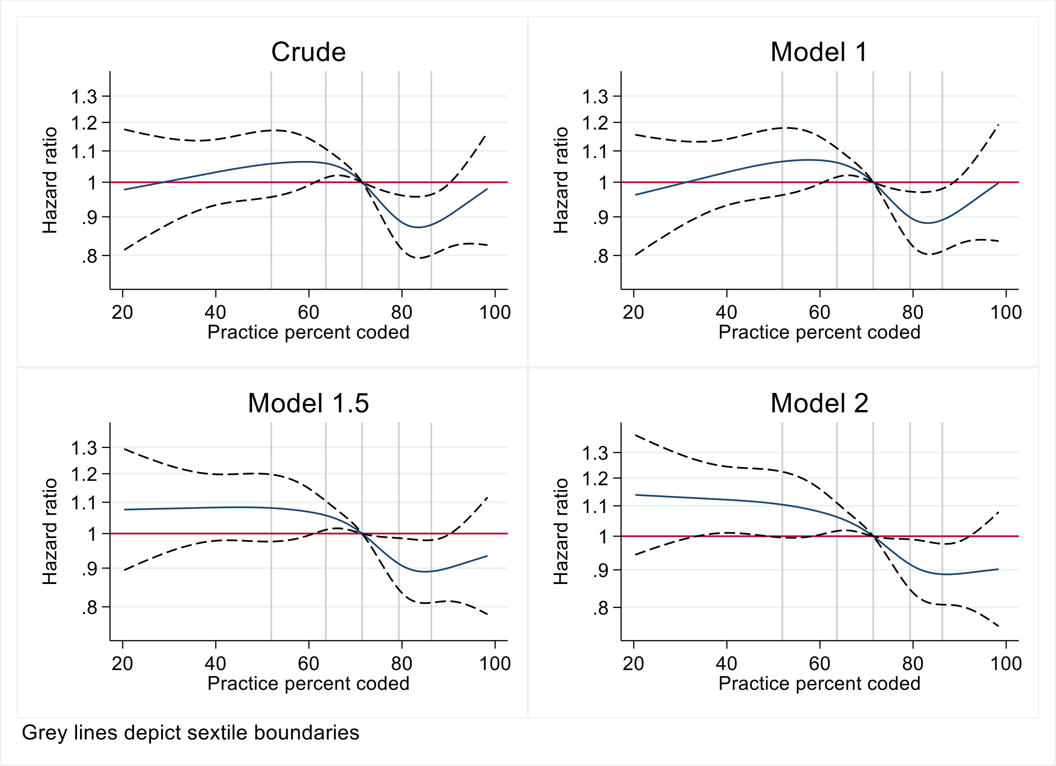

For peer review only - http://bmjopen.bmj.com/site/about/guidelines.xhtml

Supplementary Figure 14. Hazard ratio splines for time to first **AKI** event, compared to median (73.9%) practice percent coded (sequential adjustments)

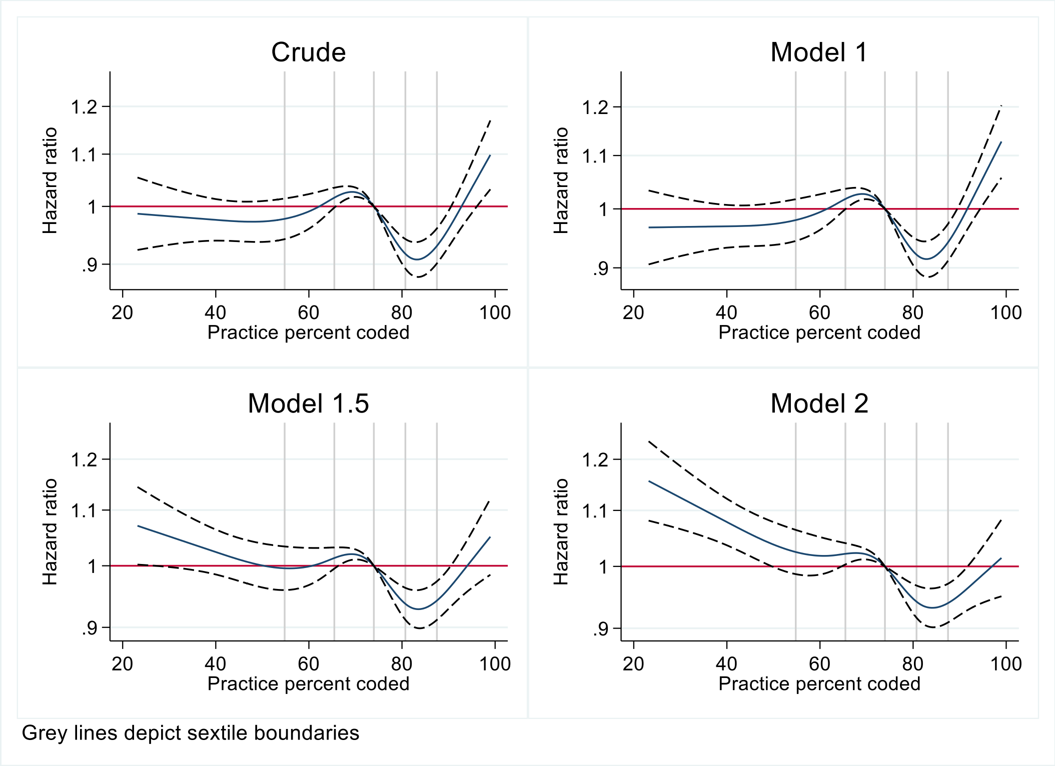

For peer review only

Supplementary Figure 15 Hazard ratio splines for time to first **AKI** event, compared to median (64.9%) practice percent coded, in **CKD stage 3a only** (sequential adjustments)

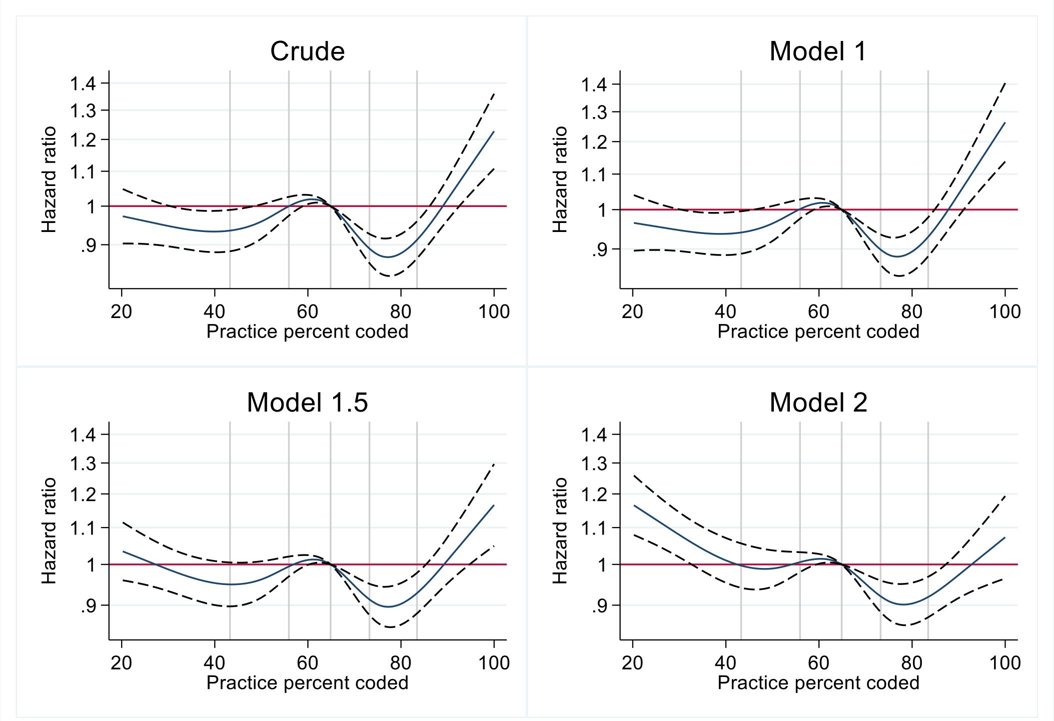

Grey lines depict sextile boundaries

Supplementary Figure 16. Hazard ratio splines for time to first **AKI** event, compared to median (87.9%) practice percent coded, in **CKD stages 3b-5 only** (sequential adjustments)

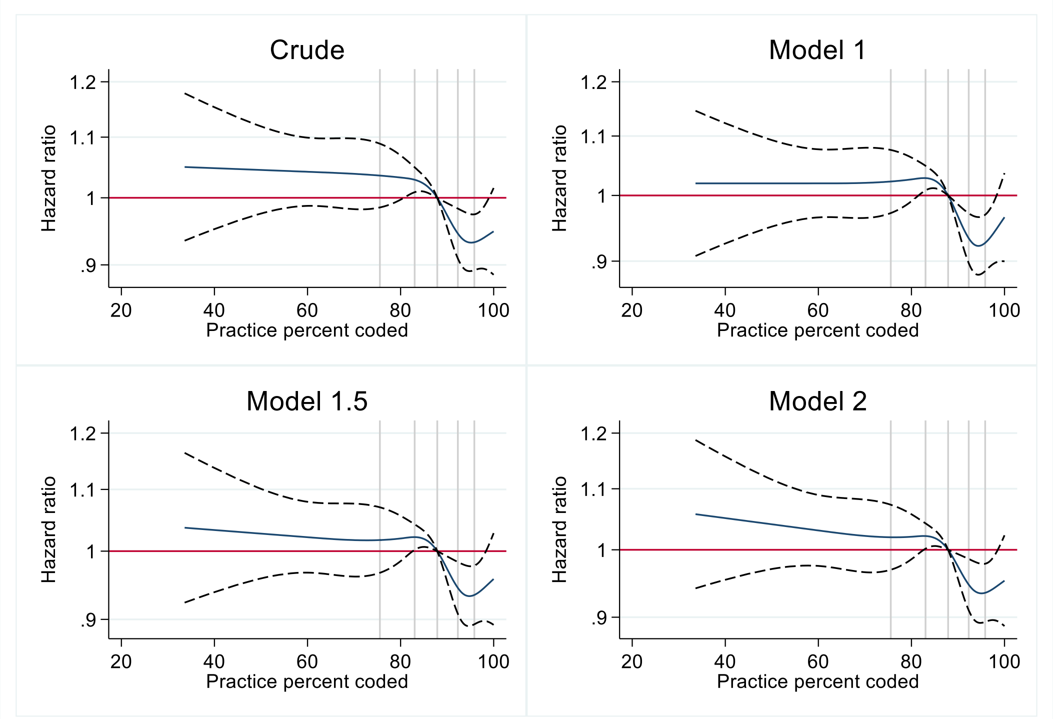

Grey lines depict sextile boundaries

Supplementary Figure 17. Hazard ratio splines for time to first **AKI** event, compared to median (78.6%) practice percent coded, in **diabetes only** (sequential adjustments)

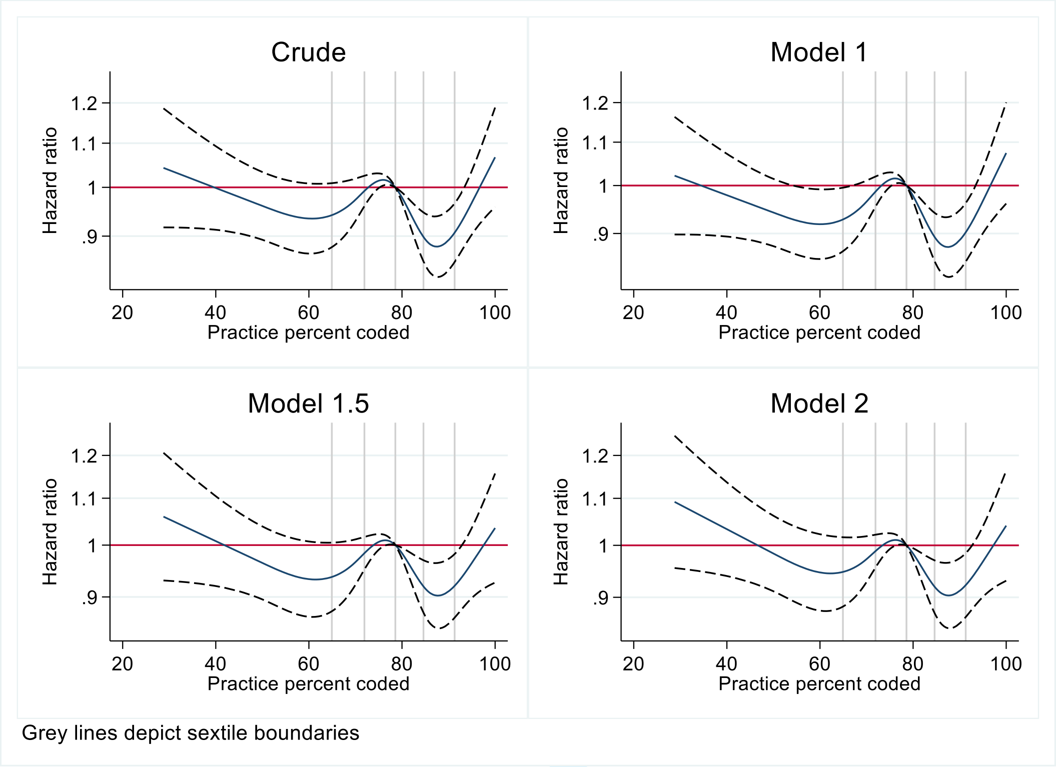

Supplementary Figure 18. Hazard ratio splines for time to first **AKI** event, compared to median (71.4%) practice percent coded, in **no diabetes only** (sequential adjustments)

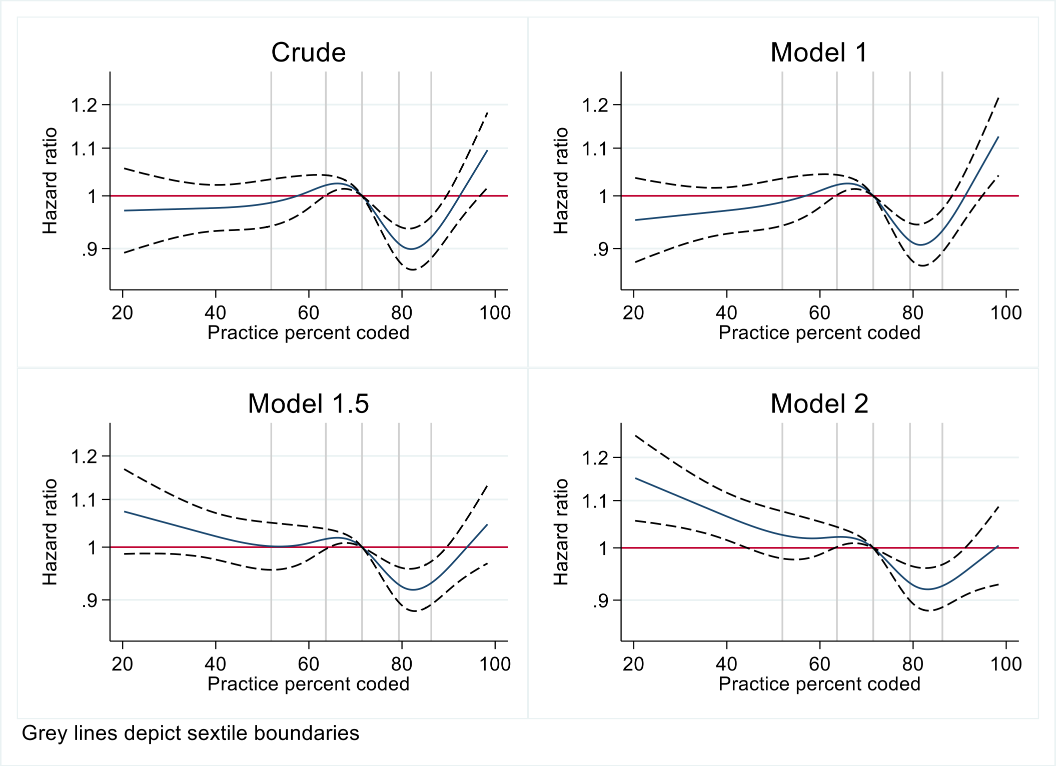

Supplementary Figure 19. Hazard ratio splines for time to **death**, compared to median (73.9%) practice percent coded (sequential adjustments)

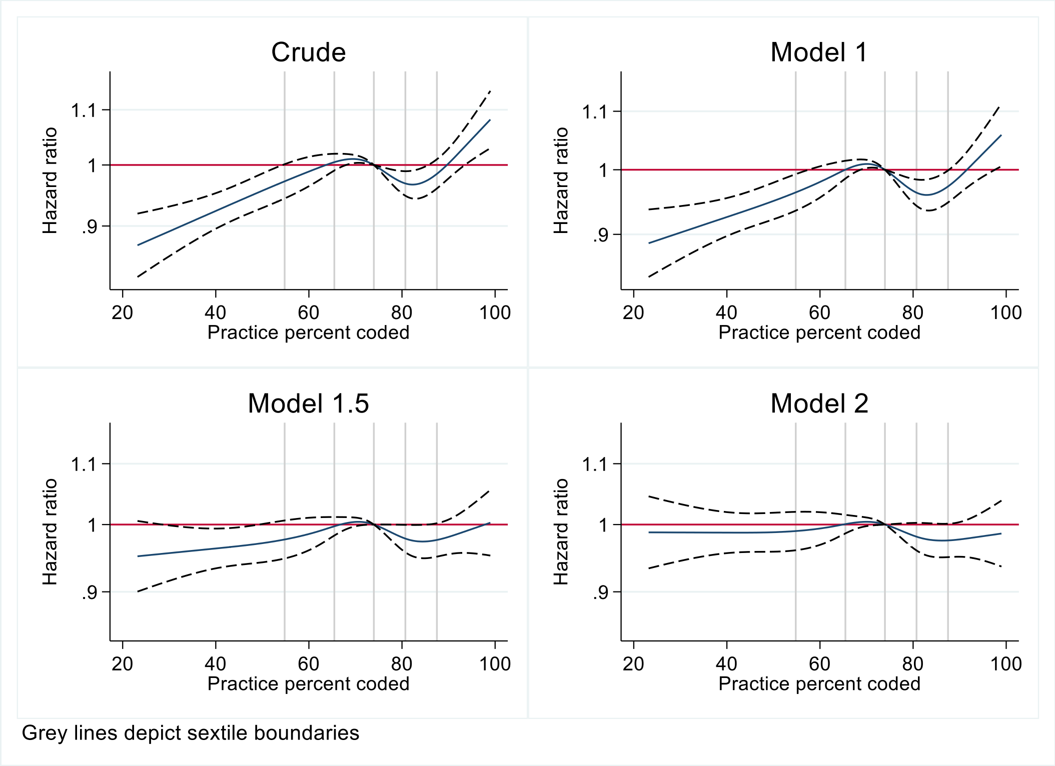

review only

For peer review only - http://bmjopen.bmj.com/site/about/guidelines.xhtml

Supplementary Figure 20. Hazard ratio splines for time to **death**, compared to median (64.9%) practice percent coded, in **CKD stage 3a only** (sequential adjustments)

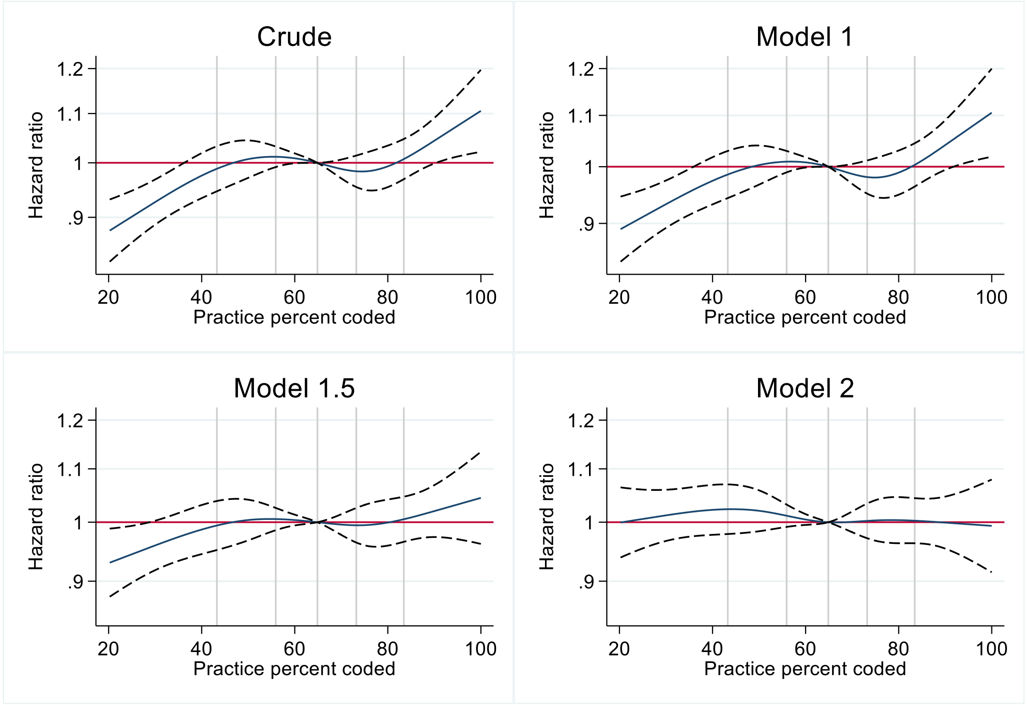

Grey lines depict sextile boundaries

Supplementary Figure 21. Hazard ratio splines for time to **death**, compared to median (87.9%) practice percent coded, in **CKD stages 3b-5 only** (sequential adjustments)

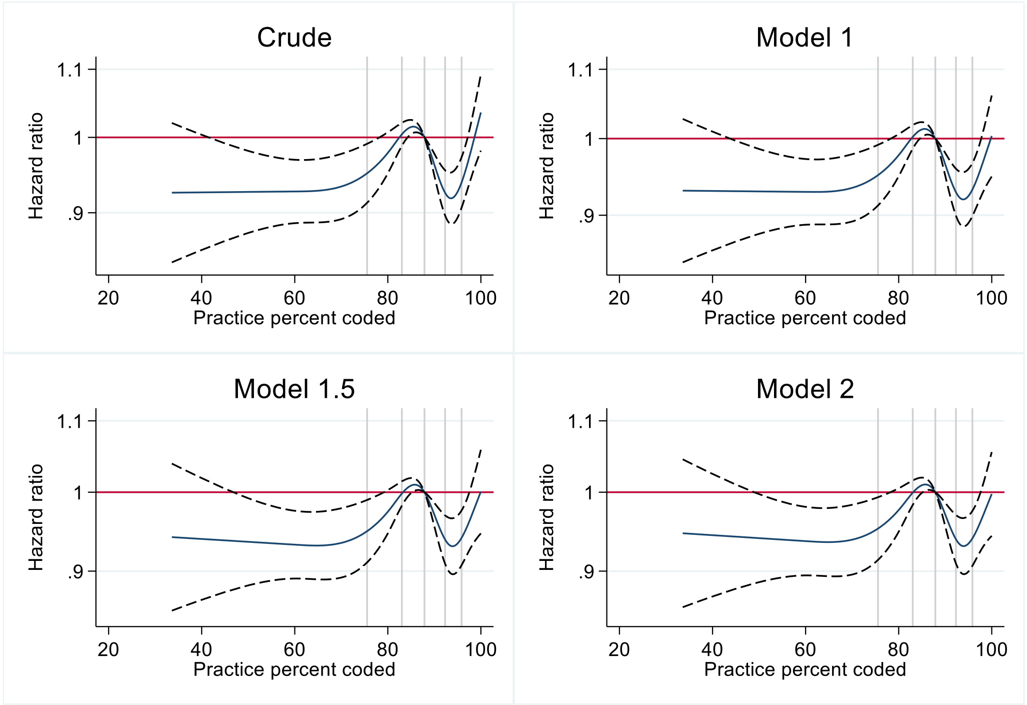

Grey lines depict sextile boundaries

Supplementary Figure 22. Hazard ratio splines for time to **death**, compared to median (78.6%) practice percent coded, in **diabetes only** (sequential adjustments)

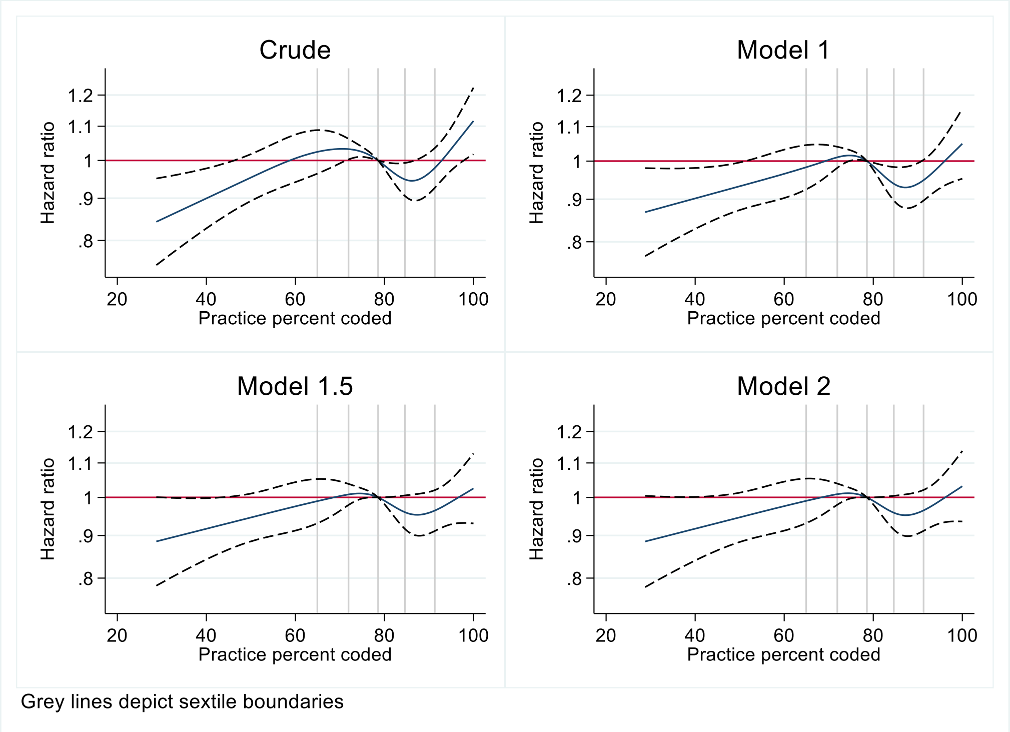

Supplementary Figure 23. Hazard ratio splines for time to **death**, compared to median (71.4%) practice percent coded, in **no diabetes** only (sequential adjustments)

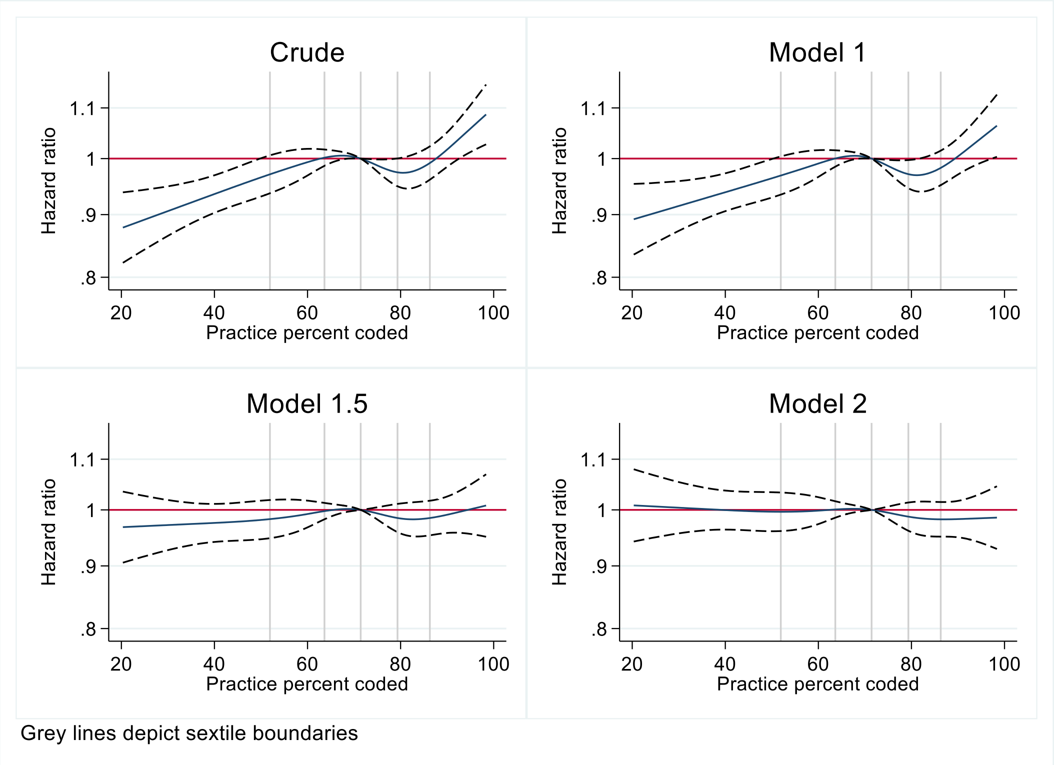

Supplementary Table 4. Fully adjusted hazard ratios (95% CI) per 10% increase in practice percent coded CKD for each outcome analysed, overall and by subgroup

	CV events	HF	Death
**All CKD**	0.973 (0.955, 0.991)*	0.936 (0.905, 0.967)*	0.999 (0.986, 1.011)
**CKD stage 3a**	0.985 (0.964, 1.007)	0.950 (0.911, 0.992)*	N/A**
**CKD stage 3b-5**	0.935 (0.893, 0.980)*	0.913 (0.846, 0.986)*	N/A**
**Diabetes**	0.963 (0.921, 1.006)	0.975 (0.906, 1.049)	N/A**
**No diabetes**	0.970 (0.948, 0.991)*	0.915 (0.879, 0.953)*	N/A**

Analysis not performed for outcomes and subgroups where assumption of linearity not supported by visual checks. (Descriptive likelihood ratio tests confirmed improved fit for linear term over 5-knot spline model in model 2 adjusted analyses, based on p-value threshold of 0.05, for all reported results, providing further justification for linearity assumption.)
Example interpretation: An adjusted hazard ratio of 0.973 for CV events represents a 6.4% reduction in the rate of CV hospitalisations for each 10% increase in practice CKD coding.

Supplementary Figure 24. Hazard ratio slope estimates (and 95% CI) for CV hospitalisations, HF hospitalisations and deaths, assuming linear differences in practice CKD coding performance compared to median practice coding, in the overall CKD population and within subgroups. *Analyses were carried out in the middle 4 sextiles of practices only (the two-thirds of most typically performing practices), with adjustment for all practice factors (model 2)*

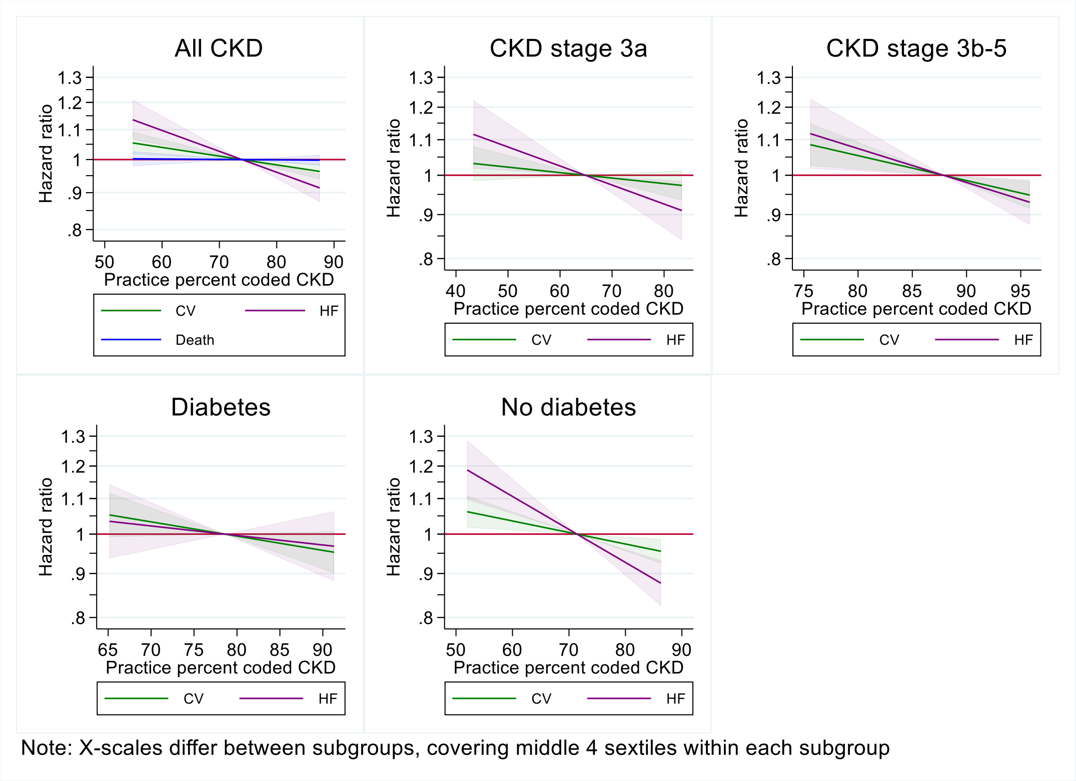

For peer review only - http://bmjopen.bmj.com/site/about/guidelines.xhtml

Supplementary Table 5. Adjusted hazard ratios for the association between practice behaviour variables and **AKI** events, sorted by point estimate

**Practice behaviour**	**HR (95% CI)**
Percent ACR/PCR test in last year in CKD (>58.7%)	0.959 (0.935, 0.984)*
Percent flu vaccination in last year in CKD (>78.8%)	0.965 (0.940, 0.990)*
Percent usage of ACEi/ARBs in hypertension (>76.6%)	0.967 (0.944, 0.990)*
Percent usage of statins in CVD (>93.0%)	0.969 (0.944, 0.995)*
Percent usage of statins in diabetes (>84.1%)	0.989 (0.964, 1.015)
Percent pneumococcus vaccination in past 5 years in CKD stages 4-5 (>12.5%)	1.008 (0.985, 1.032)
Percent meeting blood pressure target in last year in CKD (>57.8%)	1.030 (1.004, 1.055)*

Supplementary Table 6. Adjusted hazard ratios for the association between practice behaviour variables and **HF** events, sorted by point estimate

**Practice behaviour**	**HR (95% CI)**
Percent usage of ACEi/ARBs in hypertension (>76.6%)	0.935 (0.888, 0.984)*
Percent ACR/PCR test in last year in CKD (>58.7%)	0.951 (0.901, 1.004)
Percent flu vaccination in last year in CKD (>78.8%)	0.959 (0.908, 1.013)
Percent meeting blood pressure target in last year in CKD (>57.8%)	0.976 (0.926, 1.028)
Percent usage of statins in CVD (>93.0%)	0.985 (0.932, 1.042)
Percent pneumococcus vaccination in past 5 years in CKD stages 4-5 (>12.5%)	1.023 (0.973, 1.075)
Percent usage of statins in diabetes (>84.1%)	1.040 (0.984, 1.100)

Supplementary Table 7. Adjusted hazard ratios for the association between practice behaviour variables and **deaths**, sorted by point estimate with descriptive p-values

**Practice behaviour**	**HR (95% CI)**
Percent usage of statins in CVD (>93.0%)	0.950 (0.930, 0.970)*
Percent usage of ACEi/ARBs in hypertension (>76.6%)	0.965 (0.947, 0.984)*
Percent usage of statins in diabetes (>84.1%)	0.971 (0.951, 0.991)*
Percent pneumococcus vaccination in past 5 years in CKD stages 4-5 (>12.5%)	1.002 (0.983, 1.020)
Percent flu vaccination in last year in CKD (>78.8%)	1.007 (0.987, 1.027)
Percent meeting blood pressure target in last year in CKD (>57.8%)	1.010 (0.992, 1.030)
Percent ACR/PCR test in last year in CKD (>58.7%)	1.012 (0.992, 1.033)

For peer review only - http://bmjopen.bmj.com/site/about/guidelines.xhtml

Supplementary Figure 25. Results from prior NCKDA analyses, copied from manuscript reference [12]: Hazard ratios for CV events, AKI and mortality in uncoded vs coded patients, adjusted for patient age, sex, and presence of coded diabetes, hypertension and CVD, stratified by latest eGFR measurement, among patients with biochemical evidence of CKD

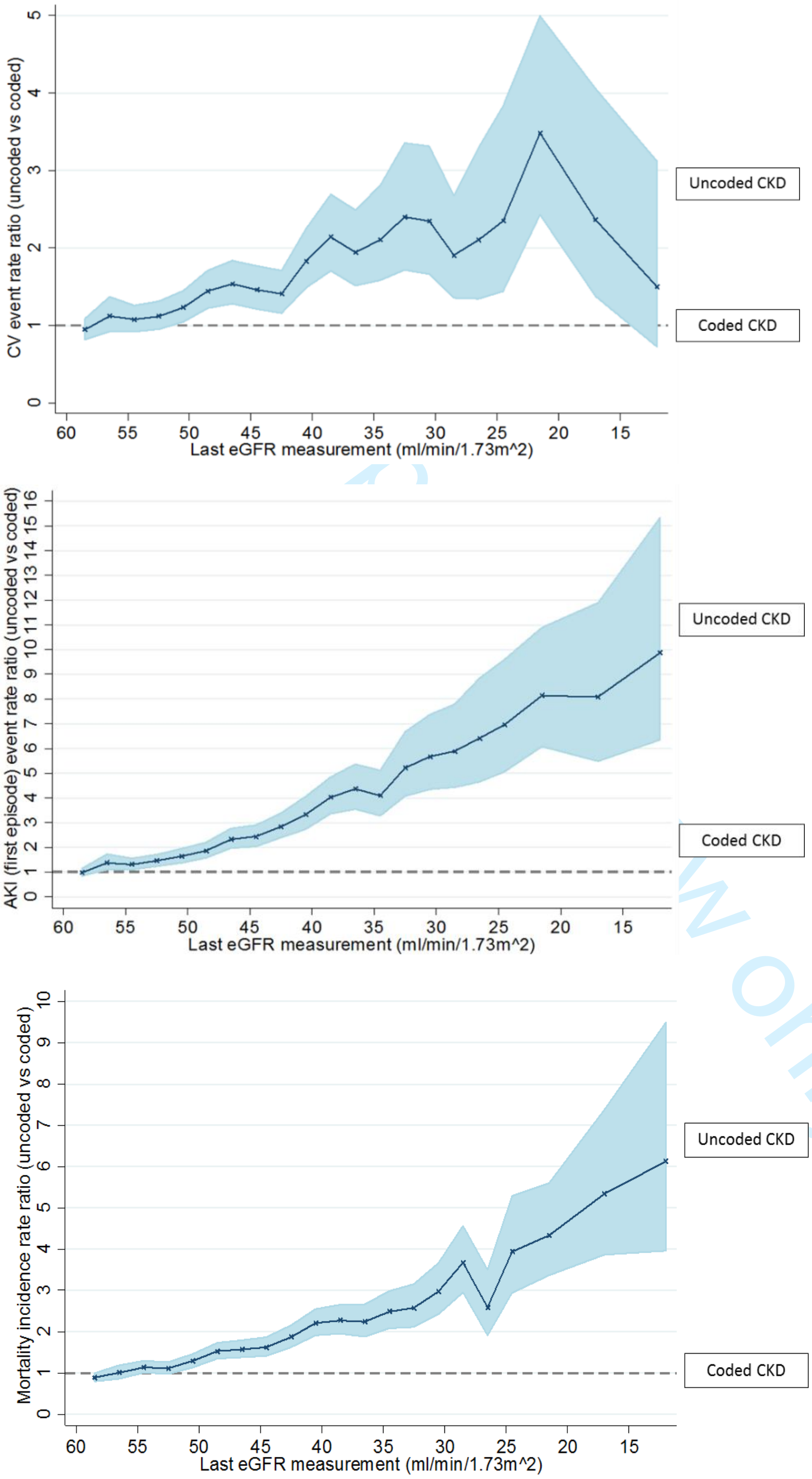

For peer review only - http://bmjopen.bmj.com/site/about/guidelines.xhtml

Supplementary Information 3. Strengthening the Reporting of Observational Studies in Epidemiology (STROBE) checklist

Section/Topic	Item #	Recommendation	Reported on page #
**Title and abstract**	1	(*a*) Indicate the study's design with a commonly used term in the title or the abstract	1, 2
		(*b*) Provide in the abstract an informative and balanced summary of what was done and what was found	2
**Introduction**			
Background/rationale	2	Explain the scientific background and rationale for the investigation being reported	5-6
Objectives	3	State specific objectives, including any prespecified hypotheses	6
**Methods**			
Study design	4	Present key elements of study design early in the paper	6
Setting	5	Describe the setting, locations, and relevant dates, including periods of recruitment, exposure, follow-up, and data collection	6-8
Participants	6	(*a*) Give the eligibility criteria, and the sources and methods of selection of participants. Describe methods of follow-up	6-8
		(*b*) For matched studies, give matching criteria and number of exposed and unexposed	N/A
Variables	7	Clearly define all outcomes, exposures, predictors, potential confounders, and effect modifiers. Give diagnostic criteria, if applicable	7-10; Supplementary Table 1
Data sources/ measurement	8*	For each variable of interest, give sources of data and details of methods of assessment (measurement). Describe comparability of assessment methods if there is more than one group	6-10
Bias	9	Describe any efforts to address potential sources of bias	10-11
Study size	10	Explain how the study size was arrived at	7
Quantitative variables	11	Explain how quantitative variables were handled in the analyses. If applicable, describe which groupings were chosen and why	8-12
Statistical methods	12	(*a*) Describe all statistical methods, including those used to control for confounding	10-12
		(*b*) Describe any methods used to examine subgroups and interactions	11
		(*c*) Explain how missing data were addressed	7-11
		(*d*) If applicable, explain how loss to follow-up was addressed	8, 10
		(*e*) Describe any sensitivity analyses	11

For peer review only - http://bmjopen.bmj.com/site/about/guidelines.xhtml

Results			
Participants	13*	(a) Report numbers of individuals at each stage of study—eg numbers potentially eligible, examined for eligibility, confirmed eligible, included in the study, completing follow-up, and analysed	12, 14, 17
		(b) Give reasons for non-participation at each stage	12, 17; Also see methods p.6-8
		(c) Consider use of a flow diagram	12
Descriptive data	14*	(a) Give characteristics of study participants (eg demographic, clinical, social) and information on exposures and potential confounders	14
		(b) Indicate number of participants with missing data for each variable of interest	See methods p.6-10
		(c) Summarise follow-up time (eg, average and total amount)	14, 17
Outcome data	15*	Report numbers of outcome events or summary measures over time	17; Also Sup. Figures 2-3, Sup. Table 2
Main results	16	(*a*) Give unadjusted estimates and, if applicable, confounder-adjusted estimates and their precision (eg, 95% confidence interval). Make clear which confounders were adjusted for and why they were included	17-19
		(*b*) Report category boundaries when continuous variables were categorized	14, 17-19
		(*c*) If relevant, consider translating estimates of relative risk into absolute risk for a meaningful time period	17-18
Other analyses	17	Report other analyses done—eg analyses of subgroups and interactions, and sensitivity analyses	17-19
**Discussion**			
Key results	18	Summarise key results with reference to study objectives	19
**Limitations**			
Interpretation	20	Give a cautious overall interpretation of results considering objectives, limitations, multiplicity of analyses, results from similar studies, and other relevant evidence	20-22
Generalisability	21	Discuss the generalisability (external validity) of the study results	20-21
**Other information**			
Funding	22	Give the source of funding and the role of the funders for the present study and, if applicable, for the original study on which the present article is based	23

*Information should be provided separately for exposed and unexposed groups in cohort studies

For peer review only - http://bmjopen.bmj.com/site/about/guidelines.xhtml

Supplementary Information 3. Strengthening the Reporting of Observational Studies in Epidemiology (STROBE) checklist

Section/Topic	Item #	Recommendation	Reported on page #
**Title and abstract**	1	(*a*) Indicate the study's design with a commonly used term in the title or the abstract	1, 2
		(*b*) Provide in the abstract an informative and balanced summary of what was done and what was found	2
**Introduction**			
Background/rationale	2	Explain the scientific background and rationale for the investigation being reported	5-6
Objectives	3	State specific objectives, including any prespecified hypotheses	6
**Methods**			
Study design	4	Present key elements of study design early in the paper	6
Setting	5	Describe the setting, locations, and relevant dates, including periods of recruitment, exposure, follow-up, and data collection	6-8
Participants	6	(*a*) Give the eligibility criteria, and the sources and methods of selection of participants. Describe methods of follow-up	6-8
		(*b*) For matched studies, give matching criteria and number of exposed and unexposed	N/A
Variables	7	Clearly define all outcomes, exposures, predictors, potential confounders, and effect modifiers. Give diagnostic criteria, if applicable	7-10; Supplementary Table 1
Data sources/ measurement	8*	For each variable of interest, give sources of data and details of methods of assessment (measurement). Describe comparability of assessment methods if there is more than one group	6-10
Bias	9	Describe any efforts to address potential sources of bias	10-11
Study size	10	Explain how the study size was arrived at	7
Quantitative variables	11	Explain how quantitative variables were handled in the analyses. If applicable, describe which groupings were chosen and why	8-12
Statistical methods	12	(*a*) Describe all statistical methods, including those used to control for confounding	10-12
		(*b*) Describe any methods used to examine subgroups and interactions	11
		(*c*) Explain how missing data were addressed	7-11
		(*d*) If applicable, explain how loss to follow-up was addressed	8, 10
		(*e*) Describe any sensitivity analyses	11

For peer review only - http://bmjopen.bmj.com/site/about/guidelines.xhtml

**Results**			
Participants	13*	(a) Report numbers of individuals at each stage of study—eg numbers potentially eligible, examined for eligibility, confirmed eligible, included in the study, completing follow-up, and analysed	12, 14, 17
		(b) Give reasons for non-participation at each stage	12, 17; Also see methods p.6-8
		(c) Consider use of a flow diagram	12
Descriptive data	14*	(a) Give characteristics of study participants (eg demographic, clinical, social) and information on exposures and potential confounders	14
		(b) Indicate number of participants with missing data for each variable of interest	See methods p.6-10
		(c) Summarise follow-up time (eg, average and total amount)	14, 17
Outcome data	15*	Report numbers of outcome events or summary measures over time	17; Also Sup. Figures 2-3, Sup. Table 2
Main results	16	(*a*) Give unadjusted estimates and, if applicable, confounder-adjusted estimates and their precision (eg, 95% confidence interval). Make clear which confounders were adjusted for and why they were included	17-19
		(*b*) Report category boundaries when continuous variables were categorized	14, 17-19
		(*c*) If relevant, consider translating estimates of relative risk into absolute risk for a meaningful time period	17-18
Other analyses	17	Report other analyses done—eg analyses of subgroups and interactions, and sensitivity analyses	17-19
**Discussion**			
Key results	18	Summarise key results with reference to study objectives	19
**Limitations**			
Interpretation	20	Give a cautious overall interpretation of results considering objectives, limitations, multiplicity of analyses, results from similar studies, and other relevant evidence	20-22
Generalisability	21	Discuss the generalisability (external validity) of the study results	20-21
**Other information**			
Funding	22	Give the source of funding and the role of the funders for the present study and, if applicable, for the original study on which the present article is based	23

*Information should be provided separately for exposed and unexposed groups in cohort studies

For peer review only - http://bmjopen.bmj.com/site/about/guidelines.xhtml

For peer review only

Association between practice coding of chronic kidney disease (CKD) in primary care and subsequent hospitalisations and death: a cohort analysis using national audit data

Journal:	*BMJ Open*
Manuscript ID	bmjopen-2022-064513.R1
Article Type:	Original research
Date Submitted by the Author:	20-Aug-2022

Complete List of Authors:	Cleary, Faye; London School of Hygiene and Tropical Medicine Department of Non-communicable Disease Epidemiology<br>Kim, Lois; University of Cambridge, Cardiovascular Epidemiology Unit<br>Prieto-Merino, David; London School of Hygiene and Tropical Medicine Department of Non-communicable Disease Epidemiology<br>Wheeler, David; University College London, Department of Renal Medicine<br>Steenkamp, Retha; UK Kidney Association<br>Fluck, Richard; Royal Derby Hospital, Department of Renal Medicine<br>Adlam, David; University of Leicester, Department of Cardiovascular Sciences and NIHR Leicester Biomedical Research Centre<br>Denaxas, S; University College London, Institute of Health Informatics; British Heart Foundation, Data Science Centre<br>Griffith, Kathryn; No affiliation, retired<br>Loud, Fiona; Kidney Care UK<br>Hull, Sally; Queen Mary University of London, Wolfson Institute of Population Health<br>Caplin, Ben; University College London, Department of Renal Medicine<br>Nitsch, Dorothea; London School of Hygiene & Tropical Medicine, Department of Non-Communicable Disease Epidmiology

**Primary Subject Heading**:	Renal medicine
Secondary Subject Heading:	Epidemiology, Public health, General practice / Family practice
Keywords:	Chronic renal failure < NEPHROLOGY, EPIDEMIOLOGY, PRIMARY CARE, PUBLIC HEALTH, Quality in health care < HEALTH SERVICES ADMINISTRATION & MANAGEMENT

SCHOLARONE™
Manuscripts

I, the Submitting Author has the right to grant and does grant on behalf of all authors of the Work (as defined in the below author licence), an exclusive licence and/or a non-exclusive licence for contributions from authors who are: i) UK Crown employees; ii) where BMJ has agreed a CC-BY licence shall apply, and/or iii) in accordance with the terms applicable for US Federal Government officers or employees acting as part of their official duties; on a worldwide, perpetual, irrevocable, royalty-free basis to BMJ Publishing Group Ltd ("BMJ") its licensees and where the relevant Journal is co-owned by BMJ to the co-owners of the Journal, to publish the Work in this journal and any other BMJ products and to exploit all rights, as set out in our licence.

The Submitting Author accepts and understands that any supply made under these terms is made by BMJ to the Submitting Author unless you are acting as an employee on behalf of your employer or a postgraduate student of an affiliated institution which is paying any applicable article publishing charge ("APC") for Open Access articles. Where the Submitting Author wishes to make the Work available on an Open Access basis (and intends to pay the relevant APC), the terms of reuse of such Open Access shall be governed by a Creative Commons licence – details of these licences and which Creative Commons licence will apply to this Work are set out in our licence referred to above.

Other than as permitted in any relevant BMJ Author's Self Archiving Policies, I confirm this Work has not been accepted for publication elsewhere, is not being considered for publication elsewhere and does not duplicate material already published. I confirm all authors consent to publication of this Work and authorise the granting of this licence.

For peer review only

Title:

Association between practice coding of chronic kidney disease (CKD) in primary care and subsequent hospitalisations and death: a cohort analysis using national audit data

Authors:

Faye Cleary[1]*, Lois Kim[2], David Prieto-Merino[1], David C Wheeler[3], Retha Steenkamp[4], Richard Fluck[5], David Adlam[6], Spiros Denaxas[7,8], Kathryn Griffith[9], Fiona Loud[10], Sally Hull[11], Ben Caplin[3], Dorothea Nitsch[1]

[1] Department of Non-Communicable Disease Epidemiology, Faculty of Epidemiology and Population Health, London School of Hygiene and Tropical Medicine, Keppel Street, London, WC1E 7HT, UK

[2] Cardiovascular Epidemiology Unit, Department of Public Health and Primary Care, University of Cambridge, Strangeways Research Laboratory, 2 Worts' Causeway, Cambridge, CB1 8RN, UK

[3] Department of Renal Medicine, University College London, Rowland Hill Street, London, NW3 2PF, UK

[4] UK Kidney Association, Brandon House Building 20a1, Southmead Road, Bristol, BS34 7RR, UK

[5] Department of Renal Medicine, Royal Derby Hospital, Uttoxeter Road, Derby, DE22 3NE, UK

[6] Department of Cardiovascular Sciences and NIHR Leicester Biomedical Research Unit, University of Leicester, UK

[7] Institute of Health Informatics, University College London, 222 Euston Road, London, NW1 2DA, UK

[8] British Heart Foundation Data Science Centre, Greater London House, 180 Hampstead Road, London, NW1 7AW, UK

[9] No affiliation, retired

[10] Kidney Care UK, 3 The Windmills, St Mary's Cl, Alton, GU34 1EF, UK

[11] Wolfson Institute of Population Health, Queen Mary University of London, London, UK

*Corresponding author

Email: faye.cleary@lshtm.ac.uk

Word count: 3,996

For peer review only - http://bmjopen.bmj.com/site/about/guidelines.xhtml

ABSTRACT

Objective To examine the association between practice percentage coding of chronic kidney disease (CKD) in primary care with risk of subsequent hospitalisations and death

Design Retrospective cohort study using linked electronic healthcare records

Setting 637 GP practices in England

Participants 167,208 patients with CKD stages 3-5 identified by 2 measures of estimated glomerular filtration rate <60 ml/min/1.73m^2, separated by at least 90 days, excluding those with coded initiation of renal replacement therapy.

Main outcome measures Hospitalisations with cardiovascular (CV) events, heart failure (HF), acute kidney injury (AKI); all-cause mortality

Results Participants were followed for (median) 3.8 years for hospital outcomes and 4.3 years for deaths. Rates of hospitalisations with CV events and HF were lower in practices with higher percentage CKD coding. Trends of a small reduction in AKI but no substantial change in rate of deaths were also observed as CKD coding increased. Compared to patients in the median performing practice (74% coded), patients in practices coding 55% of CKD cases had a higher rate of CV hospitalisations (HR 1.061 [95% CI 1.015-1.109]) and HF hospitalisations (HR 1.097 [95% CI 1.013-1.187]) and patients in practices coding 88% of CKD cases had a reduced rate of CV hospitalisations (HR 0.957 [95% CI 0.920-0.996]) and HF hospitalisations (HR 0.918 [95% CI 0.855-0.985]). We estimate that 9.0% of CV hospitalisations and 16.0% of HF hospitalisations could be prevented by improving practice CKD coding from 55% to 88%. Prescription of anti-hypertensives was the most dominant predictor of a reduction in hospitalisation rates for CKD patients, followed by albuminuria testing and use of statins.

For peer review only - http://bmjopen.bmj.com/site/about/guidelines.xhtml

Conclusions Higher levels of CKD coding by GP practices were associated with lower rates of CV and HF events, which may be driven by increased use of anti-hypertensives and regular albuminuria testing, although residual confounding cannot be ruled out.

Keywords

Epidemiology, Chronic renal failure, Primary care, Public health, Quality in health care

For peer review only

For peer review only - http://bmjopen.bmj.com/site/about/guidelines.xhtml

Strengths and limitations of this study

- A large database of 167,208 patients with biochemical evidence of CKD was used for analysis, covering 637 GP practices in England who volunteered to participate in an audit of care and which were representative of the general population in terms of age and sex.
- Risk of confounding due to patient characteristics is reduced by studying the association between practice-level (rather than patient-level) CKD coding and patient-level outcomes, where practice case-mix is not expected to differ with practice coding rates.
- Practice behaviours associated with CKD coding performance that are not believed to occur as a consequence of CKD coding may confound associations but were adjusted for as far as possible.
- Average duration of follow-up between assessment of practice coding performance and end of data collection for outcomes was limited to approximately 4 years; longer term effects of CKD coding may therefore not be captured in this study.

For peer review only

For peer review only - http://bmjopen.bmj.com/site/about/guidelines.xhtml

INTRODUCTION

Chronic kidney disease (CKD) is a growing public health problem.[1-3] Consequences of CKD include cardiovascular (CV) morbidity, acute kidney injury (AKI) and premature mortality, with increasing risks as disease progresses.[4] The burden of CKD and associated healthcare costs are increasing,[5,6] yet recognition of the disease in routine practice is often poor and varies between healthcare providers.[7,8] This may lead to delayed intervention and worsen prognosis in many CKD patients.

In the UK, computer systems used by GP practices allow electronic coding of patient clinical information, enabling consistent and specific recording and ease of access to coded data.[9] The National Institute for Health and Care Excellence (NICE) provides recommendations for regular renal function testing and CKD management in primary care,[10] and the Quality and Outcomes Framework (QOF) provides financial incentives for GPs to maintain a practice register of patients with CKD stages 3-5.[11]

The National Chronic Kidney Disease Audit (NCKDA) conducted in England and Wales in 2015-2016 found that approximately 30% of biochemically confirmed CKD stage 3-5 cases were not given an appropriate CKD code in primary care electronic healthcare records (EHRs).[8] Among patients with biochemical evidence of CKD stage 3-5, those registered with a CKD code had significantly lower rates of CV hospitalisations, AKI and mortality than those without CKD codes.[12] However, analyses only adjusted for age, sex and coded diabetes, hypertension and CV disease, due to limited data availability. The audit report cautioned therefore that a causal association cannot be established for the reported benefits of CKD coding, as results will be affected by confounding by patient health seeking behaviours and unmeasured morbidities. Some have cautioned against overdiagnosis of CKD that may fail to benefit the overall health of the population,[13] and more research is needed to study the benefits of CKD coding.

For peer review only

In attempt to overcome the issue of unmeasured confounding experienced in original analyses of the NCKDA,[12] we set out to examine the association between completeness of CKD coding of the GP practice at which patients were registered and individual adverse outcomes known to be associated with CKD. We hypothesised that practice CKD coding performance would not be associated with individual patient characteristics within practices (case-mix), thereby removing some potential confounding, and, if appropriately adjusted for practice behaviours, analysis would provide stronger evidence for a causal effect of coding of CKD on outcomes. Additional aims were to explore the role of practice behaviours that may occur as a result of CKD coding in reducing risk of adverse outcomes. Evidence that higher levels of practice CKD coding improved patient outcomes would have important ramifications for GPs and may influence policy to improve recognition of CKD in primary care.

METHODS

Study Design

We carried out a retrospective cohort study using routinely collected EHRs.

Data sources

The NCKDA database holds selected data from the EHRs of 695 GP practices in England and was used to identify a cohort of CKD patients for analysis and to define exposure variables. Data extraction ranged from March 2015 to July 2016 as practices were gradually recruited into the audit, with the majority of practices recruited by July 2015. In brief, the audit is a snapshot of care at the time point of audit data extraction. Details of the audit and data collection strategy are specified elsewhere.[8] The NCKDA database was linked to Hospital Episode Statistics (HES) holding information on all hospital admissions in England, and Office for National Statistics (ONS) mortality data, to follow up patients for adverse outcomes. Linkage was carried out by NHS Digital using NHS number, and hospital record information with pseudo-anonymised linkage IDs were provided for analysis.

For peer review only

The NCKDA research database has national ethical approval (REC reference: 17/LO/0049), section 251 approval (CAG reference number: 17CAG0058). NCKDA steering group approval[14] and LSHTM ethics approval (reference: 21689) were granted for this analysis.

Study population

The analysis cohort included all adult patients in the NCKDA database extracted from eligible GP practices in England and with biochemical evidence of CKD (hereafter referred to simply as "CKD" and/or "confirmed CKD"), defined as at least 2 records of estimated glomerular filtration rate (eGFR) <60 ml/min/m^2 separated by at least 90 days, with the most recent measure recorded within the last 2 years prior to data extraction and excluding any patients with coded initiation of renal replacement therapy (RRT).

Primary exposure

At the time of data extractions (2015-2016), Read codes were used to electronically record patient findings in GP computer systems.[15] Variables defined based on eGFR use the isotope-dilution mass spectroscopy (IDMS) calibrated Modification of Diet in Renal Disease (MDRD) study equation, the standard GFR-estimating equation in use during the period of data collection.

Practice CKD coding performance was characterised as the percentage of CKD patients in a practice with a CKD stages 3-5 Read code, hereafter referred to as practice CKD coding (performance) or percent coded CKD. Practice CKD coding performance was defined at practice data extraction, which marked the index date for commencement of follow-up for outcomes.

Practices with fewer than 50 total CKD cases were excluded from analysis due to anticipated excess noise in measurement of the primary exposure.

Outcomes

Four outcomes of interest were studied: (i) hospitalisation for CV events, (ii) hospitalisation for heart failure (HF), (iii) hospitalisation with AKI, and (iv) all-cause mortality, defined in **Supplementary Table**

For peer review only - http://bmjopen.bmj.com/site/about/guidelines.xhtml

1. Follow-up began at the time of practice data extraction and was capped at 1st March 2019 for hospital outcomes and 1st September 2019 for deaths.

Practice features

Features of a practice that may confound the association between practice CKD coding and patient adverse outcomes were defined and categorised as: those reflecting overall practice risk profile; practice testing behaviours for CKD; and characteristics of the identified practice CKD population. Practice features that may improve CKD outcomes, some of which may lie on the causal pathway between practice CKD coding and patient adverse outcomes were also defined. Practice percentage variables were defined by summing the number of patients meeting relevant risk factor criteria in each practice and dividing by relevant practice denominators. Practice list size data were used to determine size of the adult population in each practice.

Patient-level risk factors identified in NCKDA data (and used to calculate practice percentages) were defined based on presence of any relevant Read code in the EHR prior to data extraction and included:

- Diabetes – any diabetes code not superseded by a diabetes resolved code
- Hypertension – any hypertension code
- Cardiovascular disease (CVD) – any CVD code
- CKD stages 3b-5 – confirmed CKD and latest eGFR < 45
- Statin use - any statin prescription or contraindication code
- Anti-hypertensive use - any prescription or contraindication code for angiotensin-converting enzyme inhibitors (ACEi) or angiotensin-II receptor blockers (ARBs)
- Blood pressure (BP) targets met - last BP within target range within last year before data extraction: SBP < 130 mmHg and DBP < 80 mmHg in those with diabetes or last urine albumin to creatinine ratio (ACR) ≥ 70 mg/mmol or last protein to creatinine ratio (PCR) ≥ 100 mg/mmol; SBP < 140 mmHg and DBP < 90 mmHg in all other patients

For peer review only - http://bmjopen.bmj.com/site/about/guidelines.xhtml

- Flu vaccination – any influenza vaccination code in the last year
- Pneumococcal vaccination – any pneumococcal vaccination code in the last 5 years

Additional patient-level risk factors not available in NCKDA data were defined using HES data and included:

- Recent chronic obstructive pulmonary disease (COPD) admission - admission in last 3 years prior to (NCKDA) practice data extraction with a COPD ICD-10 code (J44) as primary diagnosis
- Recent cancer admission - admission in last 3 years prior to (NCKDA) practice data extraction with a cancer ICD-10 code (C00-C97, excluding non-melanoma skin cancers C44) as primary diagnosis

Practice characteristics reflecting overall practice risk profile:

GPs' awareness on how to identify CKD may depend on the overall burden of conditions associated with CKD in their practice. *Practice prevalence of diabetes, hypertension and CVD* were determined by summing the number of adult patients meeting patient-level co-morbidity definitions, with adult population size as denominator. Practice list size data stratified by age and sex were used to determine *mean practice age* and *percent of adults that were male*. Practice deprivation was summarised using the *median rank of the index of multiple deprivation (IMD) score* among all patients extracted from the GP practice (which was limited to patients with CKD risk factors, creatinine assessments or renal codes).

Practice testing behaviours:

Testing behaviours which may impact the patient vintage (i.e. the underlying duration of CKD at detection by the GP) and the types of CKD patients selected for analysis were also defined within practices, including *percentage of diabetes patients with a GFR test in the last year*, *percentage of*

For peer review only

CKD patients with a GFR test in the last year and *percentage of the practice adult population with confirmed CKD*.

Practice characteristics of the detected CKD population:

Underlying practice morbidity and testing behaviours may impact the types of CKD patients detected and therefore included in analysis. *Percent CKD stages 3b-5*, *percent recent COPD admission* and *percent recent cancer admission* were defined using patient-level risk factor definitions, with number of total CKD cases as denominator.

Practice behaviours that may improve CKD outcomes:

Practice behaviours expected to be related to CKD coding, some of which may be on the causal pathway from CKD to improved outcomes were defined as: *percent usage of ACEi/ARBs in hypertension*, *percent usage of statins in diabetes*, *percent usage of statins in CVD*, *percent meeting blood pressure target in last year in CKD*, *percent ACR/PCR test in last year in CKD*, *percent flu vaccination in last year in CKD*, and *percent pneumococcus vaccination in past 5 years in CKD stages 4-5*. Practice behaviour variables were dichotomised at the median value.

Statistical methods

Baseline characteristics of the study population were summarised by sextile of practice percent coded CKD to determine balance in patient characteristics according to primary exposure. Practice characteristics were also summarised by sextile of practice CKD coding to identify any associations with other practice characteristics.

Main Cox regression analyses:

Cox proportional hazards regression (time to first event) was used to evaluate the association between practice CKD coding and each of the 4 patient outcomes. Hospitalisation outcomes were censored for death. A 5-knot spline was used for the primary exposure, providing flexibility to demonstrate the nature of association between practice coding and outcomes across the spectrum

For peer review only - http://bmjopen.bmj.com/site/about/guidelines.xhtml

of practice CKD coding performance, without overfitting. The following adjustments for practice characteristics (included as continuous covariates) were carried out sequentially:

Model 1: adjusted for ***practice characteristics reflecting overall practice risk profile***

[Primary analysis, planned a priori]

Model 2: adjusted for ***practice characteristics reflecting overall practice risk profile, practice testing behaviours,*** and ***practice characteristics of the detected CKD population***

[Secondary analysis, data-driven]

Adjusted hazard ratio (HR) curves for outcomes with 95% confidence intervals (CIs) were plotted across the spectrum of practice CKD coding, compared to average (median) practice CKD coding. Attributable fractions for the number of (first) events preventable by the median follow-up time among CKD patients in practices at lower coding levels (17th percentile, bottom of sextile 2) if such practices instead coded at higher coding levels (83rd percentile, top of sextile 5) were estimated under assumption of causality following adjusted Cox regression (model 2), detailed in **Supplementary Information 1**.

Additional analyses with a single linear continuous covariate for percent coded CKD were carried out after visual inspection of an approximately linear relationship for some model 2 HR curves, allowing a more convenient clinical interpretation. These models were restricted to sextiles 2-5 of practice coding only (representing the 67% of most averagely performing practices) where linear trends were most apparent. Descriptive likelihood ratio tests were used to assess improvement in model fit using spline terms vs. a single linear covariate.

Subgroup analyses:

Subgroup analyses were carried out by diabetes status and by CKD severity (Stage 3a, Stage 3b-5). Practice percent coded CKD was re-calculated within each subgroup for analysis, since coding behaviour differed substantially between subgroups.

For peer review only - http://bmjopen.bmj.com/site/about/guidelines.xhtml

Analyses of practice behaviours that may improve CKD outcomes:

Further Cox regression analyses aimed to identify practice behaviours associated with improvements in all 4 patient outcomes , with adjustment for all (model 2) confounders as well as ***practice behaviours that may improve CKD outcomes***. Practice CKD coding covariates were excluded from analysis to identify practice factors most predictive of outcomes, regardless of CKD coding performance and not conditional on CKD coding.

Patient and public involvement

Kidney Care UK supported the research questions, grant applications and related record linkage applications of the NCKDA. After NCKDA discontinuation, Kidney Care UK helped with ethics and section 251 permissions to maintain database access for research purposes. A patient representative (Fiona Loud) was involved in the NCKDA from inception, is a co-author and critically reviewed content of this paper.

RESULTS

Data completeness

Of 695 practices in England captured in the NCKDA database, 637 practices (92%) met criteria for analysis (at least 50 CKD cases), covering 99% of all CKD patients from the original database (n=167,208) (**Figure 1**). CKD coding rates did not differ after excluding ineligible practices, overall or by subgroup, but sample sizes were smaller in some subgroups after excluding practices with fewer than 50 CKD cases (**Supplementary Table 2**).

For peer review only - http://bmjopen.bmj.com/site/about/guidelines.xhtml

Patient characteristics

Study population characteristics were generally well balanced between sextiles of practice CKD coding (**Table 1**). There were trends of slightly higher rates of diabetes, hypertension and CVD coding and lower eGFR in patients in the highest coding practices (sextile 6) and slightly lower rates of co-morbidities and higher eGFR in the lowest coding practices (sextile 1), indicating potential differences in either true underlying morbidity or risk factor coding in patients in practices performing at the extremes. Median month of data extraction and resulting follow-up duration were well balanced between sextiles, suggesting good balance in seasonal coverage.

Practice characteristics

Median practice percent coded CKD was 73.9% in the overall CKD population. It was higher in CKD stages 3b-5 (87.9%) than CKD stage 3a (64.8%), and higher in those with diabetes (78.6%) than those without diabetes (71.4%). Coding performance was more variable in early stage CKD and non-diabetic CKD (**Supplementary Figure 1**).

Practice characteristics were generally well balanced in coding sextiles 2-5 (**Table 2**). At the extremes (sextiles 1 and 6), higher coding practices had on average higher prevalence of coded co-morbidities, were more deprived, and performed more regular and complete GFR testing in those at risk. Practice behaviours that may improve CKD outcomes showed trends of improved performance in higher coding practices, across the spectrum of CKD coding. In particular, ACR/PCR testing was substantially higher in higher coding practices, and flu vaccination rates were also markedly higher.

For peer review only - http://bmjopen.bmj.com/site/about/guidelines.xhtml

Table 1. Study population characteristics by practice coding sextile

**Practice coding sextile (S)**	**S1**	**S2**	**S3**	**S4**	**S5**	**S6**
**Practice percent coded CKD**	**<54.8%**	**54.8% - 65.5%**	**65.5% - 73.9%**	**73.9% - 80.7%**	**80.7% - 87.5%**	**≥87.5%**
Number of CKD patients	N = 29,389	N = 30,360	N = 28,248	N = 28,824	N = 26,731	N = 23,656
Coded CKD	13,105 (44.6%)	18,505 (61.0%)	19,744 (69.9%)	22,326 (77.5%)	22,524 (84.3%)	21,728 (91.8%)
Age, mean (SD)	76.7 (11.2)	77.1 (11.0)	77.0 (10.9)	77.2 (10.8)	77.1 (10.7)	77.5 (10.7)
Male	11,686 (39.8%)	12,240 (40.3%)	11,464 (40.6%)	11,709 (40.6%)	10,664 (39.9%)	9,493 (40.1%)
Diabetes	7,013 (23.9%)	7,657 (25.2%)	6,887 (24.4%)	7,288 (25.3%)	6,544 (24.5%)	6,148 (26.0%)
Hypertension	19,475 (66.3%)	20,749 (68.3%)	19,300 (68.3%)	19,798 (68.7%)	18,860 (70.6%)	17,207 (72.7%)
CVD	9,324 (31.7%)	9,945 (32.8%)	9,087 (32.2%)	9,642 (33.5%)	8,819 (33.0%)	8,086 (34.2%)
Biochemical CKD Stage						
3a	18,927 (64.4%)	19,201 (63.2%)	17,938 (63.5%)	18,151 (63.0%)	16,858 (63.1%)	14,774 (62.5%)
3b	8,384 (28.5%)	8,938 (29.4%)	8,254 (29.2%)	8,576 (29.8%)	7,847 (29.4%)	7,089 (30.0%)
4	1,838 (6.3%)	1,937 (6.4%)	1,812 (6.4%)	1,831 (6.4%)	1,796 (6.7%)	1,572 (6.6%)
5	240 (0.8%)	284 (0.9%)	244 (0.9%)	266 (0.9%)	230 (0.9%)	221 (0.9%)
Time since last GFR measure (months), median (IQR)	5.0 (2.0, 9.4)	4.5 (1.8, 8.7)	4.6 (1.9, 8.6)	4.4 (1.8, 8.5)	4.3 (1.8, 8.3)	4.5 (1.9, 8.3)
Prior COPD admission in last 3 years	346 (1.2%)	352 (1.2%)	365 (1.3%)	366 (1.3%)	323 (1.2%)	285 (1.2%)
Prior cancer admission in last 3 years	1,278 (4.3%)	1,332 (4.4%)	1,266 (4.5%)	1,212 (4.2%)	1,122 (4.2%)	1,044 (4.4%)
Follow-up duration (months) for HES outcomes (months), median (IQR)	47 (36, 47)	47 (36, 47)	45 (36, 47)	46 (33, 47)	45 (35, 47)	45 (33, 47)

For peer review only - http://bmjopen.bmj.com/site/about/guidelines.xhtml

Table 2. Practice characteristics by practice coding sextile

**Practice coding sextile**	**S1**	**S2**	**S3**	**S4**	**S5**	**S6**
**Practice percent coded CKD**	**<54.8%**	**54.8% - 65.5%**	**65.5% - 73.9%**	**73.9% - 80.7%**	**80.7% - 87.5%**	**≥87.5%**
Number of practices	N = 106	N = 106	N = 106	N = 107	N = 106	N = 106
Practice percent coded CKD, mean (SD)	44.2% (9.8%)	61.2% (3.5%)	70.0% (2.4%)	77.5% (2.0%)	84.1% (2.2%)	91.7% (3.1%)
Practice characteristics reflecting practice risk profile						
Mean adult age, mean (SD)[M1,M2,CP]	48.9 (4.1)	49.5 (4.7)	49.1 (3.2)	49.2 (3.9)	49.5 (3.8)	49.4 (3.4)
Percent male, mean (SD) [M1,M2,CP]	49.3% (1.9%)	48.6% (2.6%)	49.3% (1.9%)	49.1% (2.0%)	49.3% (2.0%)	49.1% (2.5%)
Practice median rank of IMD, median (IQR) [M1,M2,CP,**]	18,236 (9,574, 23,480)	17,887 (11,835, 22,921)	17,363 (9,987, 24,460)	17,114 (10,392, 21,711)	15,793 (10,946, 21,345)	16,555 (12,742, 22,962)
Diabetes prevalence, mean (SD) [M1,M2,CP]	5.6% (1.3%)	5.9% (1.4%)	6.0% (1.5%)	6.0% (1.5%)	6.0% (1.3%)	6.3% (1.5%)
Hypertension prevalence, mean (SD) [M1,M2,CP]	16.1% (4.1%)	16.8% (3.9%)	16.6% (3.7%)	17.1% (4.1%)	17.6% (3.7%)	18.7% (4.2%)
CVD prevalence, mean (SD) [M1,M2,CP]	5.5% (1.8%)	5.9% (2.4%)	5.8% (1.6%)	6.0% (1.8%)	6.2% (1.6%)	6.4% (1.8%)
Practice testing behaviours						
Percent GFR test in last year in diabetes, mean (SD) [M2,CP]	86.9% (12.8%)	89.8% (5.3%)	89.7% (4.7%)	89.7% (5.2%)	89.7% (10.4%)	90.3% (5.0%)
Median months since last GFR test in diabetes, median (IQR)	4.6 (3.7, 5.4)	4.2 (3.5, 5.0)	4.2 (3.7, 5.1)	4.4 (3.7, 5.1)	3.8 (3.3, 4.7)	4.4 (3.8, 5.1)
Percent GFR test in last year in CKD, mean (SD) [M2,CP]	83.3% (12.7%)	86.6% (4.9%)	87.0% (4.7%)	87.2% (5.6%)	86.6% (10.6%)	88.3% (5.5%)
Median months since last GFR test in CKD, median (IQR)	4.8 (4.1, 5.7)	4.6 (4.1, 5.2)	4.7 (4.0, 5.2)	4.5 (3.8, 5.3)	4.2 (3.6, 5.0)	4.4 (3.9, 5.2)
Biochemical CKD prevalence, mean (SD) [M2,CP]	4.2% (1.9%)	4.0% (1.5%)	3.9% (1.4%)	3.8% (1.5%)	4.0% (1.4%)	4.0% (1.5%)

For peer review only - http://bmjopen.bmj.com/site/about/guidelines.xhtml

Practice characteristics of the detected CKD population						
Percent CKD stages 3b-5, mean (SD) [M2,CP]	37.0% (5.4%)	37.5% (4.9%)	37.5% (4.8%)	38.3% (5.2%)	38.0% (4.3%)	38.0% (5.0%)
Percent recent COPD admission, mean (SD) [M2,CP]	1.2% (1.0%)	1.2% (0.7%)	1.4% (0.9%)	1.3% (1.0%)	1.3% (1.1%)	1.2% (1.0%)
Percent recent cancer admission, mean (SD) [M2,CP]	4.3% (1.7%)	4.4% (1.9%)	4.4% (1.8%)	4.2% (1.8%)	4.1% (1.8%)	4.4% (2.0%)
Practice behaviours that may improve CKD outcomes						
Percent usage of ACE/ARBs in hypertension, mean (SD)[CP]	76.0% (6.2%)	75.5% (6.1%)	77.1% (5.3%)	75.9% (5.9%)	76.5% (4.6%)	78.2% (5.5%)
Percent usage of statins in diabetes, mean (SD) [CP]	82.2% (5.8%)	83.2% (5.8%)	82.9% (5.5%)	84.2% (5.8%)	84.8% (5.3%)	85.0% (5.5%)
Percent usage of statins in CVD, mean (SD) [CP]	91.4% (3.4%)	92.2% (3.5%)	92.8% (2.9%)	93.0% (2.6%)	93.1% (2.6%)	93.0% (3.5%)
Percent meeting blood pressure target in last year in CKD, mean (SD) [CP]	56.8% (7.4%)	57.0% (6.2%)	57.9% (7.0%)	56.8% (7.4%)	58.3% (7.4%)	60.2% (6.7%)
Percent ACR/PCR test in last year in CKD, mean (SD) [CP]	40.1% (13.3%)	49.1% (13.4%)	55.9% (11.9%)	61.3% (13.3%)	62.5% (17.0%)	68.9% (15.7%)
Percent flu vaccination in last year in CKD, mean (SD) [CP]	73.3% (15.4%)	77.0% (7.4%)	77.6% (5.7%)	78.8% (5.6%)	79.3% (9.5%)	80.5% (6.3%)
Percent pneumococcus vaccination in past 5 years in CKD stages 4-5, mean (SD) [CP]	15.3% (14.9%)	18.2% (16.8%)	15.2% (14.5%)	19.1% (17.0%)	17.1% (15.7%)	18.2% (18.6%)

*Prevalence statistics use adult population as denominator
** IMD is ranked for all Lower Super Output Areas (LSOAs) in England (1=worst, 32,844=best); summary statistics are based on IMD rankings of patients extracted from practices only and should be interpreted carefully
[M1] indicates variables included in statistical analysis model 1 (primary analysis)
[M2] indicates variables included in statistical analysis model 2 (secondary analysis)
[CP] indicates variables included in statistical analysis of practice behaviour variables

For peer review only

Outcomes

Of 167,208 CKD patients identified from the NCKDA database, after national linkage we found that 563 deaths had occurred but had not been reported on primary care systems at date of data extraction, leaving 166,645 eligible for outcomes analysis. Median follow-up duration was 3.8 years (range 1 day – 3.9 years) for HES outcomes and 4.3 years (range 1 day – 4.4 years) for mortality, with no meaningful differences in follow-up between sextiles. Crude event rates by sextile are shown in **Supplementary Tables 3-4** and **Supplementary Figures 2-3**.

Main adjusted Cox regression analyses

Figure 2 demonstrates how individual patient risks of the 4 studied outcomes differ according to practice CKD coding, compared to a patient in an averagely performing practice. In model 1 analyses, inverted S-shaped HR curves suggest that confounding at the extremes of practice coding may distort the association between practice CKD coding and adverse outcomes. After further adjustments (model 2), curves become flatter (approaching linearity). Wide CIs at lower levels of CKD coding reflect poor precision in HR estimates due to sparse data. (Crude analyses and additional sequential adjustments in the CKD population and subgroups [detailed in **Supplementary Information 2**] are shown in **Supplementary Figures 4-23.)**

There are strong trends, particularly among sextiles 2-5 (55% to 88% coded), of reduced rates of outcomes with improved practice CKD coding in fully adjusted analyses (model 2). Compared to patients in the averagely performing practice (74% coded), patients in practices coding only 55% of CKD cases had a significantly higher rate of CV hospitalisations (HR 1.061 [95% CI 1.015-1.109]) and HF hospitalisations (HR 1.097 [95% CI 1.013-1.187]). Patients in practices coding 88% of CKD cases had a significantly reduced rate of CV hospitalisations (HR 0.957 [95% CI 0.920-0.996]) and HF hospitalisations (HR 0.918 [95% CI 0.855-0.985]), compared to the averagely performing practice. The percentage of preventable events over a period of 3.8 years (median follow-up time) for an improvement in practice coding from 55% to 88% of CKD cases (attributable fraction) was 9.0% for

For peer review only - http://bmjopen.bmj.com/site/about/guidelines.xhtml

first CV hospitalisations and 16.0% for first HF hospitalisations, under assumption of causality after Cox modelling (model 2). Trends of a small reduction in AKI but no substantial change in rate of deaths were also observed as CKD coding increased. (Additional results of analysis of a single linear practice coding term [where appropriate] are shown in **Supplementary Table 5** and **Supplementary Figure 24**.)

Subgroup analyses showed a steeper reduction in CV hospitalisations and HF hospitalisations with increasing practice coding among CKD stages 3b-5 than in CKD stage 3a both in spline regression analyses and those assuming linear effects of practice CKD coding (**Supplementary Figures 5, 6, 10, 11 and 24**).

Practice behaviours analyses

Analysis of the association between practice behaviours and CV hospitalisations adjusted for all confounders showed a significant reduction in rate of CV hospitalisations for CKD patients belonging to practices with greater than average usage (median 76.6%) of ACEi/ARBs in hypertension compared to practices with lower than average usage (HR 0.956 [95% CI 0.929, 0.983]) (**Table 3**). Practice ACR/PCR testing in CKD was also associated with a reduction in rates of CV hospitalisations in CKD (HR 0.968 [95% CI 0.939-0.998]). Results for analyses of AKI, HF and deaths are available in **Supplementary Tables 6-8**. In brief, usage of ACEi/ARBs was the most consistently dominant predictor, being strongly associated with a reduction in events across all outcomes. This was followed by ACR/PCR testing, which was associated with a reduction in all outcomes except deaths, and usage of statins in CVD, which was associated with a reduction in rate of CV and AKI events.

For peer review only - http://bmjopen.bmj.com/site/about/guidelines.xhtml

Table 3. Adjusted HRs for the association between practice behaviour variables and CV events, sorted by point estimate

Practice behaviour	HR (95% CI)
Percent usage of ACEi/ARBs in hypertension (>76.6%)	0.956 (0.929, 0.983)*
Percent ACR/PCR test in last year in CKD (>58.7%)	0.968 (0.939, 0.998)*
Percent usage of statins in CVD (>93.0%)	0.972 (0.942, 1.003)
Percent pneumococcus vaccination in past 5 years in CKD stages 4-5 (>12.5%)	0.982 (0.955, 1.010)
Percent meeting blood pressure target in last year in CKD (>57.8%)	0.992 (0.963, 1.028)
Percent usage of statins in diabetes (>84.1%)	0.995 (0.965, 1.026)
Percent flu vaccination in last year in CKD (>78.8%)	0.998 (0.968, 1.028)

Analysis adjusted for practice characteristics: mean age, percent male, median rank of index of multiple deprivation, diabetes prevalence, hypertension prevalence, CVD prevalence, percent of CKD cases at stages 3b-5, percent of CKD patients admitted for COPD in last 3 years, percent of CKD patients admitted for cancer in last 3 years, percent GFR test in last year in diabetes, percent GFR test in last year in CKD, percent of adult population with CKD

DISCUSSION

Higher levels of practice CKD coding were associated with lower rates of hospitalisation for CV and HF events among patients with confirmed CKD, after adjusting for practice characteristics. Reductions in hospitalisation rates were strongest in CKD stages 3b-5, although greatest opportunities for improvement are in CKD stage 3a where practice variation in coding was much wider. There was no difference in death rates according to practice CKD coding (although the relationship was less clear among CKD stages 3b-5). Findings were limited by duration of follow-up with longer-term benefits of CKD coding not yet apparent. Practice behaviours associated with CKD coding including usage of ACEi/ARB therapy and ACR/PCR testing were independent predictors of reduction in hospitalisation rates.

For peer review only - http://bmjopen.bmj.com/site/about/guidelines.xhtml

There are very limited studies looking at the impact of recognition and diagnostic coding of CKD in primary care as many health systems use coded disease as opposed to laboratory records to identify patients. It is possible that some patients with 2 eGFR measures < 60 ml/min/1.73m^2 more than 3 months apart are not recognized by GPs as having CKD because of concerns of overdiagnosis, for example in elderly patients without hypertension or in patients recovering from AKI, despite these patients meeting the accepted definition of CKD. Furthermore, numerous studies have demonstrated disparities in CKD coding efforts with younger patients, those from deprived backgrounds, and ethnic minorities being less commonly coded than their counterparts,[7,16] leading to concerns around equity of care. Recent studies have shown an association between CKD coding and interventions known to reduce CV risk such as prescription of statins and anti-hypertensive agents,[16,17] and CKD coding may play a role in triggering further long-term treatment efforts with potential to reduce patient risks. Our study identified a reduced burden of CV and HF hospitalisations for practices coding more CKD, and a reduced burden of hospitalisations for practices providing more interventions (associated with CKD coding) that are likely to improve CKD outcomes.

A key strength of this study is the large sample size, including data from 167,208 CKD patients. Data were extracted from GP practices in England with a similar age-sex distribution to the whole population, so findings are likely to be generalisable to the wider population. By studying the association between practice-level CKD coding and patient-level outcomes, we were able to eliminate a lot of confounding due to individual patient characteristics that would be present in a conventional study design using patient-level exposure with unmeasured confounders. This was demonstrated by the balanced risk profile in patient characteristics observed across sextiles (which is likely to extend also to unmeasured characteristics). This is a major benefit over original analyses of the NCKDA[12] (**Supplementary Figure 25**) which showed a very strong association between individual patient CKD coding and risk of outcomes (CV events, AKI, death) but with a high risk of

For peer review only - http://bmjopen.bmj.com/site/about/guidelines.xhtml

confounding due to coding efforts being associated with perceived patient risk, and some potentially important risk factors missing from the database.

A potential weakness is that included practices had volunteered to participate in an audit of care. CKD coding may have been higher in recruited practices than the general population which may have impacted on estimated strengths of associations; benefits of coding in the wider practice population may be larger than estimated. Risk factor evaluation mostly relied on co-morbidity coding and it is not clear whether small differences in risk factor prevalence reflected true morbidity or GP behaviour. Assessment of eligibility of patients for analysis also relied on availability of repeat creatinine tests over time, which may depend on patient risk, and earlier stage CKD cases or more severe cases managed solely in secondary care may be disproportionately missing. Nevertheless, this identified CKD population may stand to benefit most imminently from improvements in primary care, assuming GPs target further coding efforts to patients already identified as at risk and with creatinine test results compatible with CKD. While there was a small signal of more frequent creatinine testing with increasing practice CKD coding, this was only at the extremes, and distribution of CKD severity appeared generally very well balanced across practice coding sextiles. Practice characteristics were analysed differently depending on whether they were likely to confound analyses or lie on the causal pathway, however we could not verify if our assumptions were reasonable, and misspecification could affect reliability of conclusions. For example, practice management of hypertension with ACEi/ARB therapy may plausibly confound analyses (if hypertension management and CKD coding share a common cause, such as practice funding or clinical expertise) or lie on the causal pathway (if management of hypertension occurs as a consequence of CKD coding). Our findings for AKI are likely affected by outcome misclassification as hospital codes were used to detect AKI events, which may have led to underestimation of the number of events and lack of power to detect an association. We did not have enough dialysis events to allow evaluation of the impact of practice coding on outcomes. These data precede the

For peer review only - http://bmjopen.bmj.com/site/about/guidelines.xhtml

use of SLGT2-inhibitor drug treatment in heart failure and albuminuric kidney disease in UK primary care.

Conclusions

Rates of CV and HF events were lower for patients belonging to practices coding more CKD, supporting the argument that CKD coding in primary care may contribute to improvement in patient outcomes. While the presence of unmeasured confounding cannot be ruled out, this is in agreement with other studies conducted in this setting.[16,17,18] High quality evidence supporting our findings is available from clinical trials and systematic reviews which underline the benefits of use of interventions in early stage CKD, including ACEi/ARB therapy to control hypertension and statin therapy to reduce CV risk.[19-21] This study suggests that reductions in key adverse events for patients with CKD could be made by improvements to GP practice identification and coding of CKD as these are associated with subsequent care efforts that are known to prevent poor outcomes.

For peer review only

For peer review only - http://bmjopen.bmj.com/site/about/guidelines.xhtml

FUNDING

This research was funded by the UK MRC, grant reference MR/N013638/1. The funding body had no role in the design of the study, collection, analysis, or interpretation of data and no role in writing of the manuscript.

COMPETING INTERESTS

David C Wheeler has an ongoing consultancy contract with AstraZeneca and has received honoraria, consultancy fees or speaker fees from Amgen, Astellas, Bayer, Boehringer Ingelheim, GlaxoSmithKline, Gilead, Janssen, Napp/Mundipharma, Merck Sharp and Dohme, Tricida, Vifor and Zydus. David Adlam has received research funding from Abbott vascular to support a clinical research fellow; he has also received funding from AstraZeneca inc. for unrelated research and has undertaken consultancy for General Electric inc. to support research funds. Dorothea Nitsch reports grants unrelated to this work from the National Institute for Health Research, Medical Research Council (MRC), the Health Foundation and GlaxoSmithKline.

AUTHOR CONTRIBUTIONS

DN, FC, BC, SH, LK and DP planned the analysis. FC conducted analysis, interpreted results and reported the work. DN, LK, DP, DCW, SH, RS, RF, DA, SD, KG, FL and BC contributed insights in the interpretation of results, provided updates to manuscript text, and read and approved the final manuscript. FC is the guarantor and accepts full responsibility for the finished work and conduct of the study, had access to the data, and controlled the decision to publish.

ACKNOWLEDGMENTS

We thank all the patients who have provided their data to the audit analyses, and patient organisations, especially KidneyCare UK and the National Kidney Foundation, and those involved in the set up and conduct of the National Chronic Kidney Disease Audit, which provided a large database of patients with CKD, without which this research would not have been possible. This

For peer review only - http://bmjopen.bmj.com/site/about/guidelines.xhtml

includes (apart from the listed authors): Matthew Harker, Yvonne Silove, Tasneem Hoosain, Nick Wilson, Ronnie Moodley, Chris Gush, Liam Smeeth, Ron Cullen, Fergus Caskey, Andy Syme, Richard Gunn, Paul Wright, Hugh Gallagher, Sion Edwards, Nick Palmer, Anita Sharma, Kate Cheema, Kathleen Mudie.

RECORD REPORTING CHECKLIST

This article adheres to RECORD reporting guidelines for observational studies using routinely collected data. A checklist is provided in **Supplementary Information 3**.

DATA SHARING

The data that support the findings of this study are stored at University College London (UCL) but restrictions apply to the availability of these data, which were used under license for the current study, and so are not publicly available.

ETHICS STATEMENT

The National Chronic Kidney Disease Audit (NCKDA) research database has national ethical approval (REC reference: 17/LO/0049), section 251 approval (CAG reference number: 17CAG0058). NCKDA steering group approval and London School of Hygiene and Tropical Medicine (LSHTM) ethics approval (reference: 21689) were granted for this analysis. The NCKDA was implemented in England and Wales in 2015-2016 to review variation in patient care with regards to the identification and management of patients with chronic kidney disease in primary care. It involved extraction of GP practice medical records for a very large number of patients, with linkage to hospital outcomes and mortality databases. The NCKDA database is now being used for further research on which aspects of primary care management improve long-term patient outcomes of patients at risk of or with kidney disease. Research ethics committee approval has been obtained to use pseudonymised clinical data from the NCKDA for research purposes. The pseudonymised database is held at LSHTM for analysis. There are no personal identifiers (such as names, NHS-numbers or addresses) which

For peer review only - http://bmjopen.bmj.com/site/about/guidelines.xhtml

allow a direct link between a person's name and their health information held in this clinical dataset; instead each record has an LSHTM study-id (pseudo-identifier) attached. A study-id is a unique number added to an individual's record for use in the study. The information on NHS numbers and corresponding study-id (pseudo-identifiers) are held separately at University College London in a super-secure server.

For peer review only

For peer review only - http://bmjopen.bmj.com/site/about/guidelines.xhtml

REFERENCES

1. GBD Chronic Kidney Disease Collaboration. Global, regional, and national burden of chronic kidney disease, 1990-2017: a systematic analysis for the Global Burden of Disease Study 2017. *Lancet* 2020;395(10225):709-33.
2. Jager KJ, Fraser SDS. The ascending rank of chronic kidney disease in the global burden of disease study. *Nephrology Dialysis Transplantation* 2017;32(suppl_2):ii121-ii28.
3. Schoolwerth AC, Engelgau MM, Hostetter TH, et al. Chronic kidney disease: a public health problem that needs a public health action plan. *Preventing Chronic Disease* 2006;3(2):A57.
4. Kidney Disease: Improving Global Outcomes (KDIGO) CKD Work Group. KDIGO 2012 Clinical Practice Guideline for the Evaluation and Management of Chronic Kidney Disease. *Kidney inter.*, Suppl 2013; 3: 1–150.
5. Kerr M, Bray B, Medcalf J, et al. Estimating the financial cost of chronic kidney disease to the NHS in England. *Nephrol Dial Transplant* 2012;27 Suppl 3:iii73-80.
6. UK Renal Registry (2020) UK Renal Registry 22nd Annual Report – data to 31/12/2018, Bristol, UK. 2020. https://ukkidney.org/audit-research/annual-report/22nd-annual-report-data-31122018. Accessed 27 Jan 2022.
7. Kim LG, Cleary F, Wheeler DC, et al. How do primary care doctors in England and Wales code and manage people with chronic kidney disease? Results from the National Chronic Kidney Disease Audit. *Nephrology Dialysis Transplantation* 2017;33(8):1373-79.
8. Nitsch D, Caplin B, Hull S, et al on behalf of the National CKD Audit and Quality Improvement Programme in Primary Care. First National CKD Audit Report. 2017. https://www.lshtm.ac.uk/files/ckd_audit_report.pdf. Accessed 28 Jul 2021.
9. NHS Digital. SNOMED CT. https://digital.nhs.uk/services/terminology-and-classifications/snomed-ct (2021). Accessed 27 Jan 2022.

For peer review only - http://bmjopen.bmj.com/site/about/guidelines.xhtml

10. National Institute for Health and Care Excellence. Chronic Kidney Disease in Adults: Assessment and Management (NICE Guideline CG182). 2014. https://www.nice.org.uk/guidance/cg182. Accessed 28 Jul 2021.

11. National Institute for Health and Care Excellence. NICE Quality and Outcomes Framework indicator. https://www.nice.org.uk/standards-and-indicators/qofindicators. Accessed 27 Jan 2022.

12. Mudie K, Cleary F, Caplin B, et al on behalf of the National CKD Audit and Quality Improvement Programme in Primary Care. Second National CKD Audit Report. 2017. https://www.lshtm.ac.uk/media/9951. Accessed 28 Jul 2021.

13. Capurro D, Coghlan S, Pires DEV. Preventing Digital Overdiagnosis. *JAMA* 2022;327(6):525-6.

14. London School of Hygiene and Tropical Medicine. National CKD Audit Steering Committee. https://www.lshtm.ac.uk/research/centres-projects-groups/ckdaudit#steering-committee. Accessed 27 Jan 2022.

15. NHS Digital. Read Codes. https://digital.nhs.uk/services/terminology-and-classifications/read-codes (2020). Accessed 27 Jan 2022.

16. Molokhia M, Okoli GN, Redmond P, et al. Uncoded chronic kidney disease in primary care: a cross-sectional study of inequalities and cardiovascular disease risk management. *British Journal of General Practice* 2020;70(700):e785-e92.

17. Hull SA, Rajabzadeh V, Thomas N, et al. Improving coding and primary care management for patients with chronic kidney disease: an observational controlled study in East London. *British Journal of General Practice* 2019;69(684):e454-e61.

18. Olaitan A, Ashman N, Homer K, et al. Predictors of late presentation to renal dialysis: a cohort study of linked primary and secondary care records in East London. *BMJ Open* 2019;9(6):e028431.

For peer review only - http://bmjopen.bmj.com/site/about/guidelines.xhtml

19. Xie X, Liu Y, Perkovic V, et al. Renin-Angiotensin System Inhibitors and Kidney and Cardiovascular Outcomes in Patients With CKD: A Bayesian Network Meta-analysis of Randomized Clinical Trials. *Am J Kidney Dis* 2016;67(5):728-41.

20. Herrington WG, Emberson J, Mihaylova B, et al. Impact of renal function on the effects of LDL cholesterol lowering with statin-based regimens: a meta-analysis of individual participant data from 28 randomised trials. *Lancet Diabetes Endocrinol* 2016;4(10):829-39.

21. Wright JT Jr, Williamson JD, Whelton PK, et al. A Randomized Trial of Intensive versus Standard Blood-Pressure Control. *New England Journal of Medicine* 2015;373(22):2103-16.

For peer review only

FIGURE LEGENGS

Figure 1. **Flow chart of selection of study population (confirmed CKD in last 2 years)**

Figure 2. HR curves for all outcomes according to practice percent coded CKD.
Analysis includes all patients with confirmed CKD. Primary exposure is (continuous) practice percent coded CKD, with median practice coding (74% coded) as the reference group. Model 1 adjusts for practice risk profile characteristics: mean age, percent male, median rank of index of multiple deprivation, diabetes prevalence, hypertension prevalence, CVD prevalence. Model 2 adjusts for model 1 variables and additionally for practice characteristics of CKD population and testing behaviours: percent of CKD cases at stages 3b-5, percent of CKD patients admitted for COPD in last 3 years, percent of CKD patients admitted for cancer in last 3 years, percent GFR test in last year in diabetes, percent GFR test in last year in CKD, percent of adult population with CKD. Labels S1 to S6 descriptively indicate sextiles of practice percent coded CKD, with each sextile representing one sixth of all practices.

For peer review only

For peer review only - http://bmjopen.bmj.com/site/about/guidelines.xhtml

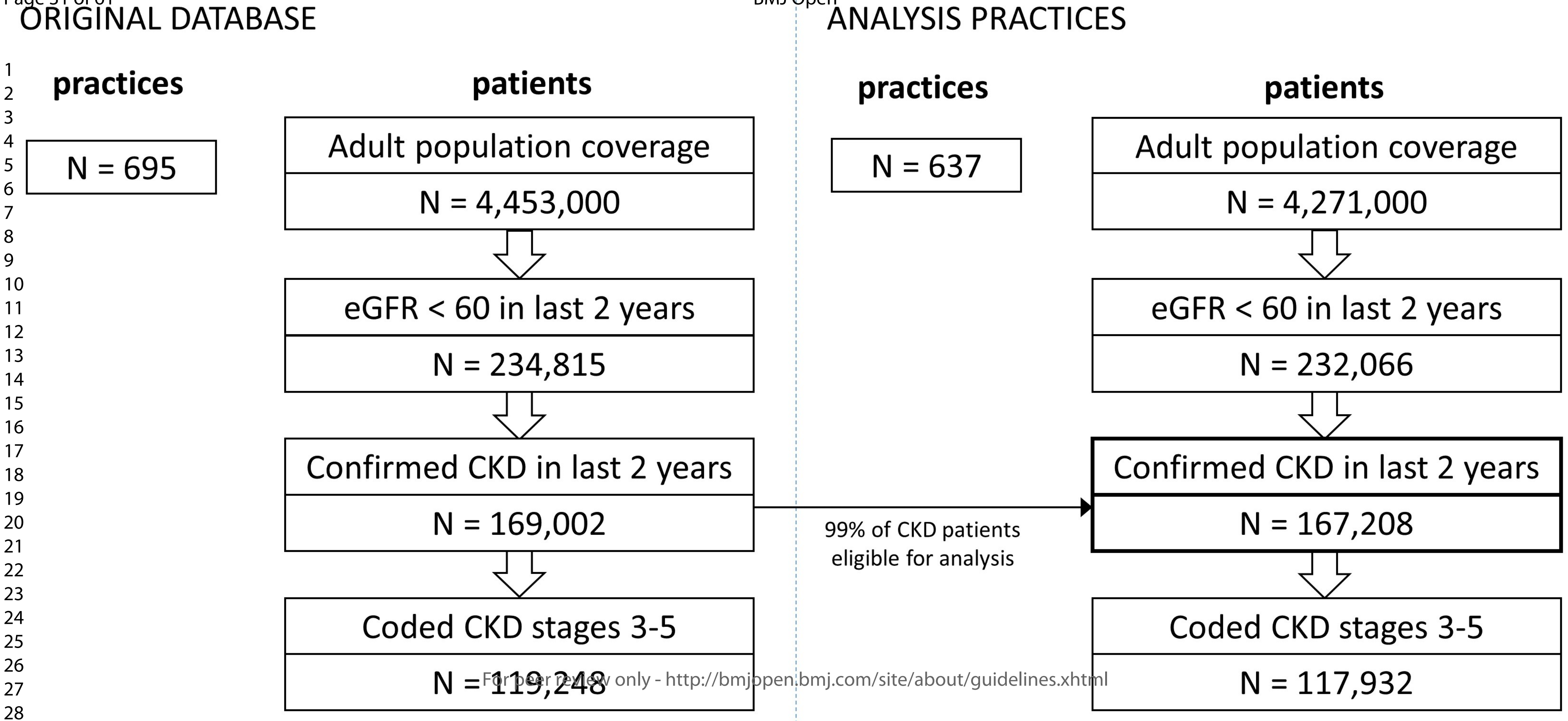

For peer review only - http://bmjopen.bmj.com/site/about/guidelines.xhtml

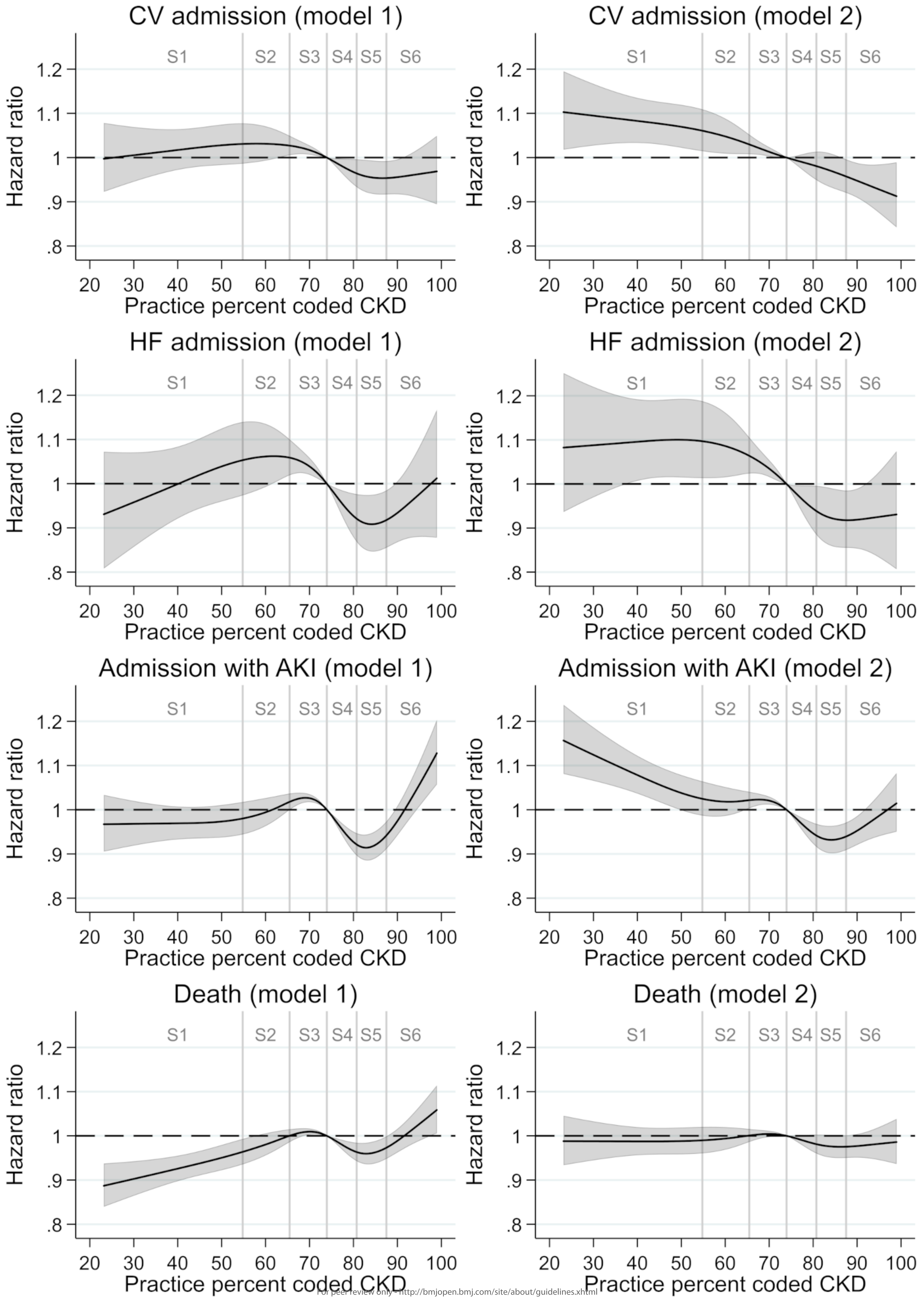
CV admission (model 1)
CV admission (model 2)
HF admission (model 1)
HF admission (model 2)
Admission with AKI (model 1)
Admission with AKI (model 2)
Death (model 1)
Death (model 2)
S1
S2
S3
S4
S5
S6
Hazard ratio
1.2
1.1
1
.9
.8
20
30
40
50
60
70
80
90
100
Practice percent coded CKD

SUPPLEMENTARY MATERIALS

Supplementary Table 1. Outcome definitions

Outcome	Definition	Details	ICD-10 codes
AKI	AKI at admission	Any diagnosis (HES) of AKI recorded in the first episode of care	N17
HF	Admission for HF	A primary diagnosis (HES) of HF recorded in the first episode of care	I50, I11.0, I13, I97.1
CV event	Admission for CV event	A primary diagnosis (HES) of HF, CHD, stroke/TIA, PAD or AAA recorded in the first episode of care	HF (I50, I11.0, I13, I97.1)<br>CHD (I20-I25, I51.6)<br>Stroke/TIA (G45-G46)<br>PAD (I79.0, I79.2, I73.8, I73.9, I74.3, I74.4, I74.5, I70.2)<br>AAA (I71, I74.0)<br>Cerebrovascular disease (I60-I69)
All-cause mortality	All-cause mortality	Any death (ONS)	N/A

Supplementary Table 2. Percentage of practices and patients eligible for analysis after application of sample size eligibility criteria (minimum of 50 CKD cases) and comparison of coding rates in the original database and analysis database, in the CKD population and in subgroups

Population	Total practices	Total patients	Total patients coded	Eligible practices	Eligible patients	Eligible patients coded
All CKD	695	169,002	119,248 (70.6%)	637 (91.7%)	167,208 (98.9%)	117,932 (70.5%)
CKD stage 3a	695	106,981	66,514 (62.2%)	580 (83.5%)	103,615 (96.9%)	64,398 (62.2%)
CKD stages 3b-5	695	62,021	52,734 (85.0%)	477 (68.6%)	56,122 (90.5%)	47,700 (84.5%)
Diabetes	695	42,063	32,099 (76.3%)	362 (52.1%)	33,065 (78.6%)	25,273 (76.4%)
No diabetes	695	126,939	87,149 (68.7%)	605 (87.1%)	124,364 (98.0%)	85,324 (68.6%)

For peer review only - http://bmjopen.bmj.com/site/about/guidelines.xhtml

Supplementary Information 1. Methods for estimating the percentage of CV and HF hospitalisation events that are preventable among CKD patients in practices coding 55% of CKD cases if practice coding improved to 88% (attributable fraction for first events)

In order to estimate the percentage of first hospitalisation events that could be prevented over a period of 3.8 years (median follow-up duration) among patients in practices coding 55% of CKD cases (13th practice coding percentile, lower boundary of sextile 2), if such practices instead coded 88% of CKD cases (83rd practice coding percentile, upper boundary of sextile 5), we adopted the Austin method in [1], which we adapted for use in STATA statistical software. We make an assumption that after adjustment for model covariates, the difference in expected event rates between practice coding groups estimated using the methods listed below is caused by practice coding performance. We followed the following steps to estimate the percentage of first events attributable to lower practice coding (55%), when compared to higher practice coding (88%), for both CV and HF hospitalisations:

1. Fit the fully adjusted Cox regression model for time to first event with 5-knot spline for practice percent coded CKD and other practice covariates (model 2), first centring all continuous covariates to generate a sensible baseline group
2. Estimate the baseline survival function [2] using: "predict s, basesurv", sort data by analysis time and extract the baseline survival probability at t = 3.8 years (median follow-up)
3. Predict the estimated survival probability at t = 3.8 years for every patient in database assuming 54.8% practice percent coded, based on true values of all covariates except with practice percent coded forced to 54.8%, as follows:
    a. Recode data to set value of practice coding variable to 54.8% coded in all patients. (Spline variable values recoded accordingly.)
    b. Use equation $\hat{S}_i(t) = \hat{S}_0(t)^{\exp(\boldsymbol{x}_i\widehat{\boldsymbol{\beta}})}$ [3] to estimate individual survival probabilities $\hat{S}_i(t)$ at time t = 3.8 years, where $\hat{S}_0(t)$ is the estimated baseline survival at time t and $\boldsymbol{x}_i\widehat{\boldsymbol{\beta}}$ is the individual prediction of the linear predictor evaluated at individual true covariate values $\boldsymbol{x}_i$ (but specified practice percent coded of 54.8%) based on coefficient estimates $\widehat{\boldsymbol{\beta}}$
4. Repeat step 3 for practice percent coded forced to 87.5% coded, to obtain individual predicted survival probabilities at t = 3.8 years assuming 87.5% practice percent coded
5. Estimate the expected number of first events occurring over 3.8 years if all practices coded at 54.8% (with other practice characteristics unchanged) by taking the sum of individual probabilities of an event (1 - $\hat{S}_i(t = 3.8)$, using results from (3b)). Repeat for assumption of all practices coding at 87.5% by taking the sum of individual probabilities of an event (1 - $\hat{S}_i(t = 3.8)$, from (4b)).
6. Using results from step 5, compute the percentage of events preventable among individuals in practices coding 54.8% of CKD cases if practices instead coded 87.5% of CKD cases (attributable fraction) as:

$$\%\ events\ preventable\ in\ practices\ coding\ 54.8\%\ of\ CKD\ cases$$

$$= \frac{expected\ events\ assuming\ 54.8\%\ coding - expected\ events\ assuming\ 87.5\%\ coding}{expected\ events\ assuming\ 54.8\%\ coding} \times 100\%$$

For peer review only - http://bmjopen.bmj.com/site/about/guidelines.xhtml

References:

1. Zhang Z, Ambrogi F, Bokov AF, et al. Estimate risk difference and number needed to treat in survival analysis. *Ann Transl Med* 2018;6(7):120.
2. StataCorp. STATA Survival Analysis Reference Manual Release 17. 2021. StataCorp LLC. https://www.stata.com/manuals/st.pdf. Accessed 17 Feb 2022.
3. Rodriguez, G. Approaches to Survival Modelling. In: Lecture Notes on Generalized Linear Models. Princeton University. 2007. https://data.princeton.edu/wws509/notes/c7.pdf. Accessed 17 Feb 2022

For peer review only

Supplementary Figure 1. Distribution of practice CKD coding performance in the CKD analysis population and in subgroups, with red lines depicting sextile boundaries

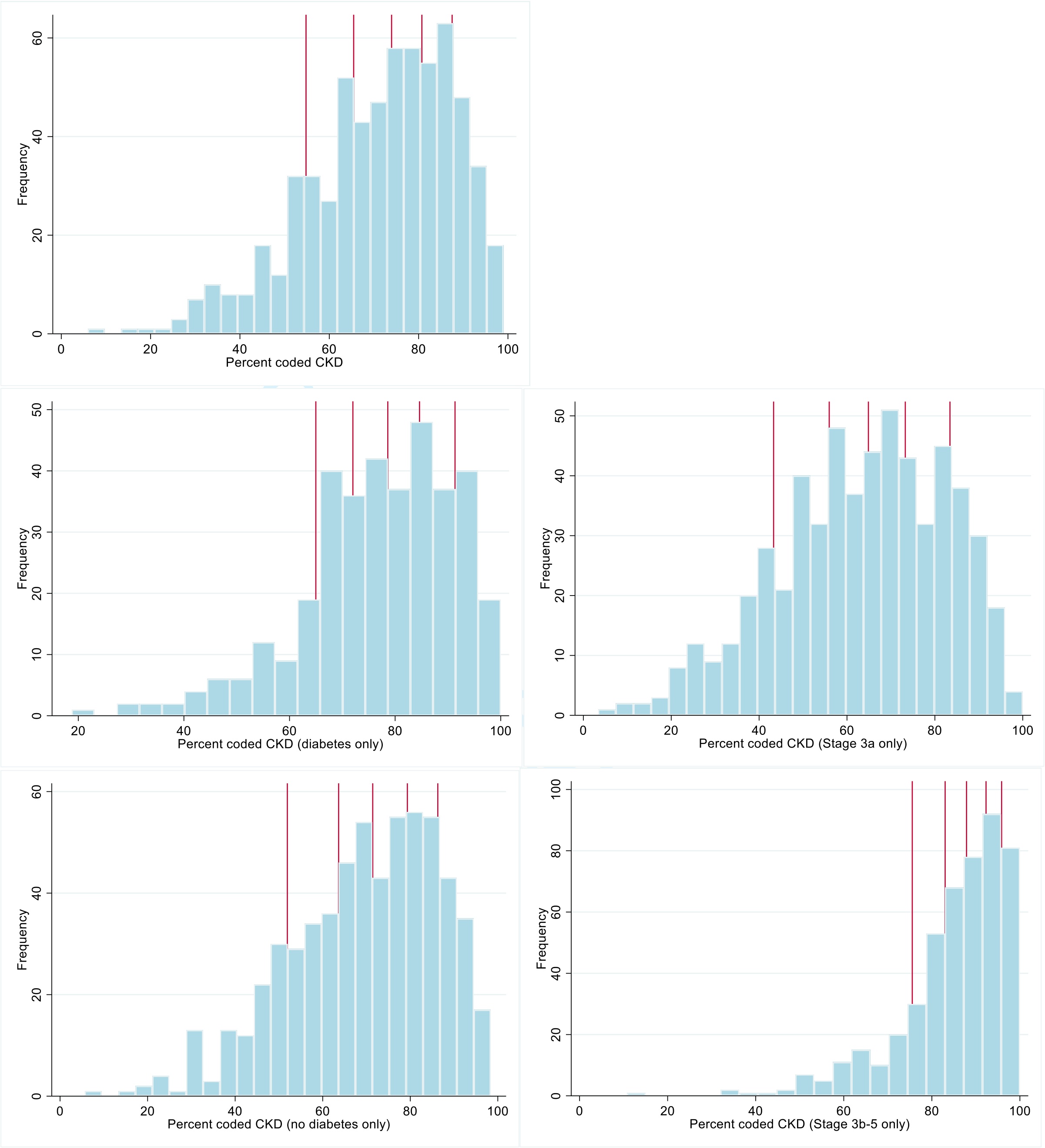

Supplementary Figure 2. Event rates per 100 patient years and 95% confidence intervals, by practice coding sextile in **all CKD** patients (including **recurring events**)

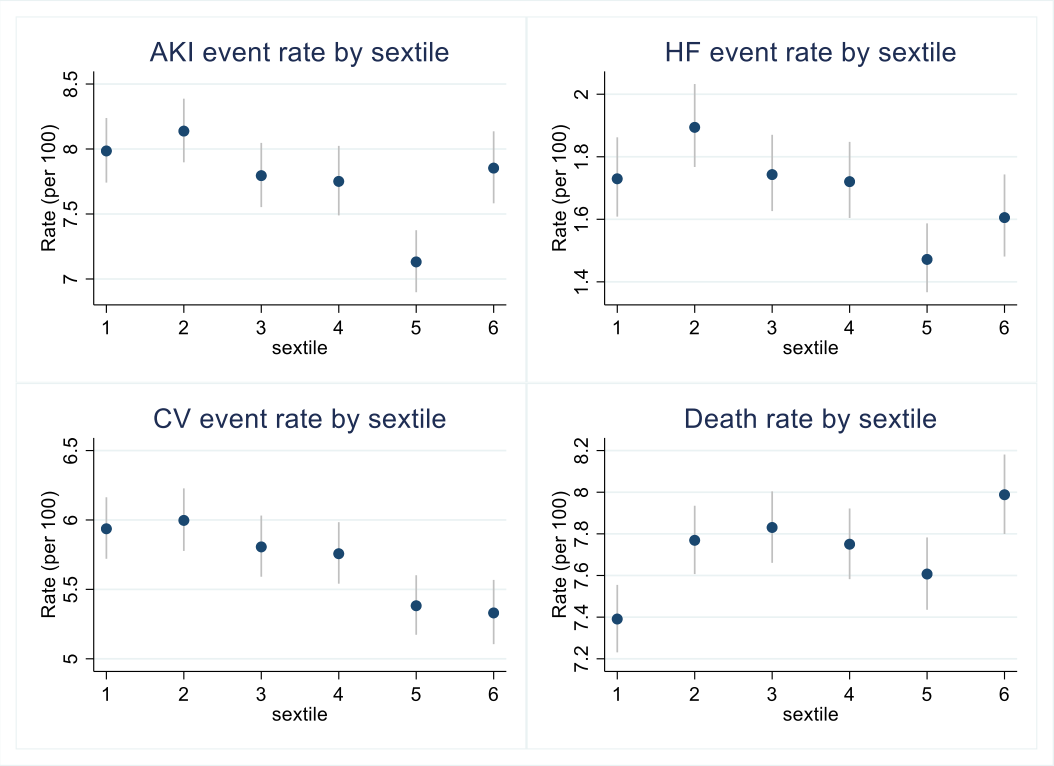

Supplementary Figure 3. Event rates per 100 patient years and 95% confidence intervals, by practice coding sextile in **all CKD** patients (**first events** only)

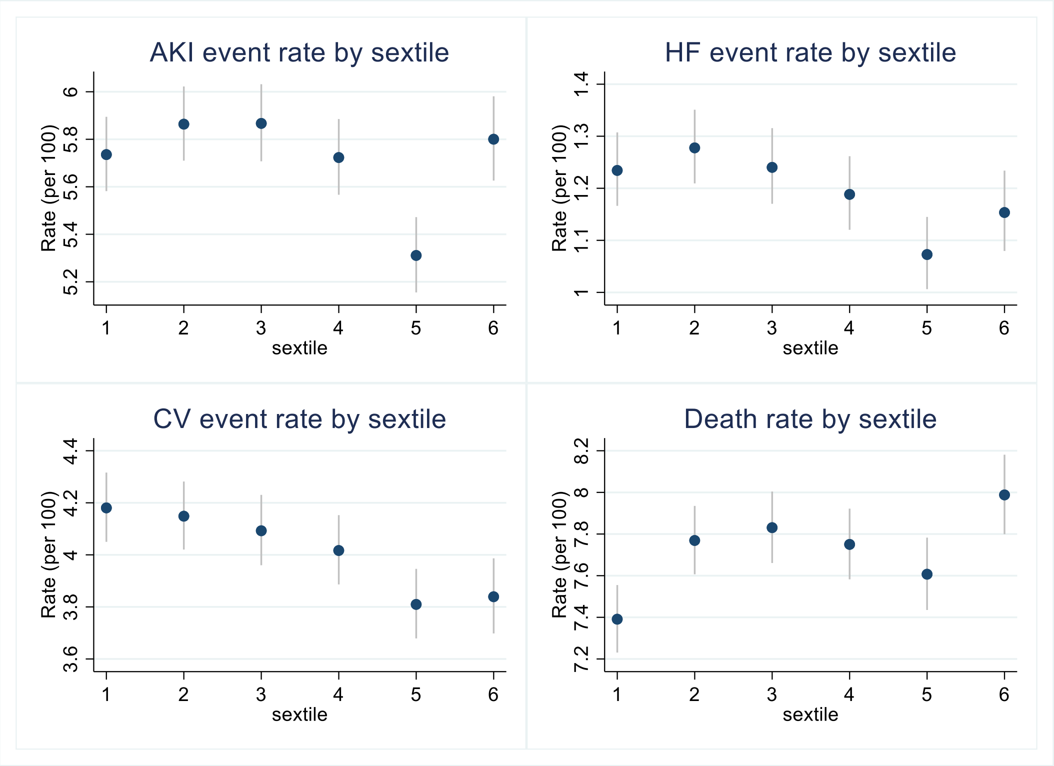

For peer review only - http://bmjopen.bmj.com/site/about/guidelines.xhtml

Supplementary Table 3. Event rates (including recurring events) per 100 patient years and 95% confidence intervals, by practice coding sextile

**Practice coding sextile (percent coded CKD)**	**S1 (<54.8%)**	**S2 (54.8% - 65.5%)**	**S3 (65.5% - 73.9%)**	**S4 (73.9% - 80.7%)**	**S5 (80.7% - 87.5%)**	**S6 (≥87.5%)**
CV event	N_E = 5766<br>5.94<br>(5.72, 6.16)	N_E = 5980<br>6.00<br>(5.78, 6.23)	N_E = 5326<br>5.81<br>(5.59, 6.03)	N_E = 5340<br>5.76<br>(5.54, 5.98)	N_E = 4677<br>5.38<br>(5.17, 5.60)	N_E = 4037<br>5.33<br>(5.11, 5.57)
HF	N_E = 1680<br>1.73<br>(1.61, 1.86)	N_E = 1889<br>1.89<br>(1.77, 2.03)	N_E = 1599<br>1.74<br>(1.63, 1.87)	N_E = 1596<br>1.72<br>(1.60, 1.85)	N_E = 1279<br>1.47<br>(1.37, 1.59)	N_E = 1216<br>1.61<br>(1.48, 1.74)
AKI	N_E = 7755<br>7.99<br>(7.74, 8.24)	N_E = 8114<br>8.14<br>(7.90, 8.39)	N_E = 7150<br>7.80<br>(7.55, 8.05)	N_E = 7189<br>7.75<br>(7.49, 8.02)	N_E = 6197<br>7.13<br>(6.90, 7.38)	N_E = 5947<br>7.85<br>(7.58, 8.14)
All-cause mortality	N_E = 7985<br>7.39<br>(7.23, 7.55)	N_E = 8612<br>7.77<br>(7.61, 7.93)	N_E = 7994<br>7.83<br>(7.66, 8.00)	N_E = 8014<br>7.75<br>(7.58, 7.92)	N_E = 7362<br>7.61<br>(7.43, 7.78)	N_E = 6740<br>7.99<br>(7.80, 8.18)

N_E = number of events

Supplementary Table 4 Event rates (first events only) per 100 patient years and 95% confidence intervals, by practice coding sextile

**Practice coding sextile (percent coded CKD)**	**S1 (<54.8%)**	**S2 (54.8% - 65.5%)**	**S3 (65.5% - 73.9%)**	**S4 (73.9% - 80.7%)**	**S5 (80.7% - 87.5%)**	**S6 (≥87.5%)**
CV event	N_E = 3834<br>4.18<br>(4.05, 4.32)	N_E = 3911<br>4.15<br>(4.02, 4.28)	N_E = 3553<br>4.09<br>(3.96, 4.23)	N_E = 3532<br>4.02<br>(3.89, 4.15)	N_E = 3144<br>3.81<br>(3.68, 3.95)	N_E = 2758<br>3.84<br>(3.70, 3.96)
HF	N_E = 1183<br>1.23<br>(1.17, 1.31)	N_E = 1257<br>1.28<br>(1.21, 1.35)	N_E = 1123<br>1.24<br>(1.17, 1.31)	N_E = 1089<br>1.19<br>(1.12, 1.26)	N_E = 922<br>1.07<br>(1.01, 1.14)	N_E = 863<br>1.15<br>(1.08, 1.23)
AKI	N_E = 5228<br>5.74<br>(5.58, 5.89)	N_E = 5480<br>5.86<br>(5.71, 6.02)	N_E = 5055<br>5.87<br>(5.71, 6.03)	N_E = 4993<br>5.72<br>(5.57, 5.88)	N_E = 4359<br>5.31<br>(5.16, 5.47)	N_E = 4135<br>5.80<br>(5.63, 5.98)
All-cause mortality	N_E = 7985<br>7.39<br>(7.23, 7.55)	N_E = 8612<br>7.77<br>(7.61, 7.93)	N_E = 7994<br>7.83<br>(7.66, 8.00)	N_E = 8014<br>7.75<br>(7.58, 7.92)	N_E = 7362<br>7.61<br>(7.43, 7.78)	N_E = 6740<br>7.99<br>(7.80, 8.18)

N_E = number of events

For peer review only - http://bmjopen.bmj.com/site/about/guidelines.xhtml

Supplementary Information 2. Methods for sequentially adjusted Cox regression analyses shown in supplementary analyses

Main Cox regression analyses, models 1 and 2, adjusted for practice characteristics are described in the main methods (repeated here for completeness) with results for all studied outcomes shown in the main results. Supplementary figures 4-23 show additional sequential adjustments, demonstrating the role of confounding as different variables were incorporated in analyses, as follows:

- Crude analysis: unadjusted
- Model 1: adjusted for ***practice characteristics reflecting practice risk profile*** (mean age, percent male, median rank of IMD, diabetes prevalence, hypertension prevalence, CVD prevalence)
- Model 1.5: adjusted for ***practice characteristics reflecting practice risk profile*** (model 1 variables), as well as ***practice characteristics of the detected CKD population*** (percent of CKD cases stages 3b-5 ["CKD severity"]), percent admitted for COPD in last 3 years, percent admitted for cancer in last 3 years)
- Model 2: adjusted for ***practice characteristics relating to overall practice risk profile*** and ***of the detected CKD population*** (model 1.5 variables), as well as ***testing biases*** which may result in confounding due to different vintages (i.e. duration of underlying disease) (percent GFR test in last year in diabetes, percent GFR test in last year in CKD, percent of adult population with detected CKD)

As in main analyses, adjusted hazard ratios (HRs) for outcomes with 95% confidence intervals were plotted across the spectrum of practice CKD coding, compared to average practice CKD coding.

For peer review only

Supplementary Figure 4. Hazard ratio splines for time to first **CV event**, compared to median (73.9%) practice percent coded (sequential adjustments)

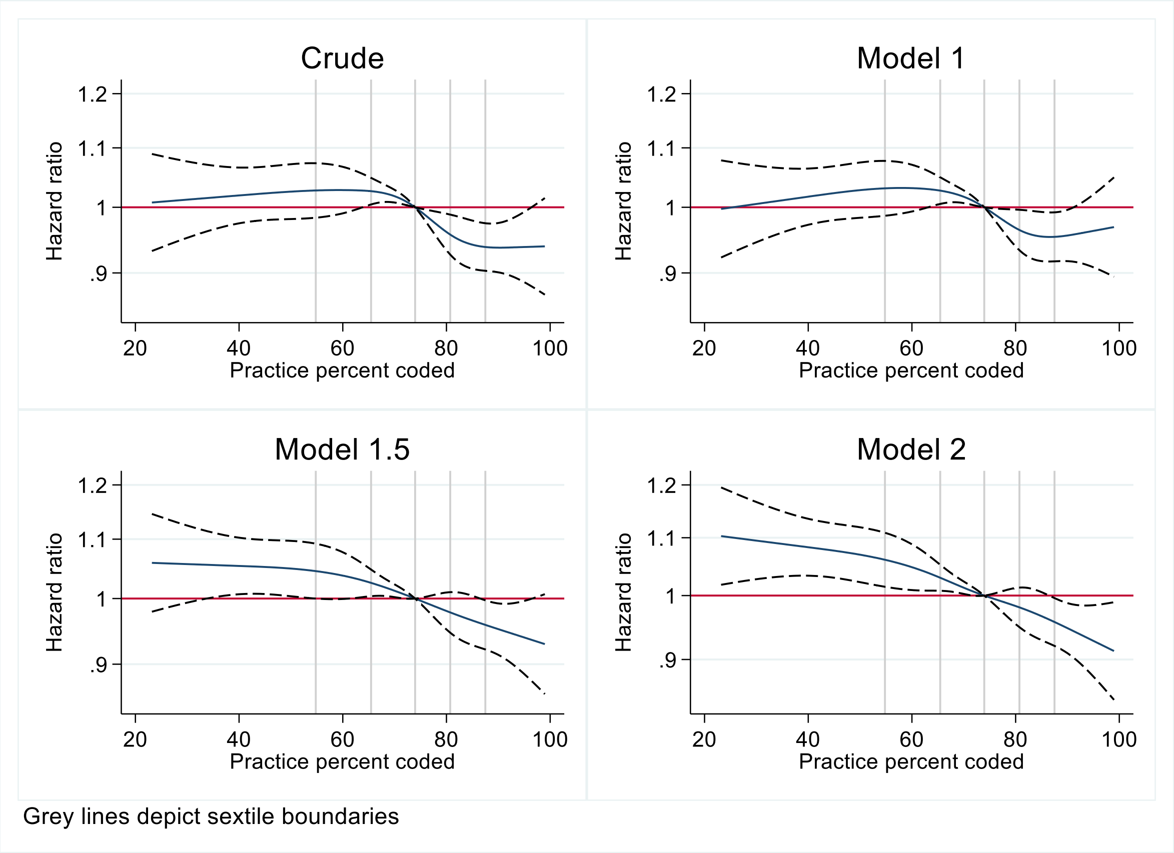

For peer review only

Supplementary Figure 5. Hazard ratio splines for time to first **CV event**, compared to median (64.9%) practice percent coded, in **CKD stage 3a only** (sequential adjustments)

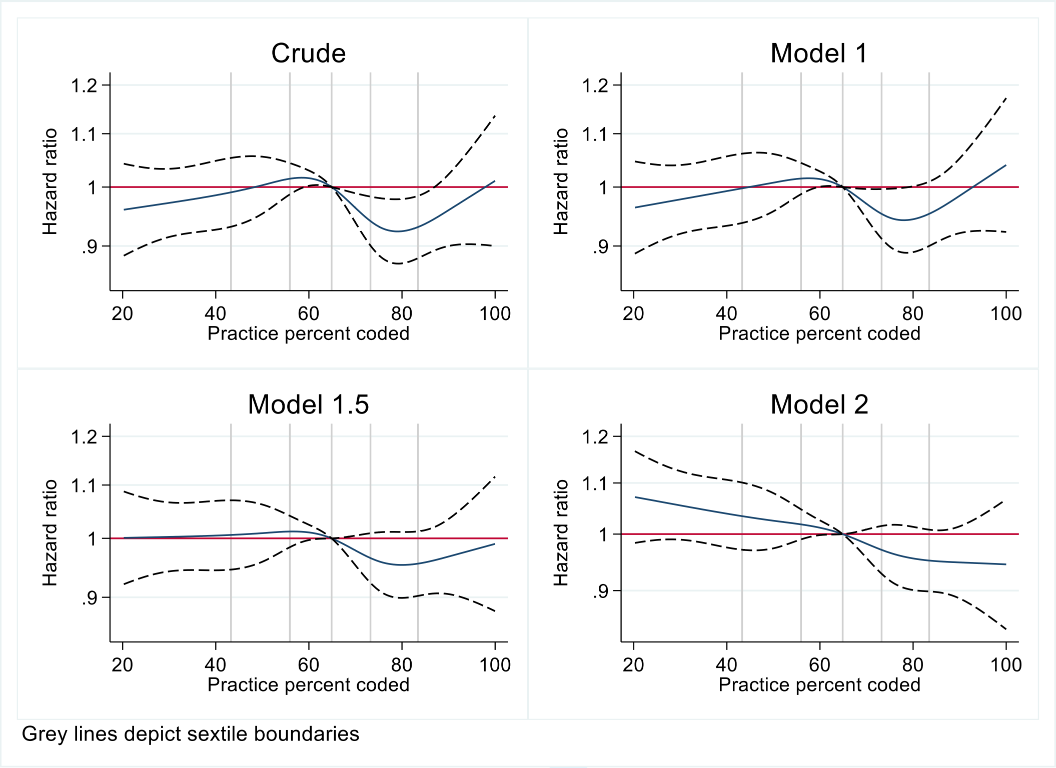

Supplementary Figure 6. Hazard ratio splines for time to first **CV event**, compared to median (87.9%) practice percent coded, in **CKD stages 3b-5 only** (sequential adjustments)

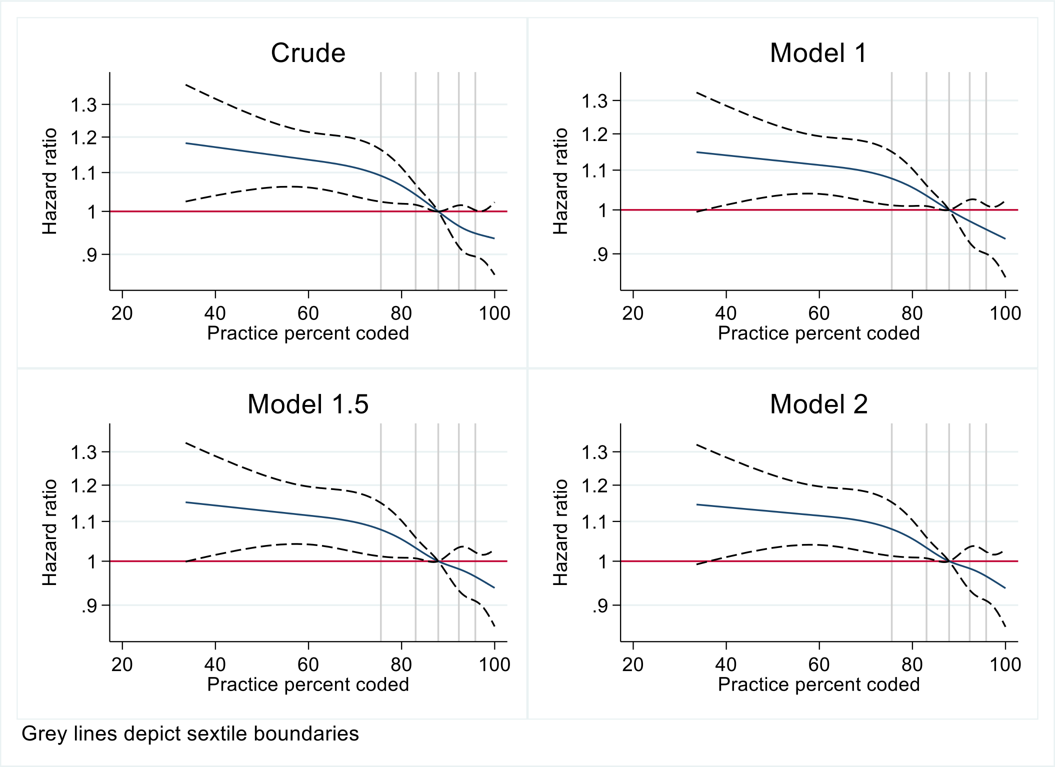

For peer review only - http://bmjopen.bmj.com/site/about/guidelines.xhtml

Supplementary Figure 7. Hazard ratio splines for time to first **CV event**, compared to median (78.6%) practice percent coded, in **diabetes only** (sequential adjustments)

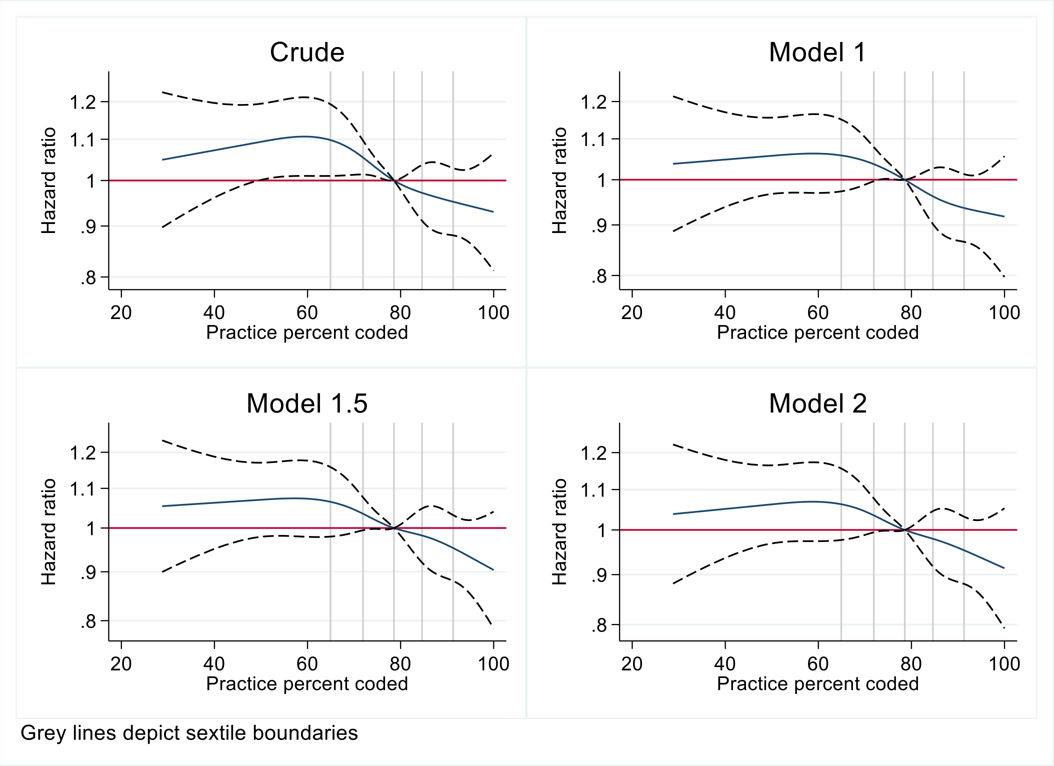

Supplementary Figure 8. Hazard ratio splines for time to first **CV event**, compared to median (71.4%) practice percent coded, in **no diabetes only** (sequential adjustments)

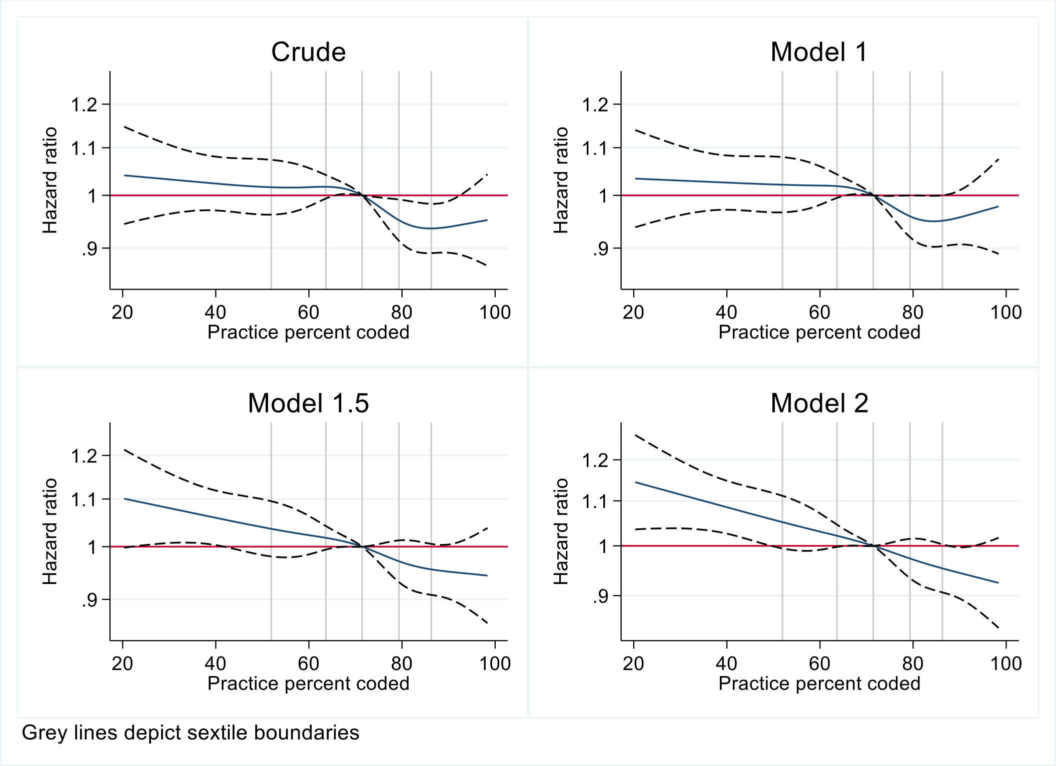

Supplementary Figure 9. Hazard ratio splines for time to first **HF** event, compared to median (73.9%) practice percent coded (sequential adjustments)

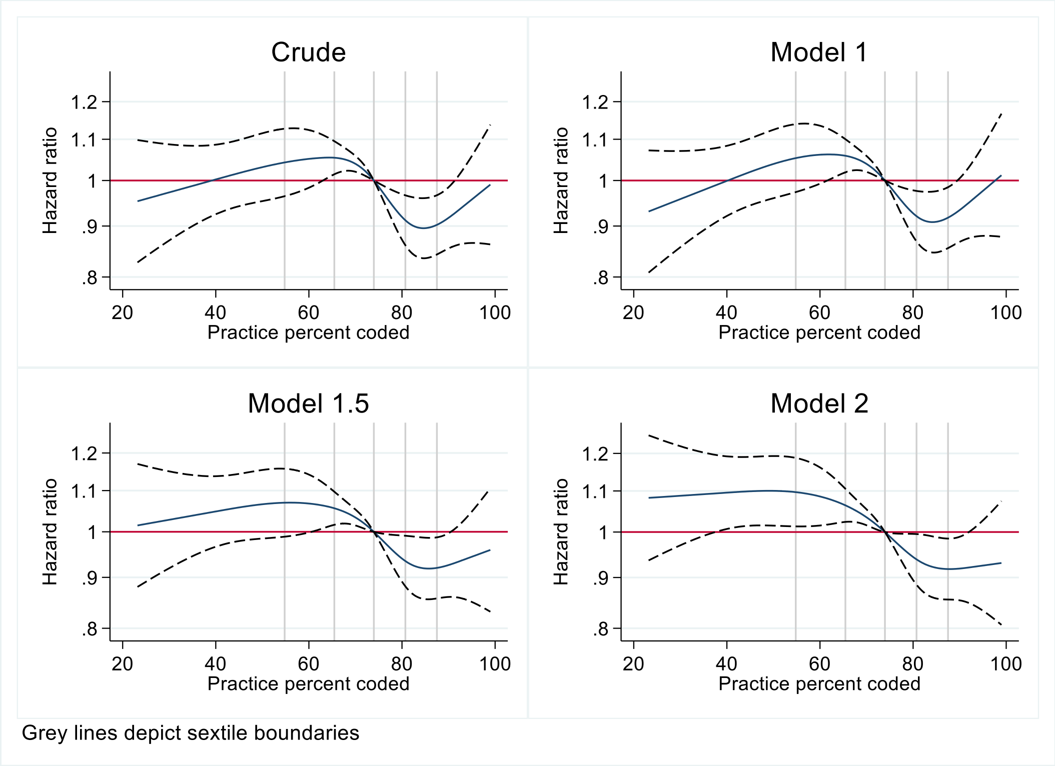

For peer review only - http://bmjopen.bmj.com/site/about/guidelines.xhtml

Supplementary Figure 10. Hazard ratio splines for time to first **HF** event, compared to median (64.9%) practice percent coded, in **CKD stage 3a only** (sequential adjustments)

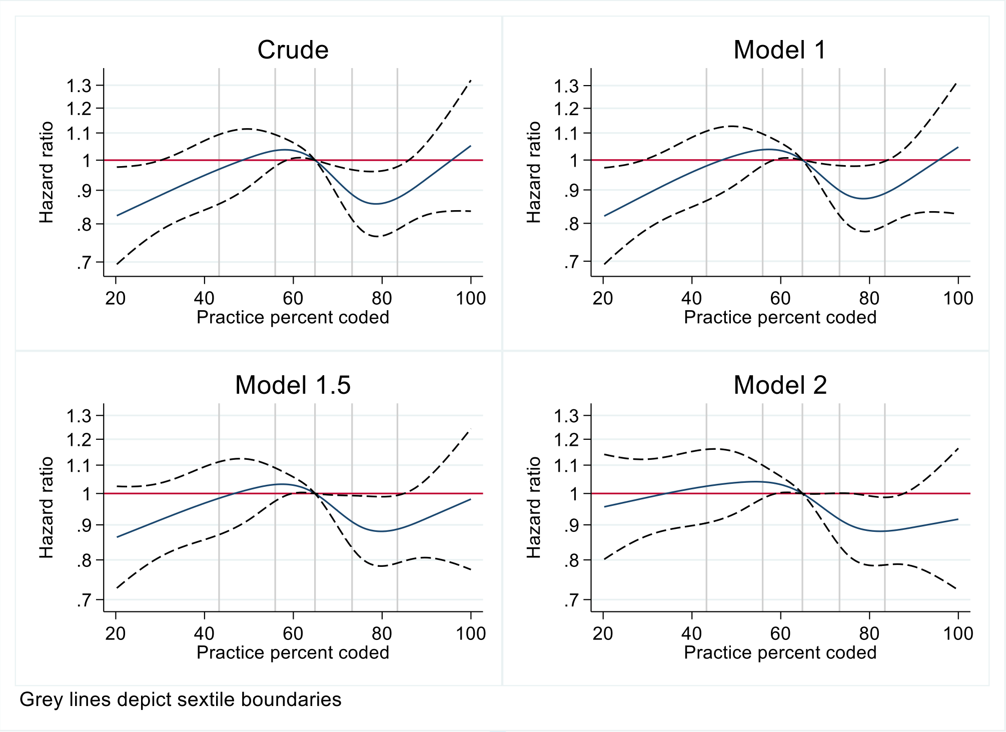

Supplementary Figure 11. Hazard ratio splines for time to first **HF** event, compared to median (87.9%) practice percent coded, in **CKD stages 3b-5** only (sequential adjustments)

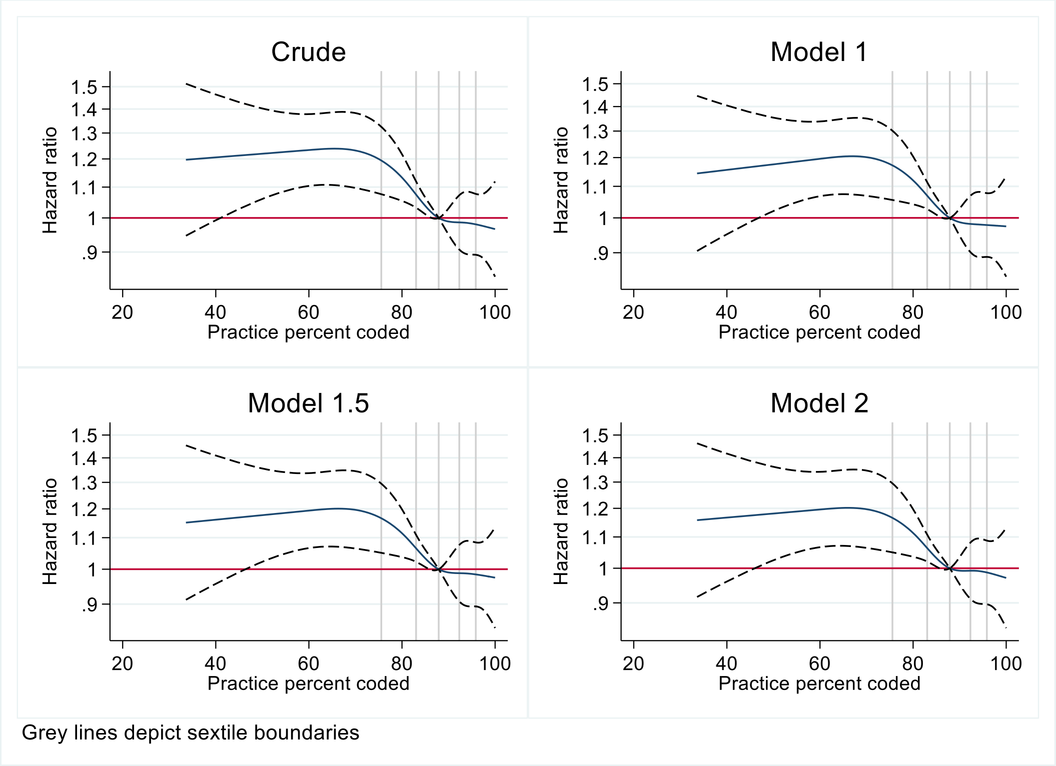

For peer review only - http://bmjopen.bmj.com/site/about/guidelines.xhtml

Supplementary Figure 12. Hazard ratio splines for time to first **HF** event, compared to median (78.6%) practice percent coded, in **diabetes only** (sequential adjustments)

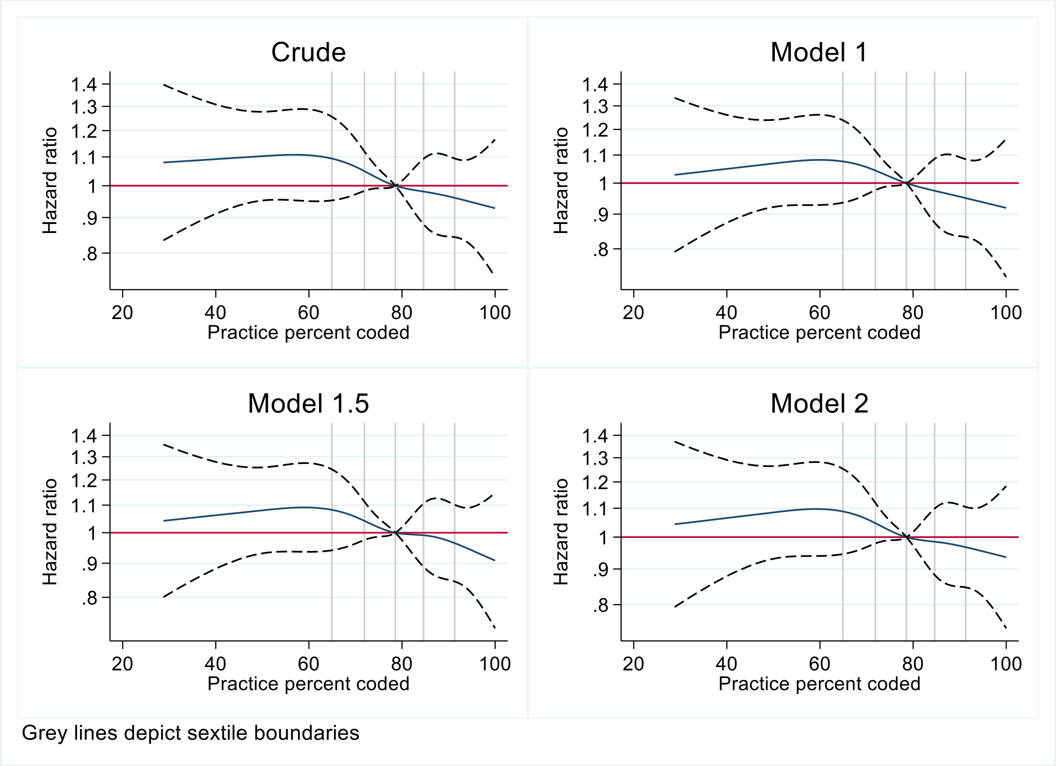

Supplementary Figure 13. Hazard ratio splines for time to first **HF** event, compared to median (71.4%) practice percent coded, in **no diabetes only** (sequential adjustments)

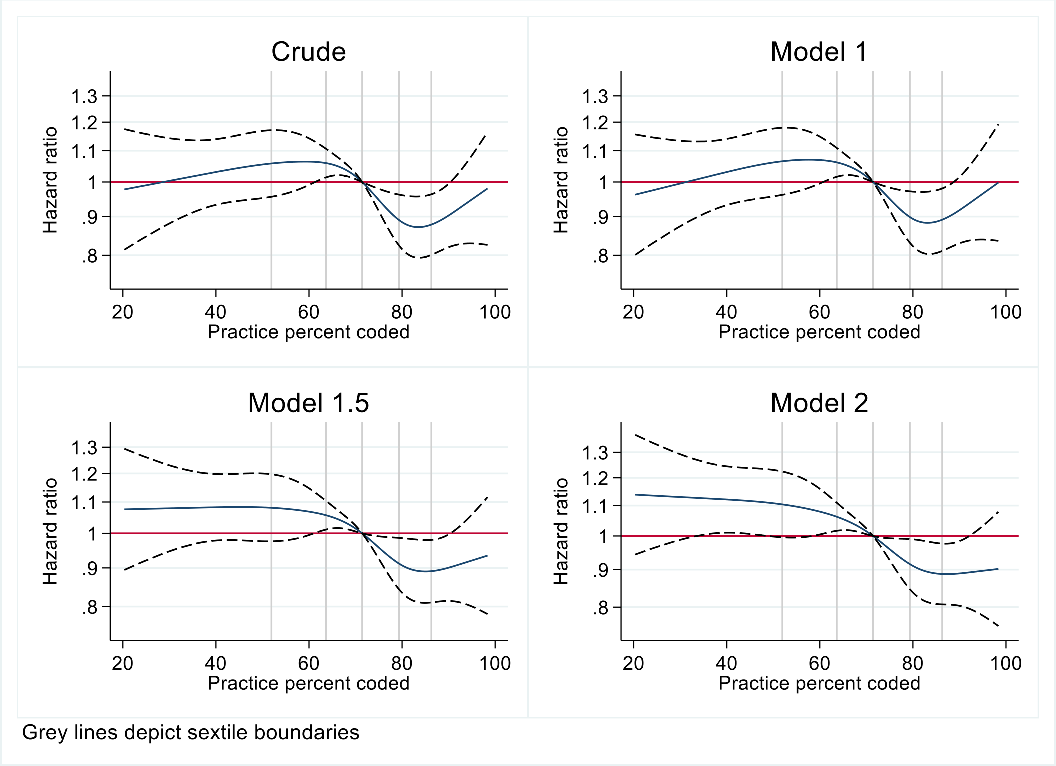

For peer review only - http://bmjopen.bmj.com/site/about/guidelines.xhtml

Supplementary Figure 14. Hazard ratio splines for time to first **AKI** event, compared to median (73.9%) practice percent coded (sequential adjustments)

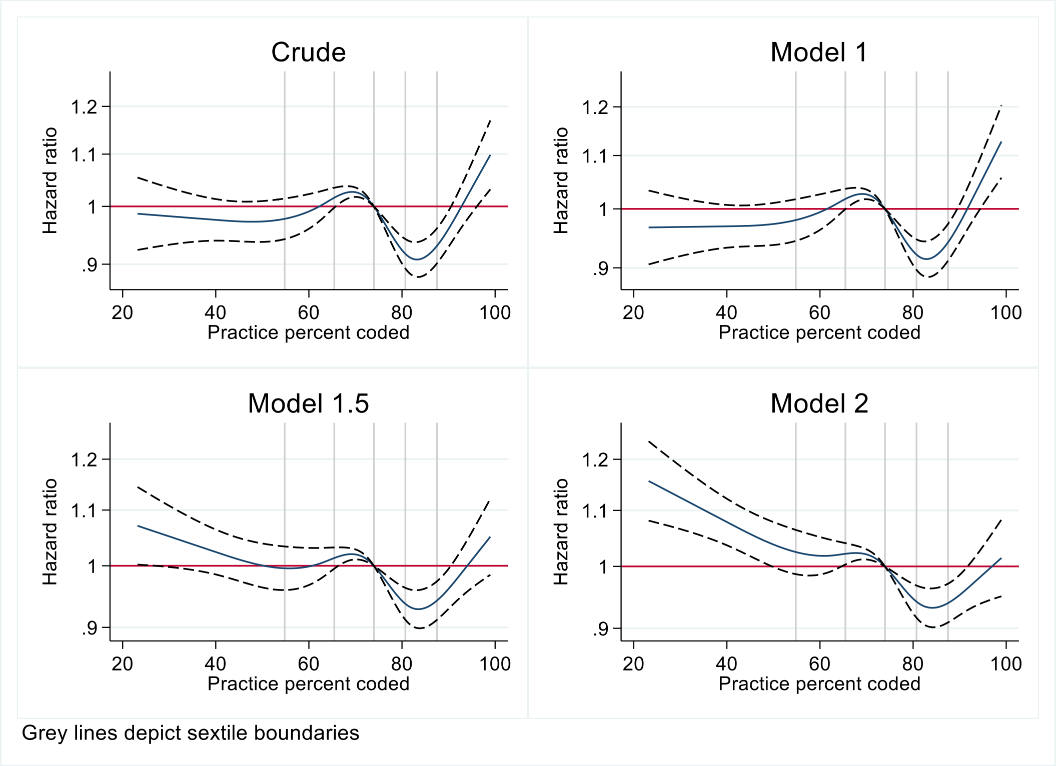

review only

For peer review only - http://bmjopen.bmj.com/site/about/guidelines.xhtml

Supplementary Figure 15 Hazard ratio splines for time to first **AKI** event, compared to median (64.9%) practice percent coded, in **CKD stage 3a only** (sequential adjustments)

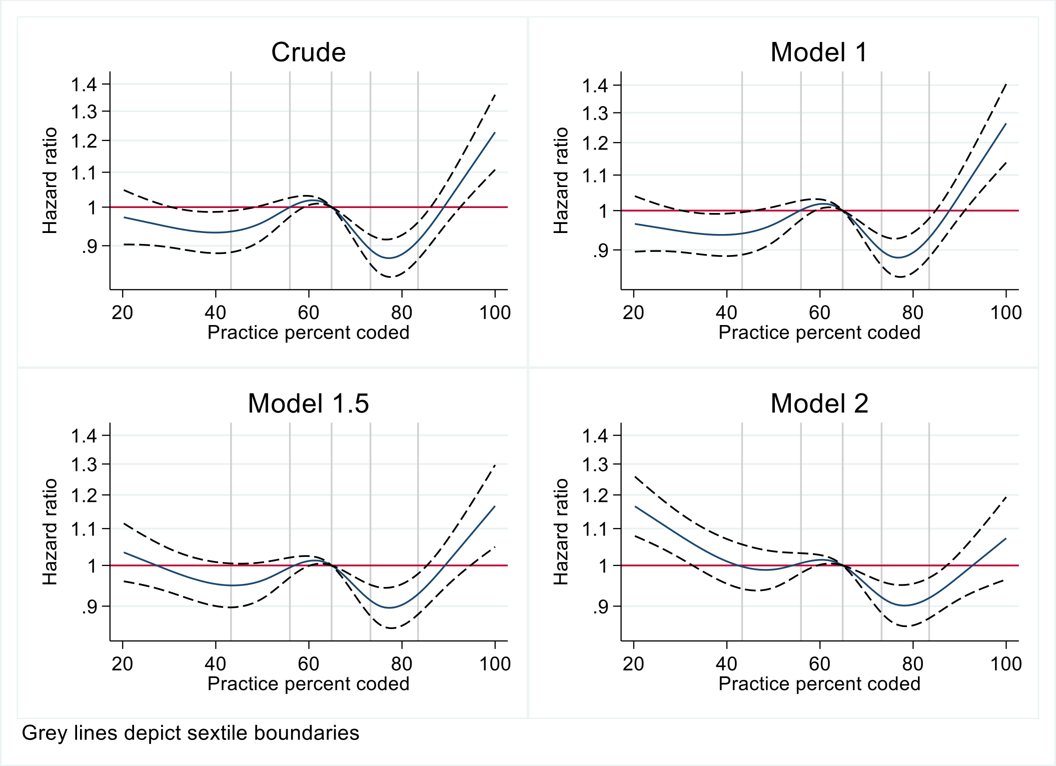

Supplementary Figure 16. Hazard ratio splines for time to first **AKI** event, compared to median (87.9%) practice percent coded, in **CKD stages 3b-5 only** (sequential adjustments)

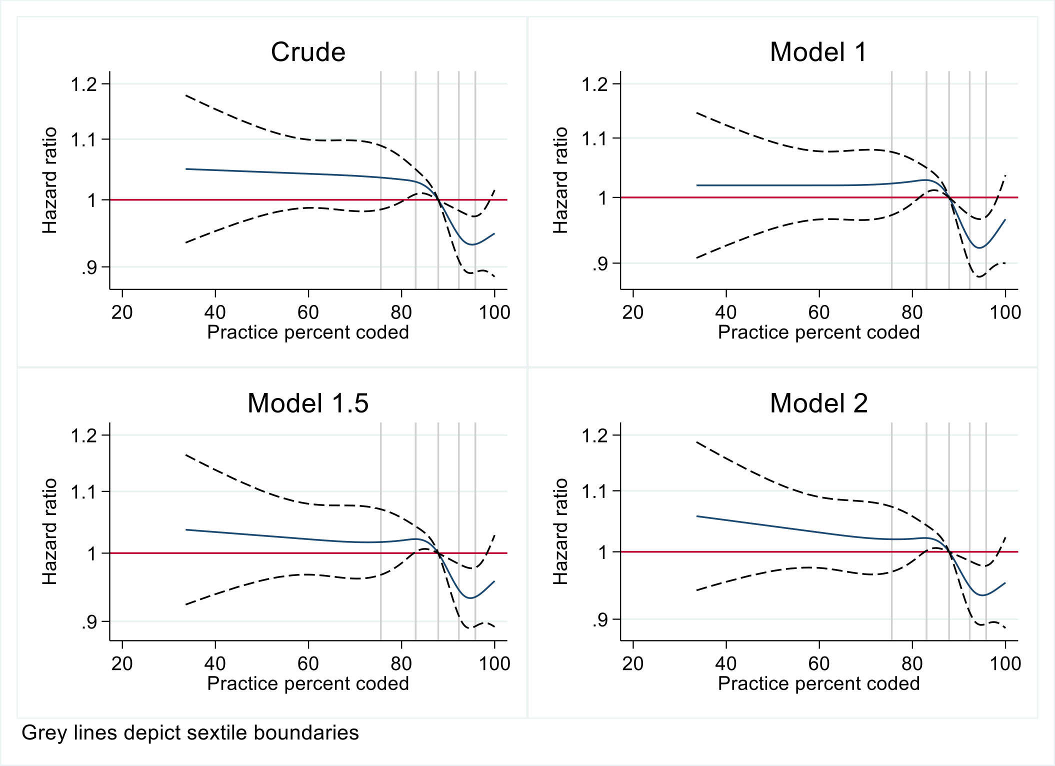

Supplementary Figure 17. Hazard ratio splines for time to first **AKI** event, compared to median (78.6%) practice percent coded, in **diabetes only** (sequential adjustments)

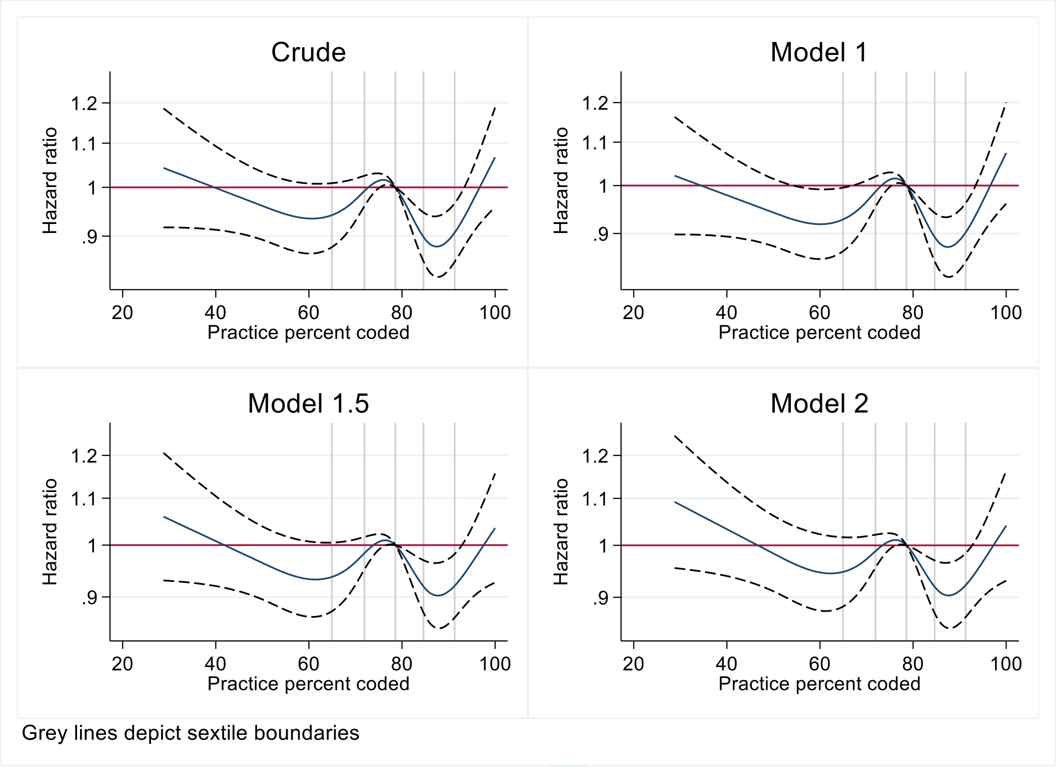

Supplementary Figure 18. Hazard ratio splines for time to first **AKI** event, compared to median (71.4%) practice percent coded, in **no diabetes only** (sequential adjustments)

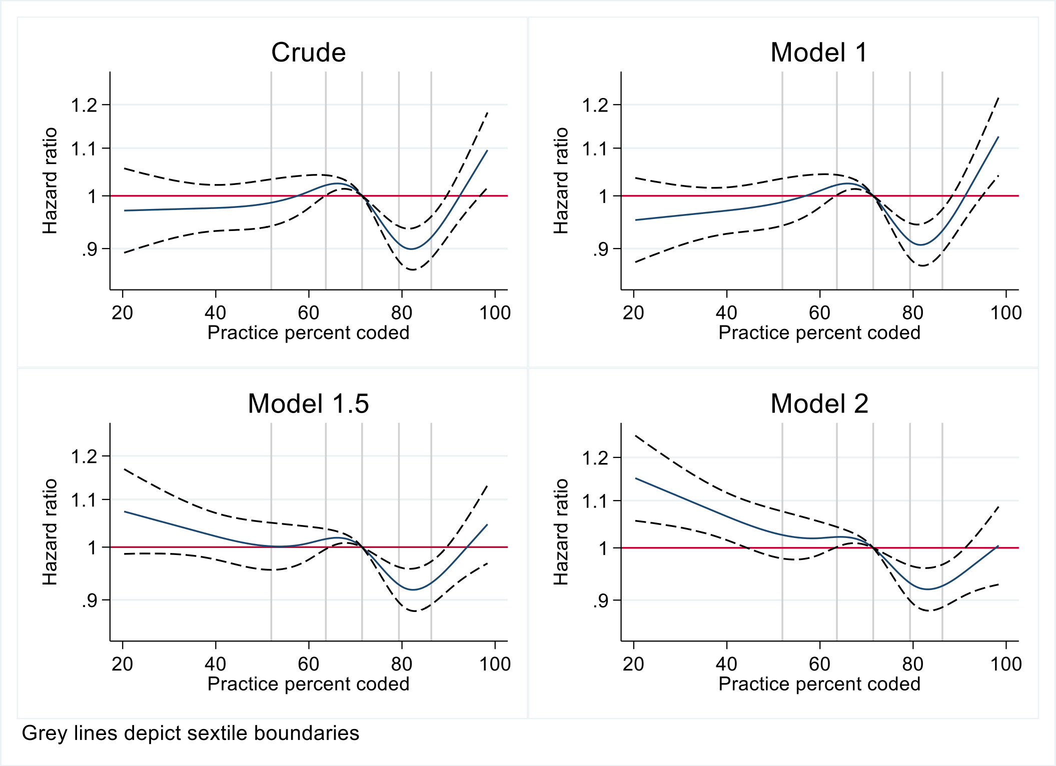

For peer review only - http://bmjopen.bmj.com/site/about/guidelines.xhtml

Supplementary Figure 19. Hazard ratio splines for time to **death**, compared to median (73.9%) practice percent coded (sequential adjustments)

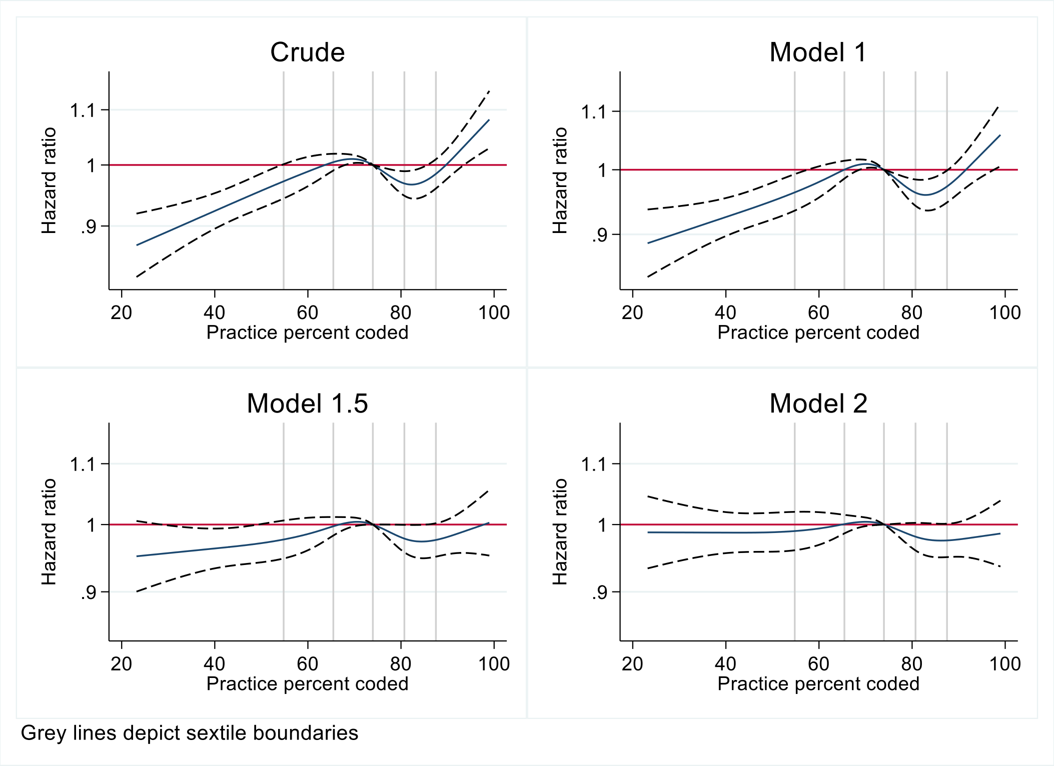

For peer review only

For peer review only - http://bmjopen.bmj.com/site/about/guidelines.xhtml

Supplementary Figure 20. Hazard ratio splines for time to **death**, compared to median (64.9%) practice percent coded, in **CKD stage 3a only** (sequential adjustments)

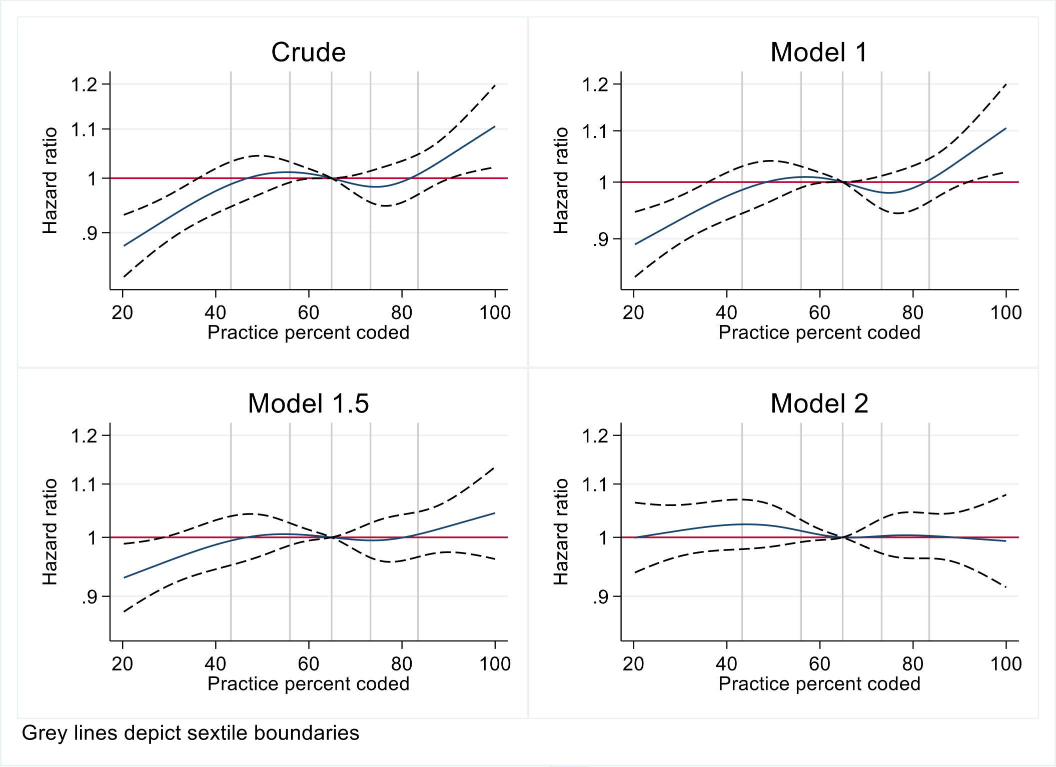

Supplementary Figure 21. Hazard ratio splines for time to **death**, compared to median (87.9%) practice percent coded, in **CKD stages 3b-5 only** (sequential adjustments)

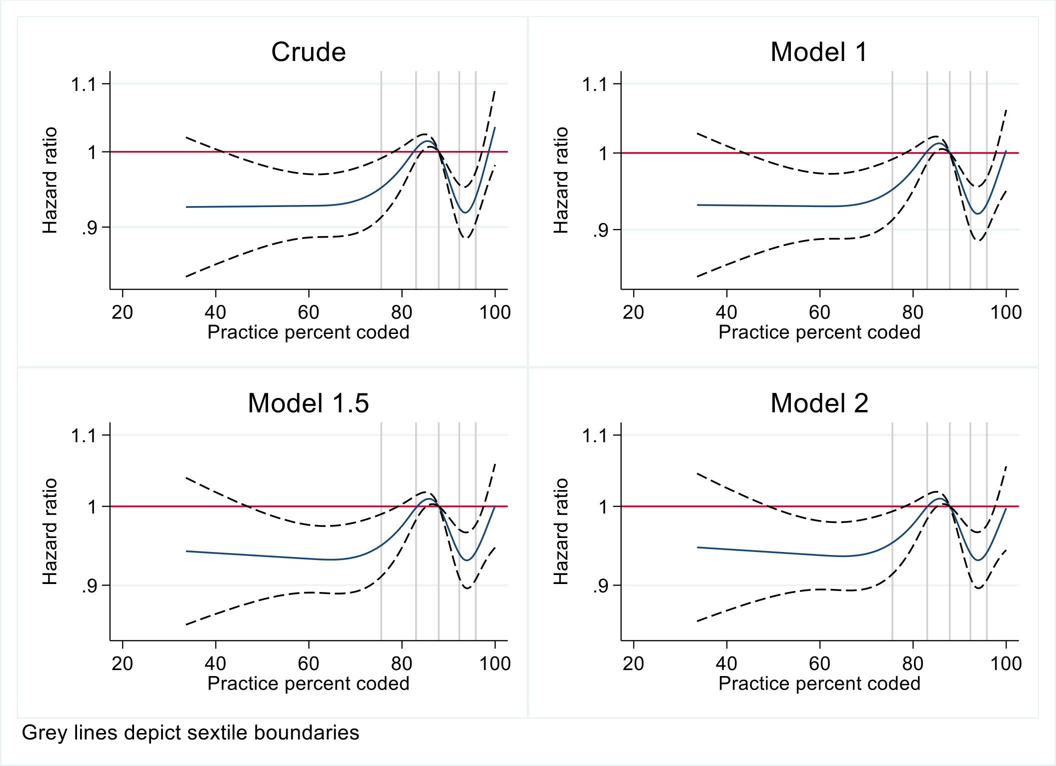

For peer review only - http://bmjopen.bmj.com/site/about/guidelines.xhtml

Supplementary Figure 22. Hazard ratio splines for time to **death**, compared to median (78.6%) practice percent coded, in **diabetes only** (sequential adjustments)

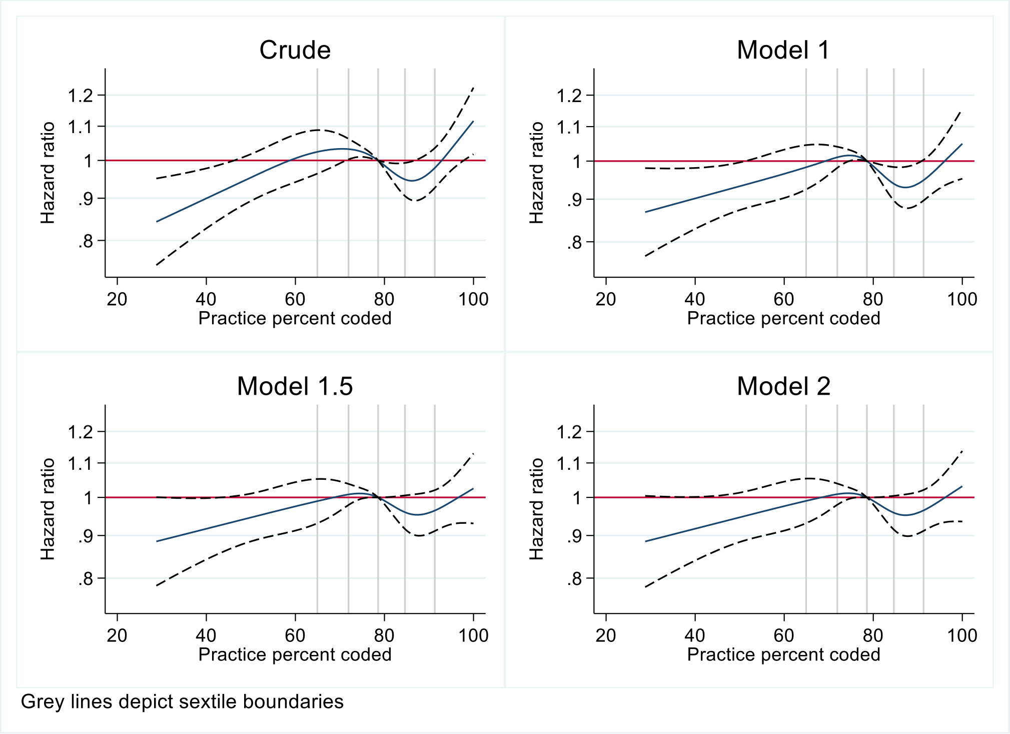

Supplementary Figure 23. Hazard ratio splines for time to **death**, compared to median (71.4%) practice percent coded, in **no diabetes** only (sequential adjustments)

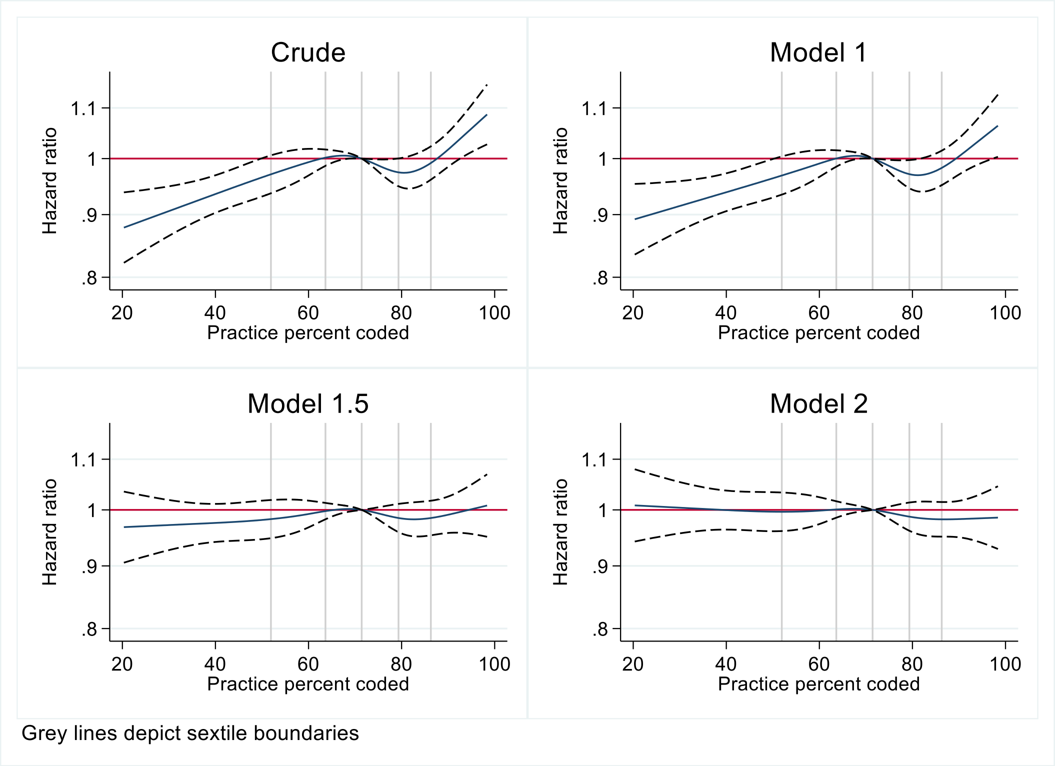

For peer review only - http://bmjopen.bmj.com/site/about/guidelines.xhtml

Supplementary Table 5. Fully adjusted hazard ratios (95% CI) per 10% increase in practice percent coded CKD for each outcome analysed, overall and by subgroup

	CV events	HF	Death
**All CKD**	0.973 (0.955, 0.991)*	0.936 (0.905, 0.967)*	0.999 (0.986, 1.011)
**CKD stage 3a**	0.985 (0.964, 1.007)	0.950 (0.911, 0.992)*	N/A**
**CKD stage 3b-5**	0.935 (0.893, 0.980)*	0.913 (0.846, 0.986)*	N/A**
**Diabetes**	0.963 (0.921, 1.006)	0.975 (0.906, 1.049)	N/A**
**No diabetes**	0.970 (0.948, 0.991)*	0.915 (0.879, 0.953)*	N/A**

Analysis not performed for outcomes and subgroups where assumption of linearity not supported by visual checks. (Descriptive likelihood ratio tests confirmed improved fit for linear term over 5-knot spline model in model 2 adjusted analyses, based on p-value threshold of 0.05, for all reported results, providing further justification for linearity assumption.)
Example interpretation: An adjusted hazard ratio of 0.973 for CV events represents a 6.4% reduction in the rate of CV hospitalisations for each 10% increase in practice CKD coding.

Supplementary Figure 24. Hazard ratio slope estimates (and 95% CI) for CV hospitalisations, HF hospitalisations and deaths, assuming linear differences in practice CKD coding performance compared to median practice coding, in the overall CKD population and within subgroups. *Analyses were carried out in the middle 4 sextiles of practices only (the two-thirds of most typically performing practices), with adjustment for all practice factors (model 2)*

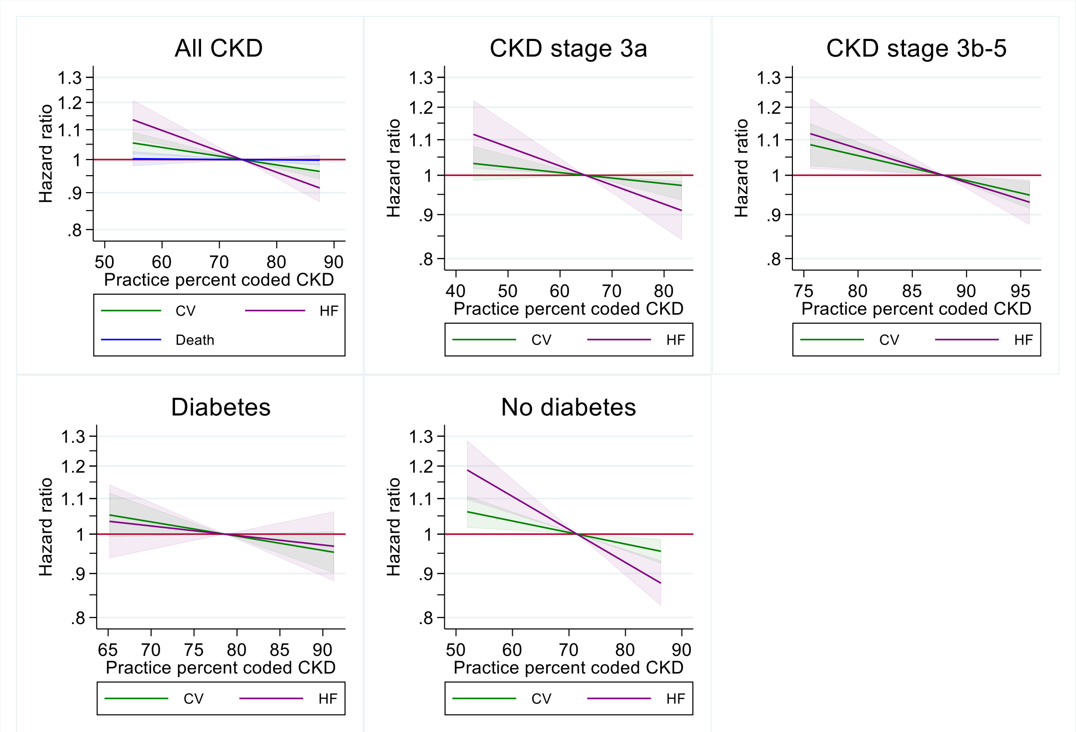

Note: X-scales differ between subgroups, covering middle 4 sextiles within each subgroup

For peer review only - http://bmjopen.bmj.com/site/about/guidelines.xhtml

Supplementary Table 6. Adjusted hazard ratios for the association between practice behaviour variables and **AKI** events, sorted by point estimate

Practice behaviour	HR (95% CI)
Percent ACR/PCR test in last year in CKD (>58.7%)	0.959 (0.935, 0.984)*
Percent flu vaccination in last year in CKD (>78.8%)	0.965 (0.940, 0.990)*
Percent usage of ACEi/ARBs in hypertension (>76.6%)	0.967 (0.944, 0.990)*
Percent usage of statins in CVD (>93.0%)	0.969 (0.944, 0.995)*
Percent usage of statins in diabetes (>84.1%)	0.989 (0.964, 1.015)
Percent pneumococcus vaccination in past 5 years in CKD stages 4-5 (>12.5%)	1.008 (0.985, 1.032)
Percent meeting blood pressure target in last year in CKD (>57.8%)	1.030 (1.004, 1.055)*

Supplementary Table 7. Adjusted hazard ratios for the association between practice behaviour variables and **HF** events, sorted by point estimate

Practice behaviour	HR (95% CI)
Percent usage of ACEi/ARBs in hypertension (>76.6%)	0.935 (0.888, 0.984)*
Percent ACR/PCR test in last year in CKD (>58.7%)	0.951 (0.901, 1.004)
Percent flu vaccination in last year in CKD (>78.8%)	0.959 (0.908, 1.013)
Percent meeting blood pressure target in last year in CKD (>57.8%)	0.976 (0.926, 1.028)
Percent usage of statins in CVD (>93.0%)	0.985 (0.932, 1.042)
Percent pneumococcus vaccination in past 5 years in CKD stages 4-5 (>12.5%)	1.023 (0.973, 1.075)
Percent usage of statins in diabetes (>84.1%)	1.040 (0.984, 1.100)

Supplementary Table 8. Adjusted hazard ratios for the association between practice behaviour variables and **deaths**, sorted by point estimate with descriptive p-values

Practice behaviour	HR (95% CI)
Percent usage of statins in CVD (>93.0%)	0.950 (0.930, 0.970)*
Percent usage of ACEi/ARBs in hypertension (>76.6%)	0.965 (0.947, 0.984)*
Percent usage of statins in diabetes (>84.1%)	0.971 (0.951, 0.991)*
Percent pneumococcus vaccination in past 5 years in CKD stages 4-5 (>12.5%)	1.002 (0.983, 1.020)
Percent flu vaccination in last year in CKD (>78.8%)	1.007 (0.987, 1.027)
Percent meeting blood pressure target in last year in CKD (>57.8%)	1.010 (0.992, 1.030)
Percent ACR/PCR test in last year in CKD (>58.7%)	1.012 (0.992, 1.033)

For peer review only - http://bmjopen.bmj.com/site/about/guidelines.xhtml

Supplementary Figure 25. Results from prior NCKDA analyses, copied from manuscript reference [12]: Hazard ratios for CV events, AKI and mortality in uncoded vs coded patients, adjusted for patient age, sex, and presence of coded diabetes, hypertension and CVD, stratified by latest eGFR measurement, among patients with biochemical evidence of CKD

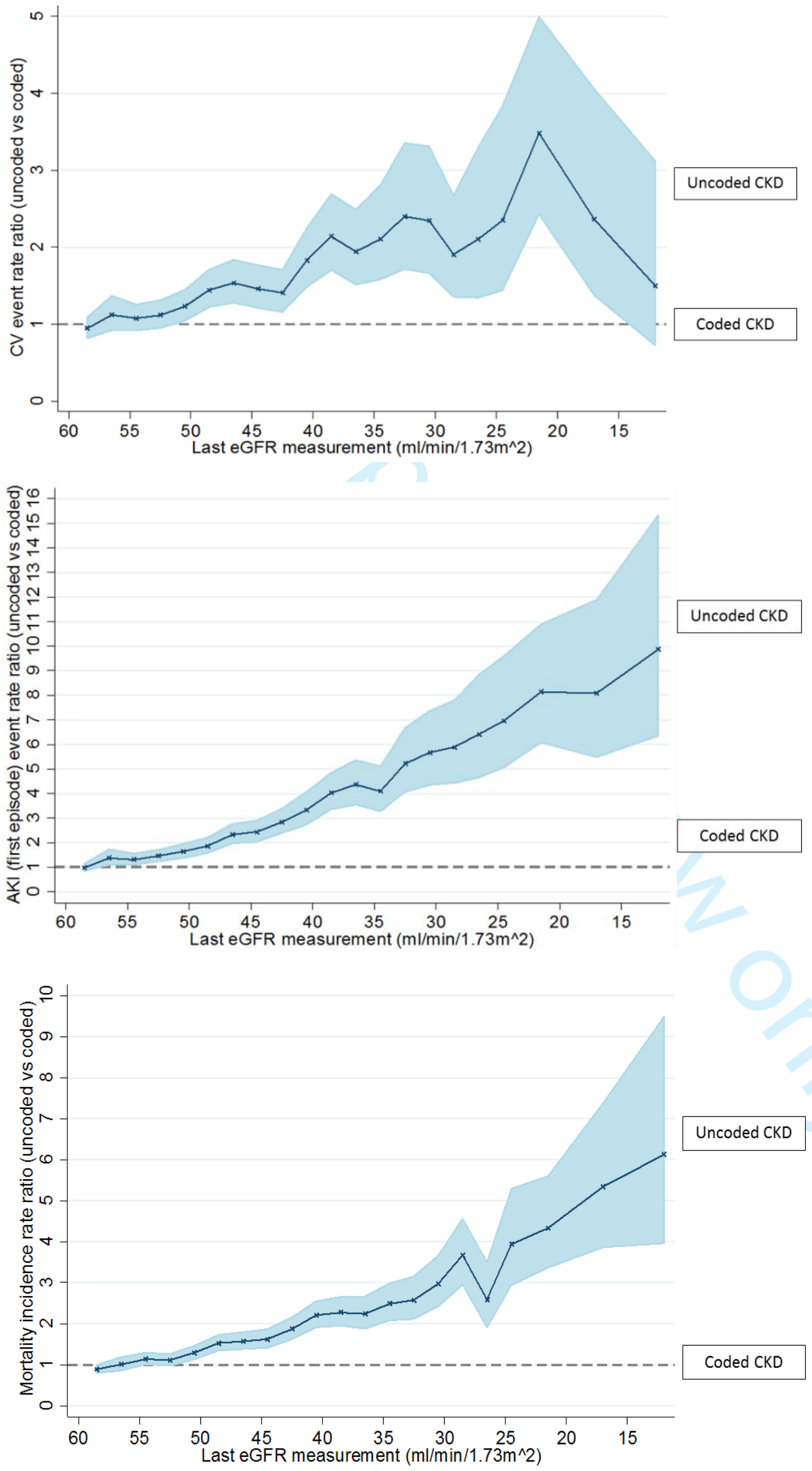

For peer review only - http://bmjopen.bmj.com/site/about/guidelines.xhtml

Supplementary Information 3. Reporting of studies Conducted using Observational Routinely-collected Data (RECORD) checklist

Section/Topic	Item #	Recommendation	Reported on page #
**Title and abstract**	1	(*a*) Indicate the study's design with a commonly used term in the title or the abstract	1, 2
		(*b*) Provide in the abstract an informative and balanced summary of what was done and what was found	2
		RECORD 1.1: The type of data used should be specified in the title or abstract. When possible, the name of the databases used should be included.	2
		RECORD 1.2: If applicable, the geographic region and timeframe within which the study took place should be reported in the title or abstract.	2
		RECORD 1.3: If linkage between databases was conducted for the study, this should be clearly stated in the title or abstract.	2
**Introduction**			
Background/rationale	2	Explain the scientific background and rationale for the investigation being reported	5-6
Objectives	3	State specific objectives, including any prespecified hypotheses	6
**Methods**			
Study design	4	Present key elements of study design early in the paper	6
Setting	5	Describe the setting, locations, and relevant dates, including periods of recruitment, exposure, follow-up, and data collection	6-8
Participants	6	(*a*) Give the eligibility criteria, and the sources and methods of selection of participants. Describe methods of follow-up	6-8
		(*b*) For matched studies, give matching criteria and number of exposed and unexposed	N/A
		RECORD 6.1: The methods of study population selection (such as codes or algorithms used to identify subjects) should be listed in detail. If this is not possible, an explanation should be provided.	7
		RECORD 6.2: Any validation studies of the codes or algorithms used to select the population should be referenced. If validation was conducted for this study and not published elsewhere, detailed methods and results should be provided.	N/A
		RECORD 6.3: If the study involved linkage of databases, consider use of a flow diagram or other graphical display to demonstrate the data linkage process, including the number of individuals with linked data at each stage.	6 (described only, no diagram)

For peer review only - http://bmjopen.bmj.com/site/about/guidelines.xhtml

Variables	7	Clearly define all outcomes, exposures, predictors, potential confounders, and effect modifiers. Give diagnostic criteria, if applicable	7-10; Supplementary Table 1
		RECORD 7.1: A complete list of codes and algorithms used to classify exposures, outcomes, confounders, and effect modifiers should be provided. If these cannot be reported, an explanation should be provided.	7-10; Supplementary Table 1
Data sources/ measurement	8*	For each variable of interest, give sources of data and details of methods of assessment (measurement). Describe comparability of assessment methods if there is more than one group	6-10
Bias	9	Describe any efforts to address potential sources of bias	10-11
Study size	10	Explain how the study size was arrived at	7
Quantitative variables	11	Explain how quantitative variables were handled in the analyses. If applicable, describe which groupings were chosen and why	8-12
Statistical methods	12	(*a*) Describe all statistical methods, including those used to control for confounding	10-12
		(*b*) Describe any methods used to examine subgroups and interactions	11
		(*c*) Explain how missing data were addressed	7-11
		(*d*) If applicable, explain how loss to follow-up was addressed	8, 10
		(*e*) Describe any sensitivity analyses	11
		RECORD 12.1: Authors should describe the extent to which the investigators had access to the database population used to create the study population.	6-7
		RECORD 12.2: Authors should provide information on the data cleaning methods used in the study.	7-10
		RECORD 12.3: State whether the study included person-level, institutional-level, or other data linkage across two or more databases. The methods of linkage and methods of linkage quality evaluation should be provided.	6
**Results**			
Participants	13*	(a) Report numbers of individuals at each stage of study—eg numbers potentially eligible, examined for eligibility, confirmed eligible, included in the study, completing follow-up, and analysed	12, 14, 17
		(b) Give reasons for non-participation at each stage	12, 17; Also see methods p.6-8
		(c) Consider use of a flow diagram	12

For peer review only

		RECORD 13.1: Describe in detail the selection of the persons included in the study (i.e., study population selection) including filtering based on data quality, data availability and linkage. The selection of included persons can be described in the text and/or by means of the study flow diagram.	7, 12
Descriptive data	14*	(a) Give characteristics of study participants (eg demographic, clinical, social) and information on exposures and potential confounders	14
		(b) Indicate number of participants with missing data for each variable of interest	See methods p.6-10
		(c) Summarise follow-up time (eg, average and total amount)	14, 17
Outcome data	15*	Report numbers of outcome events or summary measures over time	17; Also Sup. Figures 2-3, Sup. Table 2
Main results	16	(*a*) Give unadjusted estimates and, if applicable, confounder-adjusted estimates and their precision (eg, 95% confidence interval). Make clear which confounders were adjusted for and why they were included	17-19
		(*b*) Report category boundaries when continuous variables were categorized	14, 17-19
		(*c*) If relevant, consider translating estimates of relative risk into absolute risk for a meaningful time period	17-18
Other analyses	17	Report other analyses done—eg analyses of subgroups and interactions, and sensitivity analyses	17-19
**Discussion**			
Key results	18	Summarise key results with reference to study objectives	19
**Limitations**	19	Discuss limitations of the study, taking into account sources of potential bias or imprecision. Discuss both direction and magnitude of any potential bias	19, 21
		RECORD 19.1: Discuss the implications of using data that were not created or collected to answer the specific research question(s). Include discussion of misclassification bias, unmeasured confounding, missing data, and changing eligibility over time, as they pertain to the study being reported.	20-21
Interpretation	20	Give a cautious overall interpretation of results considering objectives, limitations, multiplicity of analyses, results from similar studies, and other relevant evidence	20-22
Generalisability	21	Discuss the generalisability (external validity) of the study results	20-21
**Other information**			

For peer review only

Funding	22	Give the source of funding and the role of the funders for the present study and, if applicable, for the original study on which the present article is based	23
Accessibility of protocol, raw data, and programming code		RECORD 22.1: Authors should provide information on how to access any supplemental information such as the study protocol, raw data, or programming code.	24; Supplementary materials

*Information should be provided separately for exposed and unexposed groups in cohort studies

For peer review only

Supplementary Information 3. Reporting of studies Conducted using Observational Routinely-collected Data (RECORD) checklist

Section/Topic	Item #	Recommendation	Reported on page #
**Title and abstract**	1	(*a*) Indicate the study's design with a commonly used term in the title or the abstract	1, 2
		(*b*) Provide in the abstract an informative and balanced summary of what was done and what was found	2
		RECORD 1.1: The type of data used should be specified in the title or abstract. When possible, the name of the databases used should be included.	2
		RECORD 1.2: If applicable, the geographic region and timeframe within which the study took place should be reported in the title or abstract.	2
		RECORD 1.3: If linkage between databases was conducted for the study, this should be clearly stated in the title or abstract.	2
**Introduction**			
Background/rationale	2	Explain the scientific background and rationale for the investigation being reported	5-6
Objectives	3	State specific objectives, including any prespecified hypotheses	6
**Methods**			
Study design	4	Present key elements of study design early in the paper	6
Setting	5	Describe the setting, locations, and relevant dates, including periods of recruitment, exposure, follow-up, and data collection	6-8
Participants	6	(*a*) Give the eligibility criteria, and the sources and methods of selection of participants. Describe methods of follow-up	6-8
		(*b*) For matched studies, give matching criteria and number of exposed and unexposed	N/A
		RECORD 6.1: The methods of study population selection (such as codes or algorithms used to identify subjects) should be listed in detail. If this is not possible, an explanation should be provided.	7
		RECORD 6.2: Any validation studies of the codes or algorithms used to select the population should be referenced. If validation was conducted for this study and not published elsewhere, detailed methods and results should be provided.	N/A
		RECORD 6.3: If the study involved linkage of databases, consider use of a flow diagram or other graphical display to demonstrate the data linkage process, including the number of individuals with linked data at each stage.	6 (described only, no diagram)

For peer review only - http://bmjopen.bmj.com/site/about/guidelines.xhtml

Variables	7	Clearly define all outcomes, exposures, predictors, potential confounders, and effect modifiers. Give diagnostic criteria, if applicable	7-10; Supplementary Table 1
		RECORD 7.1: A complete list of codes and algorithms used to classify exposures, outcomes, confounders, and effect modifiers should be provided. If these cannot be reported, an explanation should be provided.	7-10; Supplementary Table 1
Data sources/ measurement	8*	For each variable of interest, give sources of data and details of methods of assessment (measurement). Describe comparability of assessment methods if there is more than one group	6-10
Bias	9	Describe any efforts to address potential sources of bias	10-11
Study size	10	Explain how the study size was arrived at	7
Quantitative variables	11	Explain how quantitative variables were handled in the analyses. If applicable, describe which groupings were chosen and why	8-12
Statistical methods	12	(*a*) Describe all statistical methods, including those used to control for confounding	10-12
		(*b*) Describe any methods used to examine subgroups and interactions	11
		(*c*) Explain how missing data were addressed	7-11
		(*d*) If applicable, explain how loss to follow-up was addressed	8, 10
		(*e*) Describe any sensitivity analyses	11
		RECORD 12.1: Authors should describe the extent to which the investigators had access to the database population used to create the study population.	6-7
		RECORD 12.2: Authors should provide information on the data cleaning methods used in the study.	7-10
		RECORD 12.3: State whether the study included person-level, institutional-level, or other data linkage across two or more databases. The methods of linkage and methods of linkage quality evaluation should be provided.	6
**Results**			
Participants	13*	(a) Report numbers of individuals at each stage of study—eg numbers potentially eligible, examined for eligibility, confirmed eligible, included in the study, completing follow-up, and analysed	12, 14, 17
		(b) Give reasons for non-participation at each stage	12, 17; Also see methods p.6-8
		(c) Consider use of a flow diagram	12

For peer review only

		RECORD 13.1: Describe in detail the selection of the persons included in the study (i.e., study population selection) including filtering based on data quality, data availability and linkage. The selection of included persons can be described in the text and/or by means of the study flow diagram.	7, 12
Descriptive data	14*	(a) Give characteristics of study participants (eg demographic, clinical, social) and information on exposures and potential confounders	14
		(b) Indicate number of participants with missing data for each variable of interest	See methods p.6-10
		(c) Summarise follow-up time (eg, average and total amount)	14, 17
Outcome data	15*	Report numbers of outcome events or summary measures over time	17; Also Sup. Figures 2-3, Sup. Table 2
Main results	16	(*a*) Give unadjusted estimates and, if applicable, confounder-adjusted estimates and their precision (eg, 95% confidence interval). Make clear which confounders were adjusted for and why they were included	17-19
		(*b*) Report category boundaries when continuous variables were categorized	14, 17-19
		(*c*) If relevant, consider translating estimates of relative risk into absolute risk for a meaningful time period	17-18
Other analyses	17	Report other analyses done—eg analyses of subgroups and interactions, and sensitivity analyses	17-19
**Discussion**			
Key results	18	Summarise key results with reference to study objectives	19
**Limitations**	19	Discuss limitations of the study, taking into account sources of potential bias or imprecision. Discuss both direction and magnitude of any potential bias	19, 21
		RECORD 19.1: Discuss the implications of using data that were not created or collected to answer the specific research question(s). Include discussion of misclassification bias, unmeasured confounding, missing data, and changing eligibility over time, as they pertain to the study being reported.	20-21
Interpretation	20	Give a cautious overall interpretation of results considering objectives, limitations, multiplicity of analyses, results from similar studies, and other relevant evidence	20-22
Generalisability	21	Discuss the generalisability (external validity) of the study results	20-21
**Other information**			

For peer review only

For peer review only - http://bmjopen.bmj.com/site/about/guidelines.xhtml

Funding	22	Give the source of funding and the role of the funders for the present study and, if applicable, for the original study on which the present article is based	23
Accessibility of protocol, raw data, and programming code		RECORD 22.1: Authors should provide information on how to access any supplemental information such as the study protocol, raw data, or programming code.	24; Supplementary materials

*Information should be provided separately for exposed and unexposed groups in cohort studies

For peer review only

For peer review only - http://bmjopen.bmj.com/site/about/guidelines.xhtml
